# Meta-analysis of niacin and NAD metabolite treatment in infectious disease animal studies suggests benefit but requires confirmation in clinically relevant models

**DOI:** 10.1038/s41598-025-95735-y

**Published:** 2025-04-12

**Authors:** Colleen S. Curran, Xizhong Cui, Yan Li, Tom Gamble, Junfeng Sun, Samuel Minkove, Alicia A. Livinski, Peter Q. Eichacker, Parizad Torabi-Parizi

**Affiliations:** 1 National Heart Lung and Blood Institute, National Institutes of Health, Bethesda, MD USA; 2 Critical Care Medicine Department, Clinical Center, National Institutes of Health, Bethesda, MD USA; 3 National Institutes of Health Library, Office of Research Services, Office of the Director, National Institutes of Health, Bethesda, MD USA; 4 Critical Care Medicine Branch, National Heart, Lung, and Blood Institute, National Institutes of Health, Bldg 10, Room 2C138, Bethesda, USA

**Keywords:** NAD, Nicotinamide, Niacin, Infection, Sepsis, Bacteria, Virus, Fungus, Animal, Biochemistry, Cell biology, Immunology, Systems biology, Diseases, Medical research, Pathogenesis

## Abstract

Disruption of nicotinamide adenine dinucleotide (NAD) biosynthesis and function during infection may impair host defenses and aggravate inflammatory and oxidative organ injury. Increasingly, studies are investigating whether niacin or NAD metabolite treatment is beneficial in infection and sepsis animal models. We examined whether this preclinical experience supports clinical trials. A systematic review of three data bases was conducted through 2/29/2024 and a meta-analysis was performed comparing niacin or NAD metabolite treatment to control in adult animal models employing microbial challenges. Fifty-six studies met inclusion criteria, with 24 published after 2019. Most studies employed mouse (n = 40 studies) or rat (n = 12) models and administered either a bacterial toxin (n = 28) or bacterial (n = 19) challenge. Four and three studies employed viral or fungal challenges respectively. Studies investigated an NAD metabolite alone (n = 44), niacin alone (n = 9), or both (n = 3), usually administered before or within 24h after challenge (n = 50). Only three and four studies included standard antimicrobial support or started treatment > 24h after challenge respectively. In similar patterns with differing animal types (p ≥ 0.06), compared to control across those studies investigating the parameter, niacin or NAD treatment decreased the odds ratio of mortality [95% confidence interval (CI)] [0.28 (0.17, 0.49)] and in blood or tissue increased antioxidant levels [standardized mean differences (95%CI)] (SMD) [3.61 (2.20,5.02)] and decreased levels of microbes [− 2.44 (− 3.34, − 1.55)], histologic and permeability organ injury scoring [− 1.62 (− 2.27, − 0.98) and − 1.31(− 1.77, − 0.86) respectively], levels of TNFα, IL-6 and IL-1β [− 2.47 (− 3.30, − 1.64), − 3.17 (− 4.74, − 1.60) and − 8.44 (− 12.4, − 4.5) respectively] and myeloperoxidase (MPO) [− 1.60 (− 2.06, − 1.14)], although with significant, primarily quantitative heterogeneity for each (I^2^ ≥ 53%, p < 0.01) except MPO. Treatment increased blood or tissue NAD^+^ levels and decreased chemical organ injury measures and oxidation markers but differently comparing species (p ≤ 0.05). Only 2 and 9 survival studies described power analyses or animal randomization respectively and no study described treatment or non-histologic outcome measure blinding. Among survival studies, Egger’s analysis (p = 0.002) suggested publication bias. While suggestive, published animal studies do not yet support clinical trials testing niacin and NAD metabolite treatment for infection and sepsis. Animal studies simulating clinical conditions and with randomized, blinded designs are needed to investigate this potentially promising therapeutic approach.

## Introduction

Sepsis is a syndrome characterized by a dysregulated host response to severe bacterial, viral, fungal or parasitic infection that produces life-threatening organ dysfunction. Based on an analysis of data from 1990 to 2017, there were approximately 49 million cases of sepsis worldwide that resulted in roughly 20% of all global deaths^1^. Advanced age, obesity, and comorbidities increase the syndrome’s risk of severity and its economic burden on healthcare systems^2^. Sepsis survivors can be left with chronic medical, psychological, and cognitive morbidities that add to this burden^3,4^. Although eradication of infection with anti-microbial agents and source control interventions are the mainstays of sepsis treatment, there has long been a need for agents that target and protect against injurious components of the dysregulated host response.

**Fig. 1:**
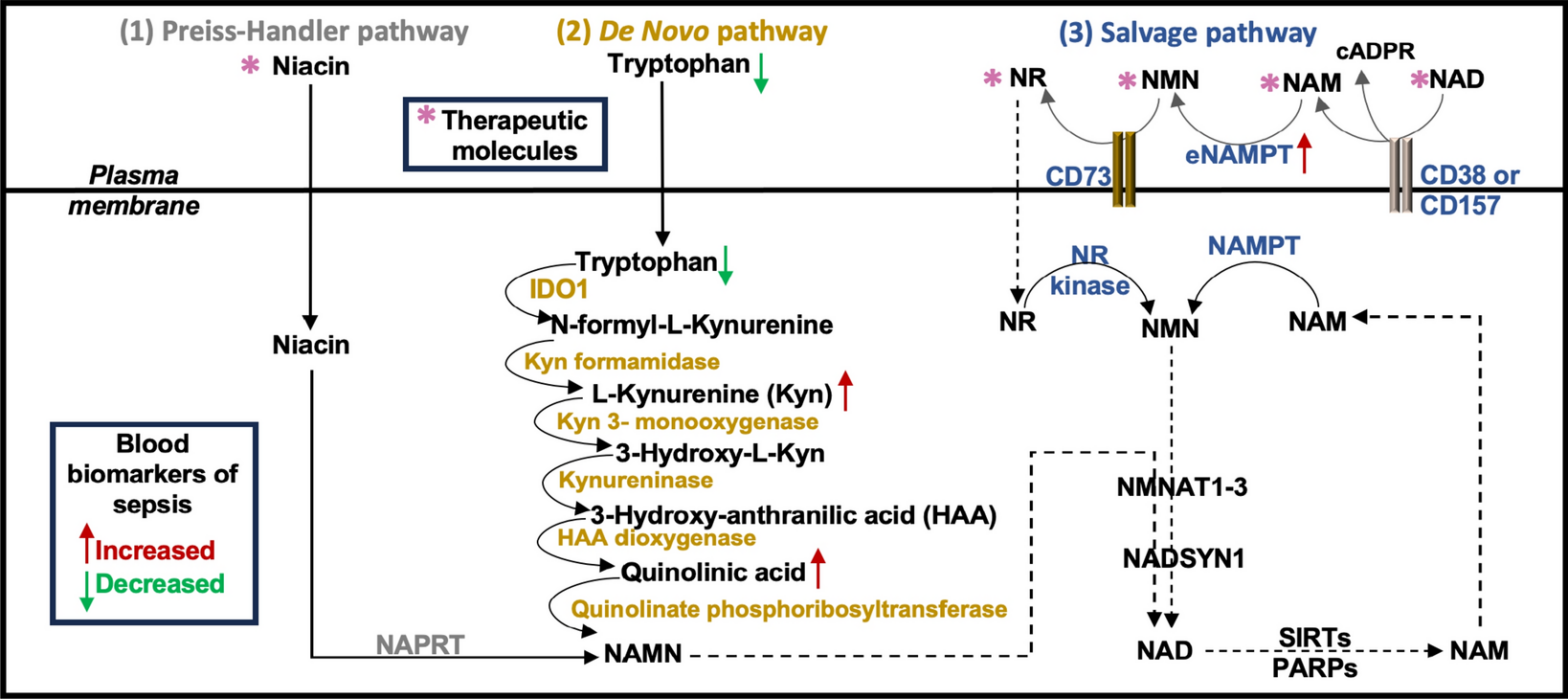
NAD biosynthesis. (1) Preiss-Handler pathway: Niacin transports into cells. Nicotinic acid phosphoribosyltransferase (NAPRT) is the rate-limiting enzyme that forms nicotinic acid mononucleotide (NAMN). Conversion of NAMN to NAD occurs through nicotinamide mononucleotide adenylyltransferases (NMNAT1-3) and NAD synthetase 1 (NADSYN1). (2) De novo pathway: Tryptophan transports into cells. Indoleamine 2, 3-dioxygenase 1 (IDO1) is the rate-limiting enzyme. A series of additional enzymatic reactions produces NAMN, which is also formed in the Preiss-Handler pathway. Conversion of NAMN to NAD occurs through NMNAT1-3 and NADSYN1. (3) Salvage pathway: NAD is catabolized by NADases (CD38, CD157), resulting in the release of nicotinamide (NAM) and cyclic ADP ribose (cADPR) from the cell. Extracellular nicotinamide phosphoribosyltransferase (eNAMPT) transforms NAM into nicotinamide mononucleotide (NMN). Extracellular NMN can be transformed to nicotinamide riboside (NR) by CD73 and NR is transported inside the cell and transformed into NMN by NR kinase. NAMPT also transforms NAM into NMN. Conversion of NMN to NAD occurs through the actions of NMNAT1-3 and NADSYN1.

Nicotinamide (NAM) adenine dinucleotide (NAD) is an enzyme co-factor essential in multiple cellular processes such as the regulation of cell signaling pathways, metabolism, DNA repair, cellular senescence, and immunity^5^. NAD is produced either by catabolism of niacin (the Preiss-Handler pathway) or tryptophan (the de novo pathway) or the recycling of NAD precursors (the salvage pathway)^6^ (Fig. 1). During sepsis, these pathways can be disrupted, resulting in increased blood levels of extracellular nicotinamide phosphoribosyltransferase (eNAMPT)^7^, quinolinic acid^8^, and kynurenine^9^, and lower levels of tryptophan and tissue NAD^+^ levels^10^. These reductions may be associated with impaired host defenses and worsened inflammatory and oxidative tissue injury^11–17^.

Pyrazinamide and isoniazid are two compounds structurally similar to NAM that are highly effective for the treatment *Mycobacterium tuberculosis*^18^. Preclinical studies have suggested that NAD metabolites also exhibit antibacterial effects for non-mycobacterial infections, potentially by altering the activity of neutrophils^19,20^. Studies from our lab have also shown that NAD metabolites inhibit pathogen-associated pro-inflammatory and hypoxic responses in macrophages^6^. These anti-inflammatory macrophages may protect against injury^21^ and promote survival in experimental sepsis^21,22^. Informal review of the literature suggests that there is a substantial published experience investigating the effects of niacin and NAD metabolite treatment in animal infection and sepsis models. To comprehensively examine this experience and provide a possible rationale for clinical study, we performed a systematic review and meta-analysis of controlled preclinical studies examining the effects of niacin and NAD metabolite treatment in adult animal models of infection and sepsis.

## Methods

The Preferred Reporting Items for Systematic Reviews and Meta-Analyses (PRISMA) checklist was used for reporting this systematic review [Supplemental File 1 (SupFile 1)]. A protocol was registered with the International Prospective Register of Systematic Reviews on August 1, 2023 (PROSPERO-CRD42023376816).

### Eligibility criteria

Studies were included that compared the effects of niacin or an NAD metabolite to a control agent in adult animal models that employed a live microbial or a microbial product challenge by any route. Reports not published in English were excluded.

### Information sources and search strategy

Five databases were searched by a biomedical librarian (A.A.L.): Embase, PubMed, Scopus, and Web of Science: BIOSIS and Core Collection from inception through 2/29/2024. Detailed methods and the search strategies used are in SupFiles 2 and 3. The bibliographies of included articles were scanned to identify additional relevant references.

### Selection process

Three authors (C.S.C., T.G., P.Q.E.) used EndNote (Clarivate Analytics) to screen all results from the literature searches. Two authors screened each record using the eligibility criteria at first title and abstract and then full text. Conflicts between authors was resolved by a separate additional author.

### Data extraction and data items

Three authors (C.S.C., S.M., P.Q.E) independently extracted data from reports using tables in Microsoft Excel with formats comparable to those presented in this report. These data included: country and year of publication; species, strain, age and weight of animals; type, dose, route and timing of the microbial or microbial product challenge; type, dose, route and timing of the niacin or NAD metabolite treatment; and parameters of study quality. The following data were also extracted from studies when compared between treatment groups; numbers of surviving and non-surviving animals; NAD^+^ levels; microbe levels; organ injury assessed with either histologic, permeability or liver or kidney chemistry measures; inflammatory cytokines including tumor necrosis factor-α (TNFα), interleukin-1β (IL-1β) or IL-6 levels; antioxidants including superoxide dismutase (SOD), catalase (CAT) or glutathione (GSH); oxidation markers including malondialdehyde (MDA), reactive oxygen species (ROS) or protein carbonyl; and myeloperoxidase (MPO) levels. Analysis included only experimental groups with untreated and treated wild type animals unless genetically modified animals were used in the generation of the infectious challenge model. When numbers or percentages of animals living or dead were not reported in studies presenting survival curves, authors of reports were contacted to obtain these data. If these data were still not available, animal numbers were calculated from presented survival curves and the reported total numbers of animals studied. For all other data, reported mean and median data with variances and/or levels of significance for differences in measures between study groups were recorded. If data were provided in figures alone, authors were also contacted for numerical results. If these data were not available, means or medians with variances were determined from the figures. At least two authors (C.S.C., S.M. or P.Q.E.) independently assessed, and then reached consensus on values for data estimated from figures. If serial survival or other data were reported over time, results at the latest time point were recorded for analysis. When the numbers of animals assigned to study groups were reported as ranges and exact numbers could not be obtained from authors, the lowest number of two consecutive numbers or the mid-number of more than two consecutive numbers were used for analysis.

### Study risk of bias assessment

Two authors (S.M. and P.Q.E) independently assessed each included studies for risk of bias using a modified version of the Systemic Review Centre for Laboratory Animal Experimentation (SYRCLE) grading system^23,24^. Disagreements were resolved by consensus discussion. Studies were examined to determine if the following information was provided: sample size or power calculation; randomization of subjects for treatment; and blinding of treatment allocation and outcome assessments.

### Statistical methods

For survival analysis we estimated the odds ratio (OR) of death. Continuous outcomes were analyzed using standardized mean difference (SMD). Studies were combined using random-effects models^25^. For included studies in which more than one treatment group was compared to a common control group for a measure, the results of these comparisons were pooled (using random-effects models) to provide a single treatment effect for the study, if the level of significance for heterogeneity (I^2^) across comparisons was p > 0.05. If results from such groups within studies differed with a p < 0.05, these groups were included individually in the analysis. Using similar criteria, if a study included more than one experiment comparing treatment to a control group, the results of these experiments were combined or included individually in analysis. The effects of niacin or NAD metabolites were examined across studies employing the same animal type and then across different animal types. Heterogeneity among studies was assessed using the Q statistic and I^2^ value^26^. All analyses were performed using R^27^ (version 4.3.1) packages *meta* (version 6.5–0)^28^. Two-sided p-values ≤ 0.05 were considered significant. Besides animal type, sensitivity analysis examined the influence of the following variables on the effects of treatment on mortality; challenge type [bacterial toxin vs bacteria (including either a single bacteria type, CLP, or feces) vs virus (any type) vs fungus (any type); time of treatment [pre (more than 1d before challenge) vs D0 (within 24h before or after challenge) vs post (more than 1d after challenge)]; and pathway of treatment type [Preiss-Handler (PH) vs salvage]. The effects of treatment on the three antioxidant types were combined in analysis as were the three oxidation marker types. Potential publication bias for studies reporting the effects of treatment on survival was assessed with funnel plot and Egger’s regression. Survival effects were also stratified and analyzed based on quartiles of study size.

## Results

After screening 7,109 retrieved reports and then screening the full text of 130 of the reports, 56 studies were included Supplemental Fig. 1 (SupFig. 1)]. Table 1 summarizes main characteristics of the 56 studies examined^11–17,19–22,29–73^. Supplemental Table 1 (SupTable 1) provides additional information for each study including the sex, weight and age of animals, the regimen of microbe or microbial toxin challenge, and the regimen of niacin or NAD metabolite treatment.

**Table 1:** Summary of microbial challenged animal models tes5ng an NAD metabolite treatment.

Author (year)	Country	Animal	Microbe or microbial toxin challenge	Treatment type	Treatment time	Category of measures analyzable from studies*
Abdel Rasheed (2023)^29^	Egypt	Mouse	LPS (type: *E. Coli*)	Niacin	D0	Organ injury; cytokines
BeJenworth (2014)^19^	Germany, US	Mouse	*Citrobacter roden0um*	NAM	Pre	Microbial clearance; organ injury; MPO
Cao (2023)^30^	China, UK, US	Mouse	Feces	NMN	D0	Survival; microbial clearance; organ injury; cytokines; oxidants; MPO
Chang (1954)^31^	US	Mouse	*Mycobacterium lepraemurium*	NAM	D0 or Post	Survival
Cros (2022)^22^	France, Switzerland	Mouse	CLP	NMN	D0	NAD metabolites; survival; microbial clearance
Doganay (2022)^32^	Turkey	Rat	CLP	NAM	Pre	Cytokines; anZoxidants
Du (2022)^33^	China	Mouse	LPS (type: *E. Coli*)	NMN	Pre	NAD metabolites; survival; organ injury; oxidants; anZoxidants
Duan (2023)^34^	Korea	Mouse	CLP	NAM, NR, Niacin	D0	Survival
Fernandes (2011)^20^	Belgium	Mouse	LPS (type: *E. Coli*)	NAM	D0	Organ injury
Fukuzawa (1997)^35^	Japan	Mouse	LPS (type: *E. Coli*)	NAM	D0	Cytokines
Fulton (1974)^36^	US	Rat	LPS (type: *E. Coli*)	NAM	D0	Survival
Griesman (1979)^37^	US	Mouse	*Proteus mirabilis*	NAM, Niacin	D0	Survival
Guo, W. (2020)^38^	China	Cows	Mastitis (endogenous)	Niacin	Post	Cytokines
Guo, W. (2021)^39^	China	Mouse	LPS (type: UC)	Niacin	Pre	Organ injury; cytokines; MPO
Han (2003)^40^	US	Mouse	LPS (type: *E. Coli*)	NAD^+^	D0	Organ injury
He, D. (2023)^41^	China	Mouse	LPS (type: UC)	NAM, NR, Niacin	D0	Survival
He, M. (2016)^42^	South Korea	Mouse	KSHV	NAM	Post	Survival
He, S. (2022)^11^	China	Mouse	LPS (type: *E. Coli*)	NMN	Pre	NAD metabolites; organ injury
He, S. (2024)^12^	China	Mouse	LPS (type: *E. Coli*)	NMN	D0	NAD metabolites; organ injury; MPO
Hilton (1976)^43^	US	Dog	LPS (type: *E. Coli*)	Niacin	D0	Survival
Hong (2018)^21^	Canada, China, US	Mouse	Feces or LPS (type: UC)	NR	D0	Survival; organ injury; oxidants; MPO
Imaruoka (2019)^44^	Japan	Mouse	LPS (type: UC)	NAM	Pre	Organ injury; cytokines
Iske (2024)^45^	China, Germany, Switzerland, US	Mouse	LPS (type: *E. Coli*) or *E. coli* 0111:B4	NAD^+^	Pre	Survival; organ injury; microbial clearance; cytokines
Izadpanah (2023)^46^	US	Mouse	SARS-CoV-2	NR	D0	NAD metabolites
Jiang (2022)^13^	China	Mouse	SARS-CoV-2	NAD^+^	D0	NAD metabolites; survival; organ injury; microbial clearance
Kao (2007)^47^	Taiwan	Rat	LPS (type: *E. Coli*)	NAM	D0	Organ injury; cytokines
Kwon, W.Y. (2011)^14^	South Korea	Rat	LPS (type: *E. Coli*)	Niacin	D0	NAD metabolites; survival; organ injury; cytokines; oxidants; anZoxidants
Kwon, W.Y. (2016)^48^	South Korea	Rat	LPS (type: *E. Coli*) or CLP	Niacin	D0	Survival; organ injury; cytokines; oxidants; anZoxidants
LeClaire (1996)^49^	USA	Mouse	SEB and LPS (type: *E. Coli*)	NAM	D0	Survival; cytokines
Li, W. (2016)^50^	China	Mouse	HBV (endogenous)	NAM	D0	Microbial clearance
Li, H.R. (2023)^15^	China	Mouse	CLP	NMN	D0	NAD metabolites; survival; cytokines; oxidants; anZoxidants
Liu (2024)^51^	China	Mouse	LPS (type: UC)	NMN	Pre	Cytokines
Micheva-Viteva (2019)^52^	USA	Mouse	*Burkhoderia. pseudomallei*	NAM	Pre	Survival
Mo (2023)^53^	China	Mouse	Human immuno-deficiency virus-1 (HIV-1)	NMN	D0	NAD metabolites; microbial clearance
Nagai (1994)^16^	Japan	Hamster	LPS (type: UC)	Niacin	D0	NAD metabolites; survival; organ injury
Pacl (2023)^54^	South Africa, US	Mouse	*Mycobacterium tuberculosis*	NAM	Post	Organ injury; microbial clearance
Park (2023)^55^	Korea	Rat	CLP	Niacin	D0	Survival; organ injury; cytokines; oxidants; MPO
Pulido (1999)^56^	USA	Rat	LPS (type: *S. typhimurium*)	NAM	D0	Organ injury; MPO
Roboon (2021)^17^	Japan, USA	Mouse	LPS (type: UC)	NR	Pre	NAD metabolites; cytokines
Rodriguez (2018)^57^	Germany, India, Spain, Switzerland, US	Mouse	*Listeria monocytogenes*	NAD^+^	Pre	Survival
Scharte (2003)^58^	Germany	Sheep	LPS (type: *S. typhosa*)	NAM	D0	Organ injury
Selli (2023)^59^	Turkey	Rat	CLP	NR	D0	Oxidants; anZoxidants; MPO
Shaw (1966)^60^	USA	Rat	LPS (type: *E. Coli*)	NAM	D0	Survival
Shi (2017)^61^	China, US	Mouse	LPS (type: UC)	Niacin	D0	Cytokines
Smith (1977)^62^	USA	Mouse	*Staphylococcus aureus*	NAM	D0	Survival
Tian (2023)^63^	China	Mouse	LPS (type: UC)	NMN	UC	Organ injury; cytokines; oxidants; anZoxidants
Umapathy (2012)^64^	USA	Mouse	LPS (type: UC)	NAD^+^	D0	Organ injury; cytokines; MPO
Wray (1998)^65^	UK	Rat	LPS (type: *E. Coli*)	NAM	D0	Survival; organ injury
Wurtele (2010)^66^	Canada	Mouse	*Candida albicans*	NAM	D0	Microbial clearance
Xing (2019)^67^	China	Mouse	*Candida albicans*	NAM	D0	Survival
Xu (2014)^68^	China	Rat	CLP	NAM	Pre	Organ injury, cytokines; oxidants
Yan (2022)^69^	China	Mouse	*Candida albicans*	NAM	D0	Survival; microbial clearance
Ye (2022)^70^	US	Mouse	LPS (type: *E. Coli*) or CLP	NAD^+^	D0	Survival; organ injury; cytokines
Yuan (2012)^71^	China	Mouse	LPS (*type: E. coli*) + /- D-Gal or CLP	NAM	D0	Survival; organ injury; cytokines
Zhao (2023)^72^	China	Mouse	CLP	NR	D0	NAD metabolites; survival; organ injury; cytokines
Zingarelli (1996)^73^	USA	Rat	LPS (type: *E. Coli*)	NAM	D0	NAD metabolites

CLP: cecal ligation and puncture; D0: treatment commenced on the day of infecton; D-Gal: D-galactosamine; HBV: hepatitis B virus; KSHV: Kaposi sarcoma-associated herpesvirus; LPS: lipopolysaccharide (originating from Escherichia coli, Salmonella typhimurium or *Salmonella typhosa)*; MPO: myeloperoxidase; endogenous: animals with existing condition at the time of study; NAD^+^ : nicotinamide adenine dinucleotde; NAM: nicotinamide; NMN: nicotinamide mononucleotide; NR: nicotinamide riboside; Pre: treatment commenced before infection; Post: treatment commenced after infection; SEB: Staphylococcal enterotoxin B; UC: unclear; UK: United Kingdom; US: United States . *Categories of measures included the following parameters: cytokines – serum or tissue levels of TNF-alpha, IL-6, or IL-1-beta; NAD metabolites – blood or tissue niacin, NAD^+^, NAM, NMN, NR levels; microbial clearance – blood or tissue levels of microbes or microbial products; organ injury – histologic (e.g., lung injury score), organ permeability (e.g., lung wet to dry weight raos) or organ functional measures including creatinine, blood urea nitrogen, and/or alanine or aspartate aminotransferases; oxidants – measures of malondialdehyde, reactive oxygen species, protein carbonyl; antioxidants – measures of superoxide dismutase, catalase and/or glutathione; MPO – myeloperoxidase as a measure of neutrophil activation.

While the earliest included study was published in 1954, 38 studies (68%) were published in 2010 or later and 24 studies (43%) have been published since 2019. Most studies employed either mouse (n = 40 studies) or rat (n = 12) models while one each employed hamster, dog, sheep or cow models. Studies included experiments with a bacterial toxin challenge alone (n = 25), live bacterial challenge alone (n = 7), cecal ligation and puncture alone (CLP, n = 8), virus alone (n = 4), fungus alone (n = 3), and bacterial toxin or CLP challenges in separate experiments (n = 3). In one study each, studies included experiments with fecal challenge alone, LPS or fecal challenges, LPS or bacteria, LPS and another bacterial toxin together, endogenous mastitis, or endogenous viral challenge. Nine studies included experiments investigating niacin treatment alone (n = 9) and 44 studies included experiments investigating treatment with one or more NAD metabolites including NAM, nicotinamide mononucleotide (NMN), nicotinamide riboside (NR), or NAD. Three studies included separate experiments or groups investigating niacin and an NAD metabolite. Only three studies, one each in a bacteria^62^, virus^53^ or fungus^69^ challenged model, reported administering a standard anti-microbial type treatment in combination with niacin or an NAD metabolite. Treatment was administered within 24h of the microbe or microbial toxin challenge in 39 studies, more than 24h before or after challenge in 11 and 4 studies respectively, within and after 24h of challenge in separate experiments in 1 study and timing was unclear in 1 study.

**Fig. 2:**
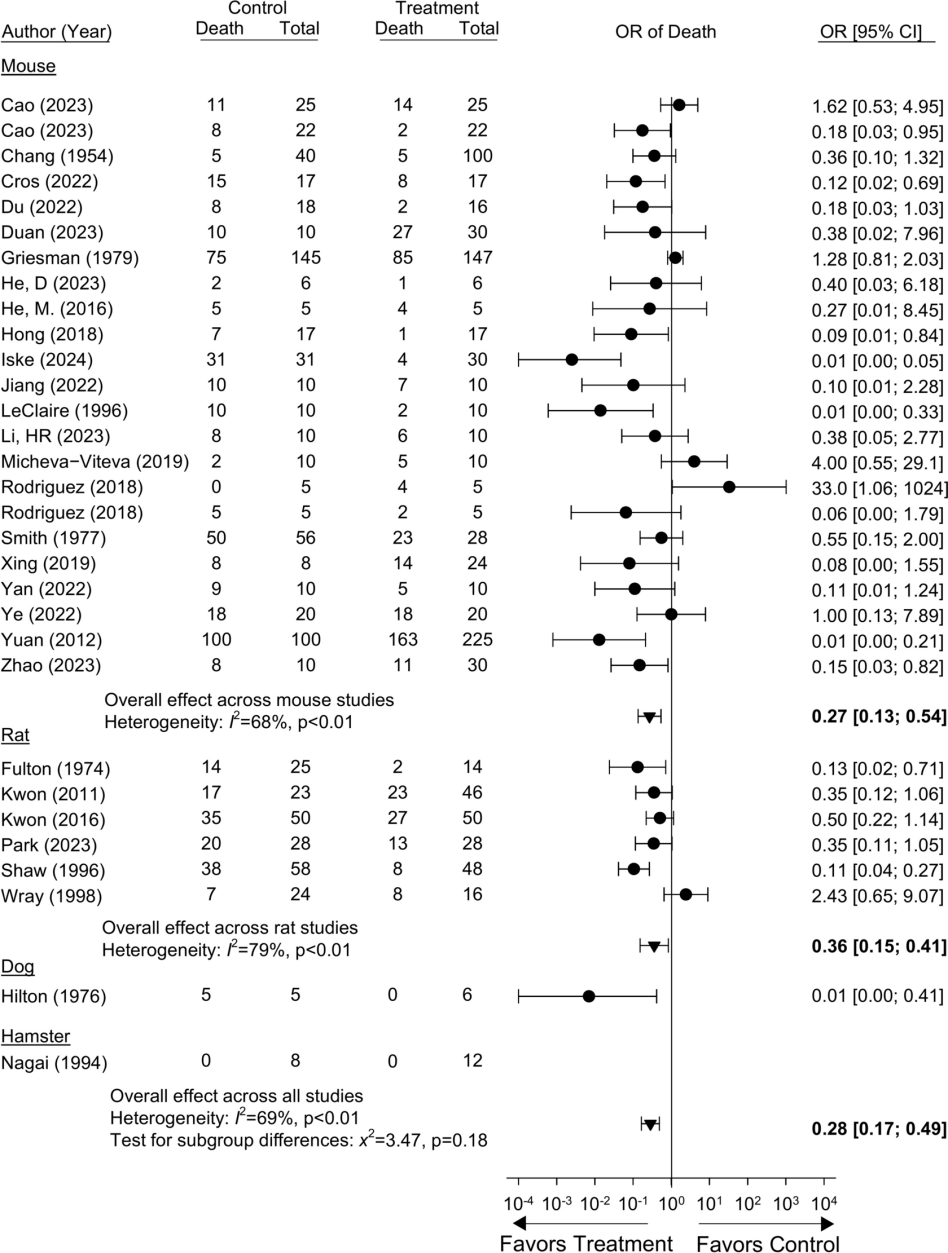
Effects of niacin or nicotinamide adenine dinucleotide (NAD) metabolite treatment versus control on the odds ratio of mortality (95%CIs) (OR) in studies (author, year of publication) conducted in either mouse, rat, dog or hamster models. Shown are the numbers of animals dying and total numbers of animals in the treatment and control groups. Data employed for analysis are shown in Supplemental Table 2. Data from individual experiments that were pooled within studies based on nonsignificant heterogeneity (p ≥ 0.05) comparing the experiments are shown in Supplemental Fig. 2. In two studies (Cao and Rodriguez), results of two experiments could not be combined. In one study (Nagai), there were no deaths in either treatment and control groups and no OR could be calculated. Across the 30 studies or individual experiments with analyzable data, niacin or NAD metabolite treatment was associated with an OR on the side of benefit in 24 (80% of analyzable studies), and in 10 of these, the effects were significant (i.e. the 95%CI was entirely on the side of benefit for treatment). The effects of treatment did not differ significantly comparing the species studied (p = 0.18). Although treatment decreased the overall OR of mortality both within species and across all studies, heterogeneity of effects due primarily to quantitative differences was significant (I^2^ = 69%, p < 0.01). Similar, primarily quantitative heterogeneity, persisted in the overall beneficial effects of treatment on mortality across studies whether stratified based on type of challenge, time of treatment or type of treatment (see results) (SupFigures-14 to -16).

Data from studies employed for meta-analysis of individual measures are presented in SupTables 2 to 13. Analyses conducted to determine whether individual experiments in studies with more than one experiment or treatment group could be combined to determine a single treatment effect for a report are shown in SupFigs 2 to 13 (see “Methods”). Twenty-nine studies presented the effects of treatment on mortality, two of which included experiments or groups that could not be combined and one which reported no mortality in either the control or treatment groups (SupTable 2) (Fig. 2, SupFig. 2). Across the 30 studies or individual experiments with analyzable data, involving mice (n = 23), rats (n = 6) or dogs (n = 1), niacin or NAD metabolite treatment was associated with an odds ratio of mortality (OR, 95% confidence interval [CI]) on the side of reduced mortality (i.e. benefit) in 24 (80% of analyzable studies), and in 10 of these studies, the effects were significant (i.e. the 95%CI was entirely on the side of benefit for treatment). The effects of treatment did not differ significantly comparing the four species studied (p = 0.18). Treatment decreased the overall OR of mortality [0.28 (0.17, 0.49)], although the heterogeneity of effects, due primarily to quantitative differences, was significant (I^2^ = 69%, p < 0.01). Similar, primarily quantitative heterogeneity, persisted in the beneficial effects of treatment on mortality across studies whether stratified based on type of challenge [bacteria, microbial toxin (LPS), virus or fungus], time of treatment (pre, D0, or post) or type of treatment (PH or salvage pathway) (see methods) (SupFigs. 14, 15 and 16). Although D0 treatment and pre-treatment yielded a significant survival benefit, post-treatment only tended to benefit survival in the three studies reported and the response was not significant (SupFig. 15). Moreover, only one study examined treatment across doses of challenge designed to produce different levels of severity^57^.

**Fig. 3:**
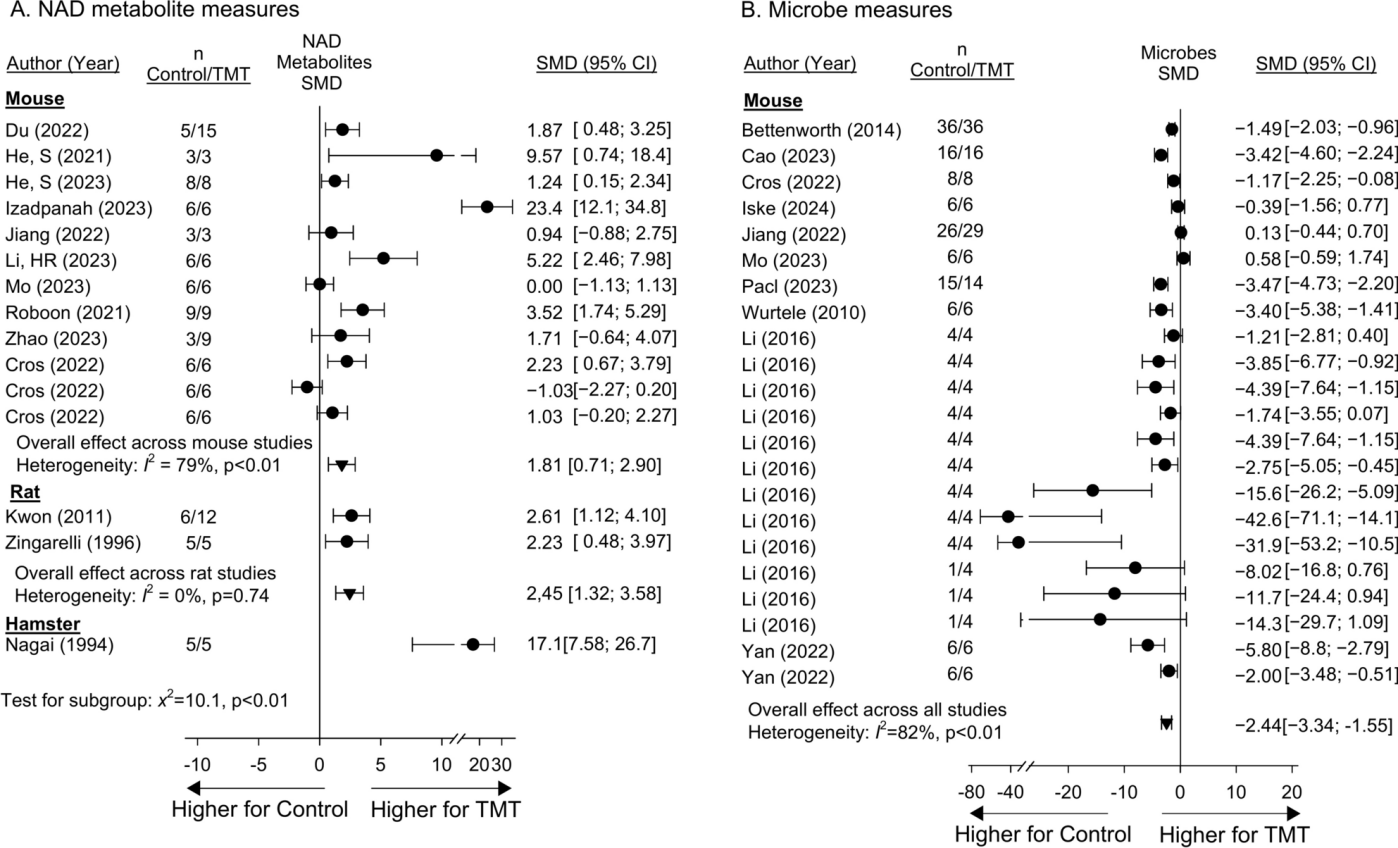
Effects of niacin or nicotinamide adenine dinucleotide (NAD) metabolite treatment (TMT) versus control on the standardized mean difference (95%CIs) (SMD) in NAD metabolite (Panel **A**) and microbe (Panel **B**) measures in blood or tissue in studies (author, year of publication) conducted in either mouse, rat, or hamster models. Animal numbers (n) for study groups are shown. Data employed for analysis are shown in Supplemental Table 2 and 3. Data from individual experiments that were pooled within studies based on nonsignificant heterogeneity (p ≥ 0.05) comparing the experiments are shown in Supplemental Figs. 3 and 4. In one study for NAD metabolite (Cros) and two for microbe (Li and Yan) levels, results of more than one experiment could not be combined. Treatment had effects on the on the side of increasing SMDs in NAD^+^ levels in 14 of 15 studies or experiments (93%) and in 10, these increases were significant (i.e. the 95%CI was entirely on the side of increases with treatment). (Panel **A**). The magnitude of increases differed across species (p < 0.01) and heterogeneity within species with more than one study or experiment was significant for mice (n = 12, I^2^ = 79%, p < 0.01) but not rats (n = 2, I^2^ = 0%, p = 0.74). Treatment had effects on the side of decreasing SMDs for microbe levels in 20 of 22 studies or experiments (91%), and decreases were significant in 14, although there was significant primarily quantitative heterogeneity (I^2^ = 82%, p < 0.01) (Panel **B**).

Thirteen studies reported the effects of niacin or NAD metabolite treatment compared to controls on serum or tissue NAD^+^ levels, one of which included 3 experiments that could not be combined in analysis (SupTable 3) (Fig. 3A, SupFig. 3). Treatment had effects on the side of increasing standardized mean differences (95%CI) (SMD) in NAD^+^ levels in 13 of 15 studies or experiments (93%) and in 10, these increases were significant (i.e. the 95%CI was entirely on the side of increases with treatment). The magnitude of increases differed comparing species (p < 0.01), but treatment increased NAD^+^ levels significantly across studies in mice [n = 10 studies; 1.81 (0.71, 2.90); I^2^ = 79%, p < 0.01], rats [n = 2; 2.45 (1.32, 3.58; I^2^ = 0, p = 0.74] and hamsters [n = 1; 17.1 (7.58, 26.7)]. Ten studies, all in mice and with two including experiments that could not be combined, reported the effects of treatment on blood or tissue microbe levels (SupTable 4) (Fig. 3B, SupFig. 4). Treatment had effects on the side of decreasing SMDs for microbe levels in 20 of 22 studies or experiments (91%), and decreases were significant in 14 and overall [− 2.44 (− 3.34, − 1.55)], although with significant, primarily quantitative, heterogeneity (I^2^ = 82%, p < 0.01).

**Fig. 4:**
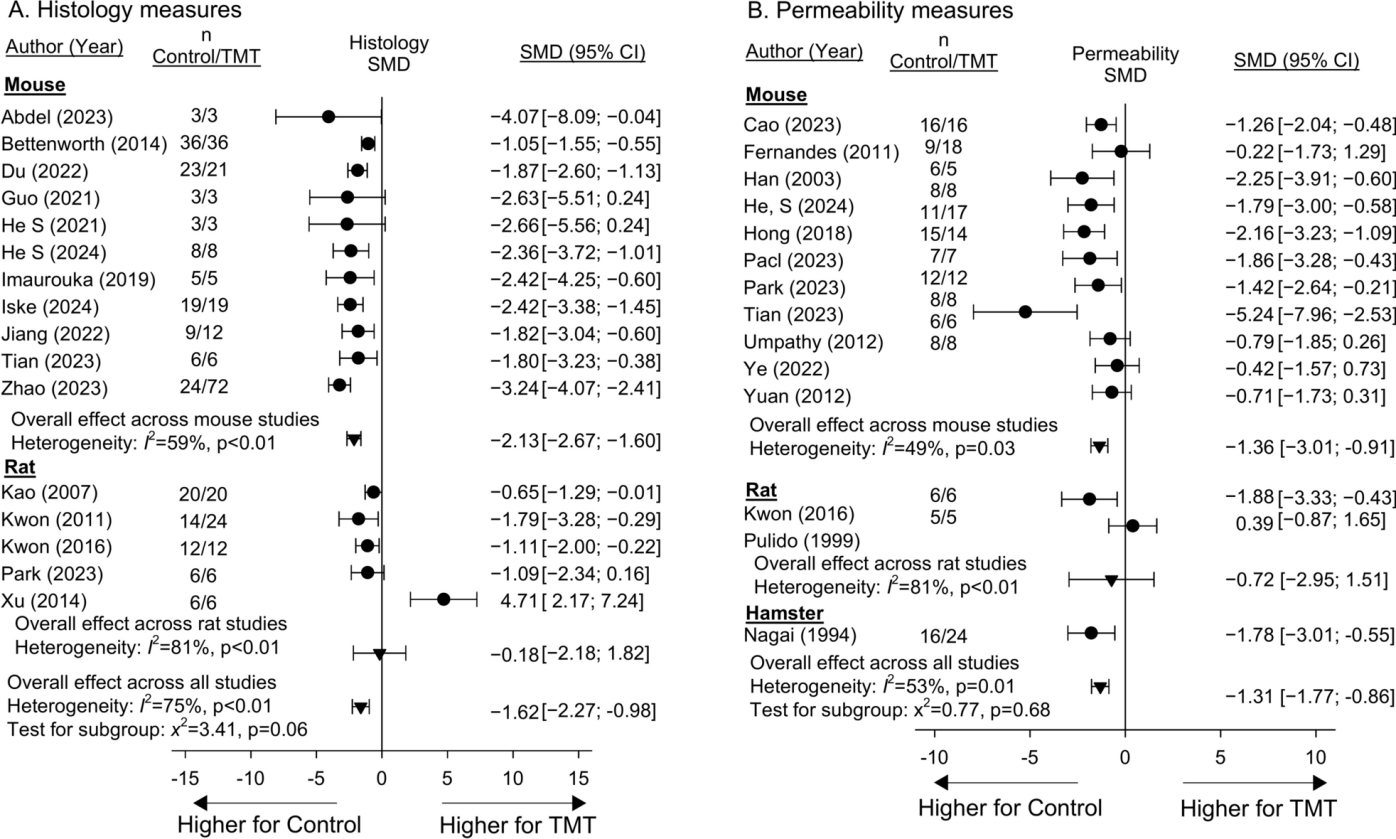
Effects of niacin or nicotinamide adenine dinucleotide (NAD) metabolite treatment (TMT) versus control on the standardized mean difference (95%CIs) (SMD) in histology (Panel **A**) and permeability (Panel **B**) measures in studies (author, year of publication) conducted in either mouse, rat, or hamster models. Animal numbers (n) for study groups are shown. Data employed for analysis are shown in Supplemental Tables 5 and 6. Data from individual experiments that were pooled within studies based on nonsignificant heterogeneity (p ≥ 0.05) comparing the experiments are shown in Supplemental Figs. 5 and 6. Sixteen and 14 studies reported the effects of treatment on histologic or permeability measures respectively and for studies with more than one experiment, results could be combined for each measure. Treatment had effects on the side of decreasing SMDs for histologic and permeability in 15 (94%) and 13 (93%) studies and experiments respectively, and in 12 and 9 respectively, these decreases were significant (i.e. the 95%CI was entirely on the side of decreases with treatment). Effects did not differ across species for either measure (p ≥ 0.06). Although primarily quantitative, there was significant heterogeneity across studies within species as well as overall for histological and permeability measures of organ injury (I^2^ ≥ 53%, p ≤ 0.01).

Sixteen studies reported the effects of niacin or NAD metabolite treatment compared to controls on histologic organ injury measures and 14 studies examined the effects of treatment on permeability measures (SupTables 5 and 6). For studies with more than one experiment, results of experiments could be combined for each of the two measures (SupFigs. 5 and 6). Studies were conducted in mice, rats or hamsters. Eight studies reported the effects of treatment on chemical measures of organ injury (i.e. blood urea nitrogen, creatinine, aspartate amino-transferase and/or alanine amino-transferase) in mice, rats or sheep, but in two studies with more than one experiment, results could not be combined (SupTable 7, SupFig 7). Treatment had effects on the side of decreasing SMDs for histologic, permeability and chemical organ injury in 15 (94%), 13 (93%) and 13 (87%) studies and experiments respectively, and in 12, 9 and 10 respectively, these decreases were significant (Fig. 4A and B, SupFig. 17A). Effects did not differ across species for histology or permeability measures (p ≥ 0.06) and the overall effects of treatment on these measures were significant [− 1.62 (− 2.27, − 0.98) and [− 1.31 (− 1.77, − 0.86)] respectively, although with primarily quantitative heterogeneity for both (I^2^ ≥ 53%, p < 0.01). Effects of treatment on chemical measures did differ (p < 0.01) comparing mice [n = 7; − 2.91 (− 4.24, − 1.58); I^2^ = 72%, p < 0.01], rats [n = 7; − 0.35 (− 2.54, 1.83); I^2^ = 91%, p < 0.01] and sheep (n = 1; 0.34 (− 0.32, 1.00)].

**Fig. 5:**
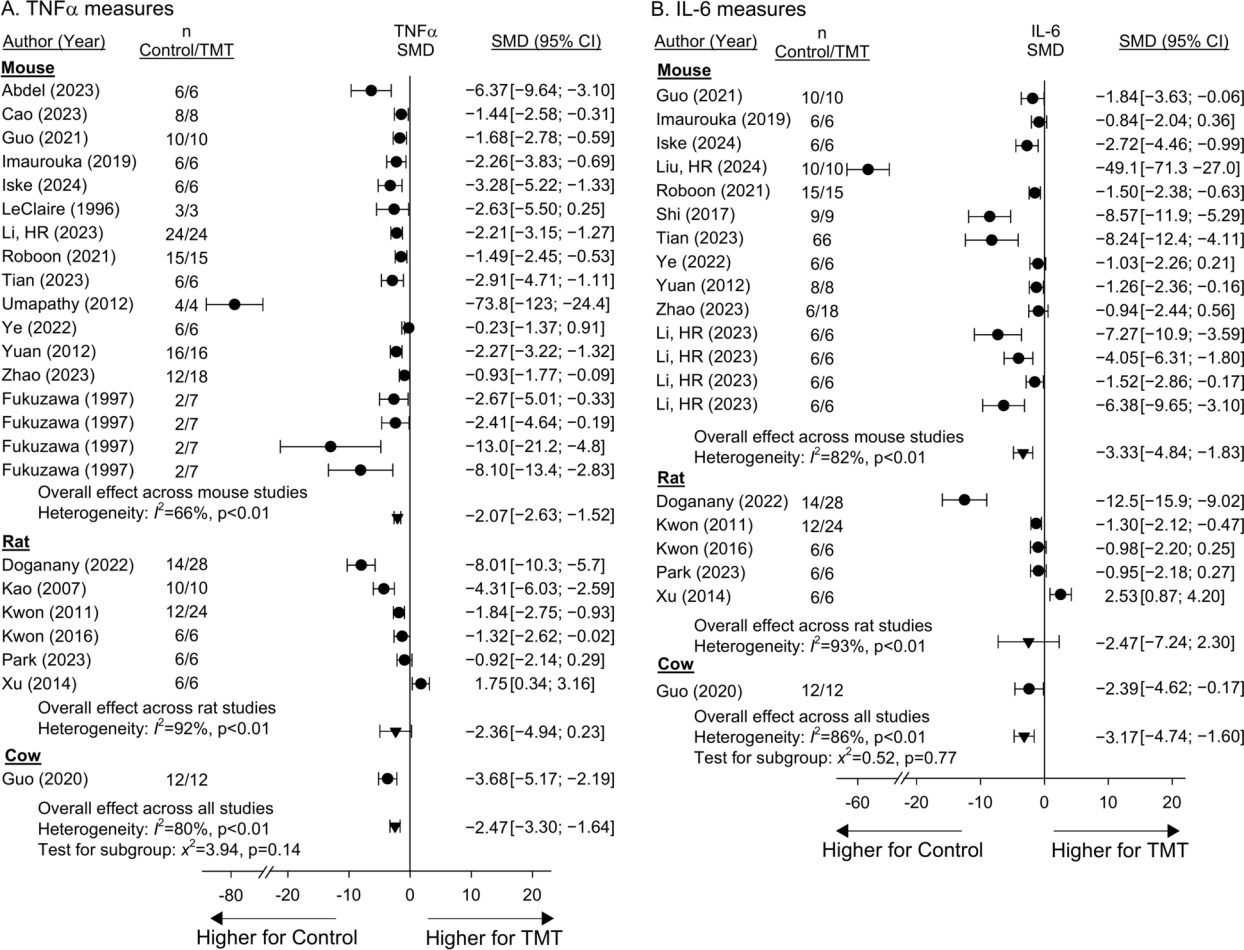
Effects of niacin or nicotinamide adenine dinucleotide (NAD) metabolite treatment (TMT) versus control on the standardized mean difference (95%CIs) (SMD) in tumor necrosis factor-α (TNFα) (Panel **A**) and interleukin-6 (IL-6) (Panel **B**) measures in studies (author, year of publication) conducted in either mouse, rat, or cow models. Animal numbers (n) for study groups are shown. Data employed for analysis are shown in Supplemental Tables 8 and 9. Data from individual regimens of treatment that were pooled within studies based on nonsignificant heterogeneity (p ≥ 0.05) comparing the regimens are shown in Supplemental Figs. 8 and 9. Twenty and 17 studies reported the effects of treatment on TNFα and IL-6 measures respectively and there was one study for each cytokine in which experiments could not be combined for analysis. Treatment had effects on the side of decreasing SMDs for TNFα and IL-6 in 23 (96%) and 19 (95%) studies and experiments respectively, and in 20 and 14 respectively, these decreases were significant (i.e. the 95%CI was entirely on the side of decreases with treatment). Effects did not differ significantly comparing species for either of the cytokines (p ≥ 0.14). However, while primarily quantitative, there was significant heterogeneity across studies within species as well as overall for each cytokine measure (I^2^ ≥ 66%, p < 0.01).

Twenty-one, 17, and 12 studies reported the effects of niacin or NAD metabolite treatment compared to controls on blood or tissue TNFα, IL-6 or IL-1β levels, respectively in mouse, rat or cow models (SupTables 8, 9 and 10). There was one study for each cytokine in which experiments could not be combined for analysis (SupFigures 8, 9 and 10). Treatment had effects on the side of decreasing SMDs for TNFα, IL-6 and IL-1β in 23 (96%), 19 (95%) and 13 (100%) studies and experiments respectively, and in 20, 14 and 12 respectively, these decreases were significant (Fig. 5A and B, SupFig. 17B). Effects did not differ significantly comparing species for any of these cytokines (p ≥ 0.08) and treatment reduced overall SMDs for TNFα [− 2.47 (− 3.30, − 1.64)], IL-6 [− 3.17 (− 4.74, − 1.60)] and IL-1β [− 8.44 (− 12.4, − 4.5)] but with significant primarily quantitative heterogeneity for each cytokine (I^2^ ≥ 80%, p < 0.01).

**Fig. 6:**
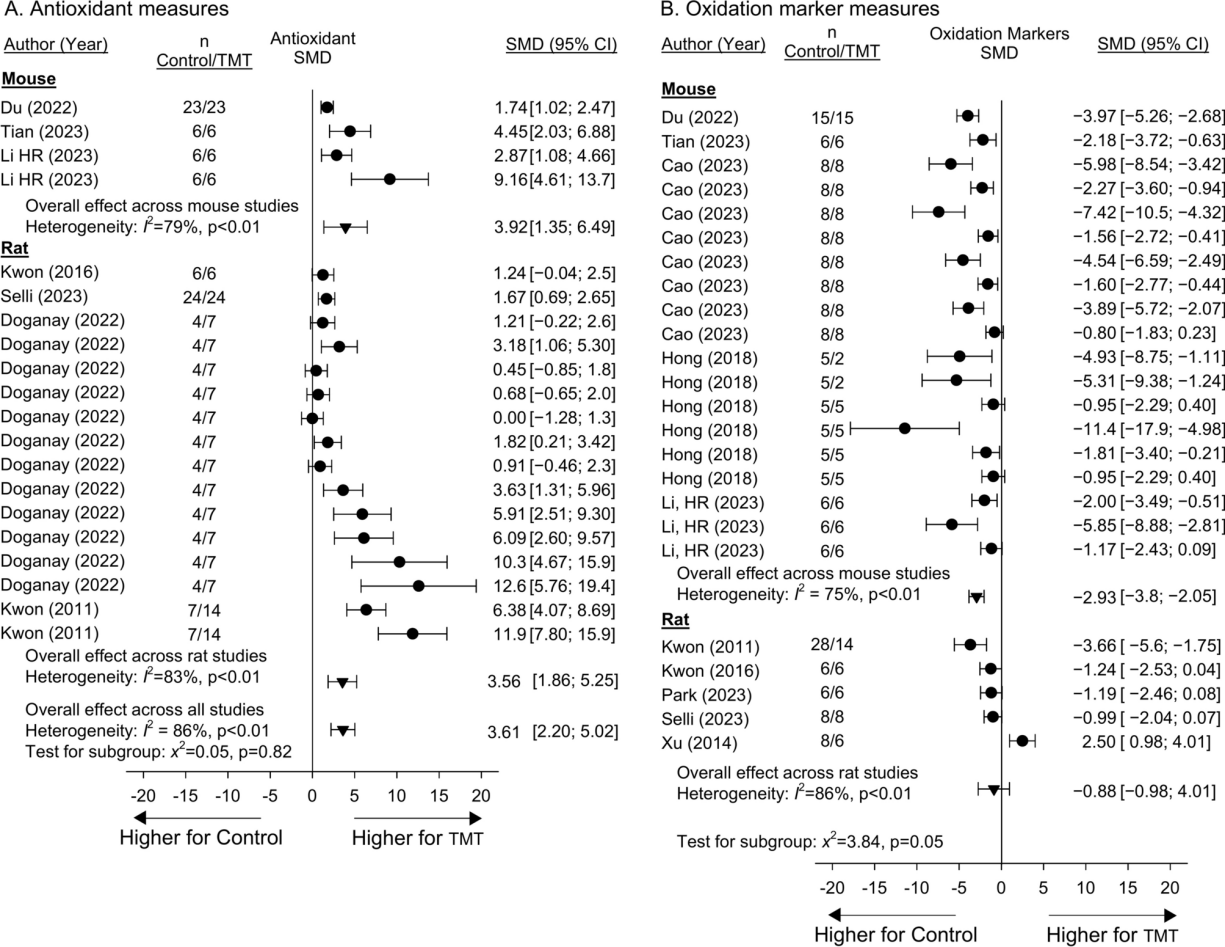
Effects of niacin or nicotinamide adenine dinucleotide (NAD) metabolite treatment (TMT) versus control on the standardized mean difference (95%CIs) (SMD) in antioxidants (superoxide dismutase, catalase or glutathione) (Panel **A**) and oxidation markers (malondialdehyde, reactive oxygen species or protein carbonyl) (Panel **B**) in studies (author, year of publication) conducted in either mouse or rat models. Data employed for analysis are shown in Supplemental Tables 12 and 13. Animal numbers (n) for study groups are shown. Data from individual regimens of treatment that were pooled within studies based on nonsignificant heterogeneity (p ≥ 0.05) comparing the regimens are shown in Supplemental Figs. 11 and 12. Seven and 10 studies reported the effects of treatment on antioxidant and oxidation marker measures respectively and there were 3 studies for each measure respectively in which experiments could not be combined for analysis. Treatment had effects on the side of increasing SMDs for antioxidant levels in 20 of 20 (100%) studies and experiments and decreasing SMDs for oxidation products in 23 of 24 (96%) studies and in 14 and 16 of these respectively, the effects of TMT were significant (i.e. the 95%CI was entirely on the side of decreases with treatment). Effects of TMT on antioxidant levels did not differ significantly (p = 0.82) but did for oxidation markers comparing mouse and rat models (p = 0.05). For both types of measures, there was significant heterogeneity across studies and experiments within species and overall (I^2^ ≥ 75%, p < 0.01).

Seven studies reported the effects of treatment on blood or tissue antioxidants including superoxide dismutase (SOD), catalase (CAT), and/or glutathione (GSH), which were combined in analysis of SMDs (SupTable 11). Three studies included experiments which could not be combined in analysis (SupFig. 11). Treatment had effects on the side of increasing SMDs for antioxidants in 20 of 20 (100%) studies and experiments, and in 14 of these increases were significant. There were no significant differences comparing the effects of treatment in mouse vs. rat models (p = 0.82) and overall treatment increased antioxidants [3.61 (2.20, 5.02)] but with significant primarily quantitative heterogeneity (I^2^ = 86%, p < 0.01) (Fig. 6A). Ten studies reported the effects of niacin or NAD metabolite treatment on blood or tissue oxidation markers including malondialdehyde (MDA), reactive oxygen species (ROS) and/or protein carbonyl which were combined in analysis of SMDs (SupTable 12). Three studies included experiments which could not be combined in analysis (SupFig. 12). Treatment had effects on the side of decreasing SMDs for oxidation markers in 23 of 24 (96%) studies and experiments, and in 16 these decreases were significant (Fig. 6B). Decreases were significant in mice [− 2.93 (− 3.8, − 2.05)] but not rats [− 0.88 (− 0.98, 4.01)] (p = 0.05 comparing species) and there was significant heterogeneity across studies and experiments for both species (I^2^ ≥ 75%, p < 0.01).

Nine studies reported the effects of treatment on myeloperoxidase (MPO) levels (SupTable 13). The effects of treatment could be combined in the two studies with more than one experiment (SupFig. 13). Treatment had effects on the side of decreasing SMDs for MPO in all nine studies (100%) and in 6, decreases were significant (SupFig. 17C). Treatment effects did not differ comparing mouse and rat (p = 0.55) and overall treatment decreased MPO levels [− 1.60 (− 2.06, − 1.14); I^2^ = 30%, p = 0.18].

**Fig. 7:**
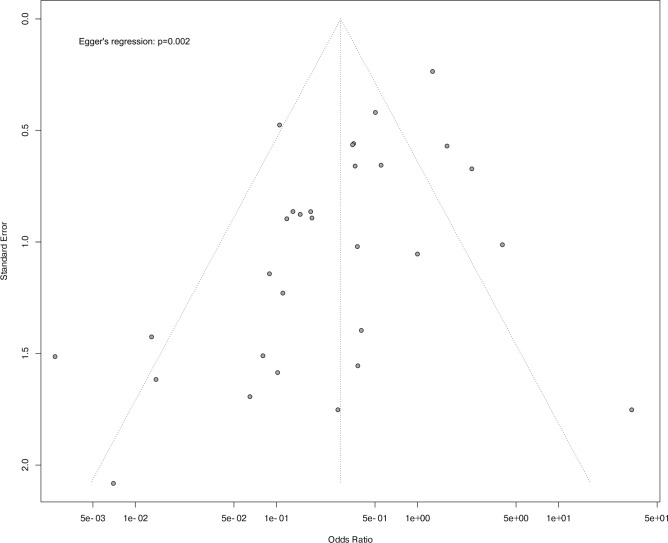
The funnel plot and Egger’s statistic for the 29 studies or experiments providing survival results shown in Fig. 1.

Three of the 56 studies described a sample size or power calculation and 2 of these were among the 29 studies assessing the effects of treatment on mortality (SupTable 14). Thirteen studies described randomizing animals to treatment and 9 of these were among the 29 studies with mortality results. No study described blinding of treatment administration. While 11 of 16 studies described blinding of histology measurements, no study noted blinding of survival or other non-survival type outcomes. The funnel plot and Egger’s analysis (p = 0.002) (Fig. 7) of studies reporting the effects of treatment on survival suggest potential publication bias. However, even when these studies were stratified into quartiles based on study size (SupFig. 18), there was still significant heterogeneity across studies in three of the four quartiles (I^2^ ≥ 50%, p ≤ 0.04).

## Discussion

Despite interest in the potential use of niacin and NAD metabolites as immunomodulatory agents in the treatment of infection and sepsis, this is the first systematic literature review and meta-analysis we are aware of that has examined the controlled preclinical animal experience investigating this question^18,74–76^. Notably, more than 40% of the 56 studies analyzed here were published in 2020 or later suggesting that interest in this question is increasing.

On the one hand, the results of this analysis might be viewed as supportive of this therapeutic approach. While there was significant heterogeneity in the effects of niacin or NAD metabolite treatment across many measures examined, this appeared to be primarily quantitative and not qualitative. In the 29 studies providing survival data, the most frequent outcome reported, treatment was associated with reductions in mortality that were on the side of benefit in 24 studies and significantly beneficial in 10 studies. Supporting this survival benefit, in studies presenting data, treatment was associated with increases in blood or tissue NAD^+^ and antioxidant levels and reductions in microbe, organ injury, inflammatory cytokine, oxidation marker and myeloperoxidase measures. Preclinical in vitro data may further support the potential beneficial immunomodulatory effects of these molecules^6,53,77^. The present findings are also consistent with clinical studies that suggest a therapeutic role for NAD metabolites in patients with *Mycobacterium tuberculosis*, human immunodeficiency virus-1, and SARS-CoV-2-associated acute kidney injury^18,78^.

However, there are weaknesses in this body of data that undermine its usefulness as a basis for the clinical application of niacin or NAD metabolite treatment for infection and sepsis. First, twenty-six (46%) of the overall studies employed a noninfectious microbial toxin challenge alone and in which the potential adverse effects of an immunosuppressive treatment on host defense and microbial clearance could not be tested. Although 19 of the studies reporting the effects of niacin or NAD metabolite treatment on survival did employ an infectious challenge, only 2 of these studies combined those treatments with the anti-microbial treatment patients would routinely receive^62,69^. An effective anti-microbial treatment could very well negate any benefit associated with a niacin or NAD metabolite type treatment. Moreover, despite the strong interest in the use of niacin or NAD metabolites raised by COVID-19, only five studies here employed a viral challenge, including two using SARS-CoV-2 models^13,42,46,50,53^).

Second, only four studies including one investigating survival, tested the effects of niacin or NAD metabolite treatment when administered more than 24h after challenge^31,38,41,54^. The survival study modeled chronic infection with *M. lepraemurium* and demonstrated a survival effect of treatment on the side of benefit^31^. However, many patients present for treatment of an acute bacterial or viral infection more than one or two days after symptoms develop that demonstrate the presence of infection^79^. Whether treatment with niacin or an NAD metabolite for acute bacterial or viral infection at these later times is beneficial has yet to be tested.

Third, despite sensitivity analyses assessing the effects of type of challenge, timing and type of niacin or NAD metabolite treatment, and study size, it was not possible to identify the source of heterogeneity underlying the effects of treatment on survival, which was the most frequent outcome reported. Fourth, based on very limited sample size estimates, initial randomization of animals and blinding of treatments and outcome measures, study quality was low. This weakness may be reflected in the significant publication bias noted across studies.

Clinically, sepsis is a syndrome without a validated criterion standard diagnostic test due to its biological and clinical heterogeneity in patients that differ in age, sex, underlying comorbidities, concurrent injuries (including surgery), medications, and infectious pathogens^80^. Human sepsis is primarily caused by gram-positive bacteria, followed by gram-negative bacteria and to a lesser extent fungi and viruses^81^. Each of these pathogens may exhibit distinct host inflammation and damage responses in the progression of sepsis^82^. Consequently, the etiological diversity in the human condition poses a complexity in recapitulating the syndrome experimentally in animal models.

Cecal ligation puncture models or pathogens administered intravenously, intratracheally or intraperitoneally may not characterize the natural evolution of sepsis^83,84^, particularly in rodents that have a natural resistance to the pathogen, possess a shorter lifespan than humans, and exhibit differences in lymphoid development, phenotypic immune markers (e.g. Fc receptors, checkpoint receptors, γ/δ T cell receptors), and the microbiome^83–86^. Despite several limitations, animal models remain essential in the development of sepsis therapies. To further our knowledge of niacin and NAD metabolites, fundamental information about their pharmacokinetics, toxicity, and mechanism of drug action alone and in the context of normal patient care (e.g. antimicrobials and adequate fluid resuscitation post-infection) need to be further studied.

Moreover, oral administration of niacin may contribute to the prevention or progression of systemic diseases affecting the heart, kidney, and nervous system^87^. However, in sepsis, oral administration is constrained by patient health, highlighting a need for continued preclinical study to identify safety, standards in dosing, and algorithms to intravenously or intraperitoneally administer niacin or NAD metabolites. Niacin is also complicated by potential flushing effects and hepatotoxicity^88^. These adverse events may be the result of aberrant metabolism of prostaglandins^89^ and cholesterol proteins^90^ by macrophages, suggesting a need to explore macrophage NAD metabolism and function in the context of infectious disease models involving niacin or NAD metabolite therapies.

Further, NAD metabolite enzymes released during sepsis (e.g. extracellular NAPRT^91^, eNAMPT^7^) may identify not only pathways disrupted but opportunities for NAD metabolite therapeutic treatments to encourage downstream production of NAD. Understanding the effects of niacin or NAD metabolites on circulating NAMPT or NAPRT, which also function as toll-like receptor 4 (TLR4) ligands^92^, may provide insight into biological, immunological, and pathological functions of cells that damage tissue and organs during severe infections.

Overall, this systematic review has identified strategies to design preclinical studies involving niacin or NAD metabolites. Studies should employ live microbial challenges that simulate the time course and pathogenesis of either the acute or chronic infections being targeted for therapy. Niacin or NAD metabolite agents should be administered in combination with the types of standard anti-microbial agents that patients would receive. If survival is an endpoint, the model should be developed to produce lethality in the presence of those anti-microbial agents. All treatments should be initiated following the onset of infection based ideally on the types of symptoms or other markers that would trigger therapy clinically. Particularly when survival is an endpoint, studies should be powered based on pilot studies that estimate the effect size of the niacin or NAD metabolite regimen being explored. Finally, studies should incorporate the randomization and blinding of treatment allocation and a priori determined outcome measures that would be typical for a randomized controlled clinical trial.

This systematic review has limitations. It only included original research journal articles published in English; therefore, studies not published in English or published as a conference abstract/paper, dissertation, or review may have been missed. We included 56 studies, many published since 2020, that provide a comprehensive picture of the present state of controlled preclinical animal investigation directed at the use of niacin and NAD metabolite treatment for infection and sepsis. Some measures in studies such as the effects of treatment on hemodynamics, apoptosis or immune cell populations were not included in analysis. But the review and analysis does include many types of data that would potentially support the effectiveness of niacin and NAD metabolite treatment. Some authors of included reports did not respond to requests for clarification of data provided in figures alone. To account for this disparity, at least two of the present report’s authors reached consensus as to the data included in analysis.

In conclusion, while there is growing interest in the possible use of niacin and NAD metabolites as immunomodulatory agents to supplement standard antimicrobial therapies for infection and sepsis, published preclinical animal studies do not yet provide a strong basis for clinical trials. Animal infection models and treatment regimens better simulating conditions that would be encountered clinically, including randomized and blinded study designs, assessment of dosing, markers of adverse events (hepatotoxicity, flushing) and disease (NAPRT, NAMPT) comparable to those employed in clinical trials are needed. These continued preclinical studies of niacin and NAD metabolites are warranted to further their utility as independent and/or adjunctive treatment strategies in infectious disease.

## Supplementary Information

Supplementary Information 1.

Supplementary Information 2.

Supplementary Information 3.

Supplementary Information 4.

Supplementary Information 5.

Supplementary Information 6.

Supplementary Information 7.

Supplementary Information 8.

Supplementary Information 9.

Supplementary Information 10.

Supplementary Information 11.

Supplementary Information 12.

Supplementary Information 13.

Supplementary Information 14.

Supplementary Information 15.

Supplementary Information 16.

Supplementary Information 17.

Supplementary Information 18.

Supplementary Information 19.

Supplementary Information 20.

Supplementary Information 21.

Supplementary Information 22.

Supplementary Information 23.

Supplementary Information 24.

Supplementary Information 25.

Supplementary Information 26.

Supplementary Information 27.

Supplementary Information 28.

Supplementary Information 29.

Supplementary Information 30.

Supplementary Information 31.

Supplementary Information 32.

Supplementary Information 33.

Supplementary Information 34.

Supplementary Information 35.

## Data Availability

All data generated or analysed during this study are included in this published article [and its supplementary information files].

## References

[1] Rudd, K. E. et al. Global, regional, and national sepsis incidence and mortality, 1990–2017: Analysis for the Global Burden of Disease Study. *Lancet* **395**(10219), 200–211 (2020). 31954465 10.1016/S0140-6736(19)32989-7 PMC6970225

[2] van den Berg, M., van Beuningen, F. E., Ter Maaten, J. C. & Bouma, H. R. Hospital-related costs of sepsis around the world: A systematic review exploring the economic burden of sepsis. *J. Crit. Care* **71**, 154096 (2022). 35839604 10.1016/j.jcrc.2022.154096

[3] Fleischmann-Struzek, C. et al. Epidemiology and costs of postsepsis morbidity, nursing care dependency, and mortality in Germany, 2013 to 2017. *JAMA Netw. Open* **4**(11), e2134290 (2021). 34767025 10.1001/jamanetworkopen.2021.34290 PMC8590172

[4] Mostel, Z. et al. Post-sepsis syndrome - an evolving entity that afflicts survivors of sepsis. *Mol. Med.* **26**(1), 6 (2019). 31892321 10.1186/s10020-019-0132-z PMC6938630

[5] Amjad, S. et al. Role of NAD(+) in regulating cellular and metabolic signaling pathways. *Mol. Metab.* **49**, 101195 (2021). 33609766 10.1016/j.molmet.2021.101195 PMC7973386

[6] Curran, C. S. et al. Nicotinamide antagonizes lipopolysaccharide-induced hypoxic cell signals in human macrophages. *J. Immunol.* **211**(2), 261–273 (2023). 37314413 10.4049/jimmunol.2200552 PMC10315438

[7] Bime, C. et al. Circulating eNAMPT as a biomarker in the critically ill: acute pancreatitis, sepsis, trauma, and acute respiratory distress syndrome. *BMC Anesthesiol.* **22**(1), 182 (2022). 35705899 10.1186/s12871-022-01718-1 PMC9198204

[8] Zeden, J. P. et al. Excessive tryptophan catabolism along the kynurenine pathway precedes ongoing sepsis in critically ill patients. *Anaesth. Intensive Care* **38**(2), 307–316 (2010). 20369765 10.1177/0310057X1003800213

[9] Lu, G. et al. Landscape of metabolic fingerprinting for diagnosis and risk stratification of sepsis. *Front. Immunol.* **13**, 883628 (2022). 35663956 10.3389/fimmu.2022.883628 PMC9159301

[10] Troche, G. et al. Tryptophan pathway catabolites (serotonin, 5-hydroxyindolacetic acid, kynurenine) and enzymes (monoamine oxidase and indole amine 2,3 dioxygenase) in patients with septic shock: A prospective observational study versus healthy controls. *Medicine (Baltimore)* **99**(19), e19906 (2020). 32384433 10.1097/MD.0000000000019906 PMC7220452

[11] He, S. et al. NAD(+) ameliorates endotoxin-induced acute kidney injury in a sirtuin1-dependent manner via GSK-3beta/Nrf2 signalling pathway. *J. Cell. Mol. Med.* **26**(7), 1979–1993 (2022). 35137552 10.1111/jcmm.17222 PMC8980955

[12] He, S. et al. Nicotinamide mononucleotide alleviates endotoxin-induced acute lung injury by modulating macrophage polarization via the SIRT1/NF-kappaB pathway. *Pharm. Biol.* **62**(1), 22–32 (2024). 38100537 10.1080/13880209.2023.2292256 PMC10732210

[13] Jiang, Y. et al. Treatment of SARS-CoV-2-induced pneumonia with NAD(+) and NMN in two mouse models. *Cell Discov.* **8**(1), 38 (2022). 35487885 10.1038/s41421-022-00409-y PMC9053567

[14] Kwon, W. Y., Suh, G. J., Kim, K. S. & Kwak, Y. H. Niacin attenuates lung inflammation and improves survival during sepsis by downregulating the nuclear factor-kappaB pathway. *Crit. Care Med.* **39**(2), 328–334 (2011). 20975550 10.1097/CCM.0b013e3181feeae4

[15] Li, H. R. et al. beta-Nicotinamide mononucleotide activates NAD+/SIRT1 pathway and attenuates inflammatory and oxidative responses in the hippocampus regions of septic mice. *Redox Biol.* **63**, 102745 (2023). 37201414 10.1016/j.redox.2023.102745 PMC10206198

[16] Nagai, A., Yasui, S., Ozawa, Y., Uno, H. & Konno, K. Niacin attenuates acute lung injury induced by lipopolysaccharide in the hamster. *Eur. Respir. J.* **7**(6), 1125–1130 (1994). 7925883

[17] Roboon, J. et al. Inhibition of CD38 and supplementation of nicotinamide riboside ameliorate lipopolysaccharide-induced microglial and astrocytic neuroinflammation by increasing NAD(). *J Neurochem* **158**(2), 311–327 (2021). 33871064 10.1111/jnc.15367 PMC8282715

[18] Murray, M. F. Nicotinamide: an oral antimicrobial agent with activity against both *Mycobacterium**tuberculosis* and human immunodeficiency virus. *Clin. Infect. Dis.* **36**(4), 453–460 (2003). 12567303 10.1086/367544

[19] Bettenworth, D. et al. Nicotinamide treatment ameliorates the course of experimental colitis mediated by enhanced neutrophil-specific antibacterial clearance. *Mol. Nutr. Food Res.* **58**(7), 1474–1490 (2014). 24764203 10.1002/mnfr.201300818

[20] Fernandes, C. A. et al. Nicotinamide enhances apoptosis of G(M)-CSF-treated neutrophils and attenuates endotoxin-induced airway inflammation in mice. *Am. J. Physiol. Lung Cell. Mol. Physiol.* **300**(3), L354-361 (2011). 21131399 10.1152/ajplung.00198.2010

[21] Hong, G. et al. Administration of nicotinamide riboside prevents oxidative stress and organ injury in sepsis. *Free Radic. Biol. Med.* **123**, 125–137 (2018). 29803807 10.1016/j.freeradbiomed.2018.05.073 PMC6236680

[22] Cros, C. et al. Nicotinamide mononucleotide administration triggers macrophages reprogramming and alleviates inflammation during sepsis induced by experimental peritonitis. *Front. Mol. Biosci.* **9**, 895028 (2022). 35832733 10.3389/fmolb.2022.895028 PMC9271973

[23] Hooijmans, C. R. et al. SYRCLE’s risk of bias tool for animal studies. *BMC Med. Res. Methodol.* **14**, 43 (2014). 24667063 10.1186/1471-2288-14-43 PMC4230647

[24] Wever, K. E., Geessink, F. J., Brouwer, M. A. E., Tillema, A. & Ritskes-Hoitinga, M. A systematic review of discomfort due to toe or ear clipping in laboratory rodents. *Lab. Anim.* **51**(6), 583–600 (2017). 28429644 10.1177/0023677217705912 PMC5700778

[25] DerSimonian, R. & Laird, N. Meta-analysis in clinical trials. *Control Clin. Trials* **7**(3), 177–188 (1986). 3802833 10.1016/0197-2456(86)90046-2

[26] Higgins, J. P. & Thompson, S. G. Quantifying heterogeneity in a meta-analysis. *Stat. Med.* **21**(11), 1539–1558 (2002). 12111919 10.1002/sim.1186

[27] R: A Language and Environment for Statistical Computing. *R Foundation for Statistical Computing, Vienna, Austria* R Core Team. https://www.R-project.org/ (2024).

[28] Balduzzi, S., Rucker, G. & Schwarzer, G. How to perform a meta-analysis with R: a practical tutorial. *Evid. Based Ment. Health* **22**(4), 153–160 (2019). 31563865 10.1136/ebmental-2019-300117 PMC10231495

[29] Abdel Rasheed, N. O., Shiha, N. A., Mohamed, S. S. & Ibrahim, W. W. SIRT1/PARP-1/NLRP3 cascade as a potential target for niacin neuroprotective effect in lipopolysaccharide-induced depressive-like behavior in mice. *Int. Immunopharmacol.* **123**, 110720 (2023). 37562290 10.1016/j.intimp.2023.110720

[30] Cao, T. et al. Nicotinamide mononucleotide as a therapeutic agent to alleviate multi-organ failure in sepsis. *J. Transl. Med.* **21**(1), 883 (2023). 38057866 10.1186/s12967-023-04767-3 PMC10699070

[31] Chang, Y. T. Chemotherapy of murine leprosy. III. The effects of nicotinamide and pyrazinamide (aldinamide) on mouse leprosy. *Int. J. Lepr.* **22**(3), 331–346 (1954). 13232783

[32] Doganay, S. B. et al. Antioxidant and anti-inflammatory effects of nicotinamide adenine dinucleotide (NAD+) against acute hepatorenal oxidative injury in an experimental sepsis model. *Kafkas Univ. Vet Fak Derg* **28**(1), 121–130 (2022).

[33] Du, S. H. et al. Nicotinamide mononucleotide ameliorates acute lung injury by inducing mitonuclear protein imbalance and activating the UPR(mt). *Exp. Biol. Med. (Maywood)* **247**(14), 1264–1276 (2022). 35538652 10.1177/15353702221094235 PMC9379602

[34] Duan, S., Kim, S. G., Lim, H. J., Song, H. R. & Han, M. K. Interferon-beta alleviates sepsis by SIRT1-mediated blockage of endothelial glycocalyx shedding. *BMB Rep.* **56**(5), 314–319 (2023). 37013347 10.5483/BMBRep.2023-0030 PMC10230016

[35] Fukuzawa, M. et al. Inhibitory effect of nicotinamide on in vitro and in vivo production of tumor necrosis factor-alpha. *Immunol. Lett.* **59**(1), 7–11 (1997). 9334851 10.1016/s0165-2478(97)00088-6

[36] Fulton, R. L. Prevention of endotoxic death with nicotinamide and adenosine triphosphate. *Surg. Forum* **25**, 17–19 (1974). 4280146

[37] Greisman, S. E., DuBuy, J. B. & Woodward, C. L. Experimental gram-negative bacterial sepsis: Prevention of mortality not preventable by antibiotics alone. *Infect. Immun.* **25**(2), 538–557 (1979). 385500 10.1128/iai.25.2.538-557.1979 PMC443579

[38] Guo, W. et al. Niacin alleviates dairy cow mastitis by regulating the GPR109A/AMPK/NRF2 signaling pathway. *Int. J. Mol. Sci.* **21**(9), 3321 (2020). 32397071 10.3390/ijms21093321 PMC7246865

[39] Guo, W. et al. GPR109A alleviate mastitis and enhances the blood milk barrier by activating AMPK/Nrf2 and autophagy. *Int. J. Biol. Sci.* **17**(15), 4271–4284 (2021). 34803497 10.7150/ijbs.62380 PMC8579459

[40] Han, X. et al. NAD+ ameliorates inflammation-induced epithelial barrier dysfunction in cultured enterocytes and mouse ileal mucosa. *J. Pharmacol. Exp. Ther.* **307**(2), 443–449 (2003). 12975482 10.1124/jpet.103.056556

[41] He, D. et al. Activation of HCA2 regulates microglial responses to alleviate neurodegeneration in LPS-induced in vivo and in vitro models. *J. Neuroinflamm.* **20**(1), 86 (2023). 10.1186/s12974-023-02762-5 PMC10053461 36991440

[42] He, M. et al. SIRT1-mediated downregulation of p27Kip1 is essential for overcoming contact inhibition of Kaposi’s sarcoma-associated herpesvirus transformed cells. *Oncotarget* **7**(46), 75698–75711 (2016). 27708228 10.18632/oncotarget.12359 PMC5342771

[43] Hilton, J. G. & Wells, C. H. Effects of indomethacin and nicotinic acid on *E*. *coli* endotoxin shock in anesthetized dogs. *J Trauma* **16**(12), 968–973 (1976). 794510 10.1097/00005373-197612000-00005

[44] Imaruoka, K. et al. Nicotinamide alleviates kidney injury and pregnancy outcomes in lupus-prone MRL/lpr mice treated with lipopolysaccharide. *Biochem. Biophys. Res. Commun.* **510**(4), 587–593 (2019). 30739788 10.1016/j.bbrc.2019.01.110

[45] Iske, J. et al. NAD(+) prevents septic shock-induced death by non-canonical inflammasome blockade and IL-10 cytokine production in macrophages. *Elife*10.7554/eLife.88686 (2024). 38372712 10.7554/eLife.88686 PMC10942599

[46] Izadpanah, A. et al. SARS-CoV-2 infection dysregulates NAD metabolism. *Front. Immunol.* **14**, 1158455 (2023). 37457744 10.3389/fimmu.2023.1158455 PMC10344451

[47] Kao, S. J., Liu, D. D., Su, C. F. & Chen, H. I. Niacinamide abrogates the organ dysfunction and acute lung injury caused by endotoxin. *J. Cardiovasc. Pharmacol.* **50**(3), 333–342 (2007). 17878764 10.1097/FJC.0b013e3180cbd18a

[48] Kwon, W. Y. et al. Niacin and selenium attenuate sepsis-induced lung injury by up-regulating nuclear factor erythroid 2-related factor 2 signaling. *Crit. Care Med.* **44**(6), e370-382 (2016). 26646455 10.1097/CCM.0000000000001422

[49] LeClaire, R. D., Kell, W., Bavari, S., Smith, T. J. & Hunt, R. E. Protective effects of niacinamide in staphylococcal enterotoxin-B-induced toxicity. *Toxicology* **107**(1), 69–81 (1996). 8597033 10.1016/0300-483x(95)03202-q

[50] Li, W. Y. et al. The SIRT1 inhibitor, nicotinamide, inhibits hepatitis B virus replication in vitro and in vivo. *Arch. Virol.* **161**(3), 621–630 (2016). 26660162 10.1007/s00705-015-2712-8

[51] Liu, J. et al. Supplementation of nicotinamide mononucleotide diminishes COX-2 associated inflammatory responses in macrophages by activating kynurenine/AhR signaling. *Free Radic. Biol. Med.* **214**, 69–79 (2024). 38336100 10.1016/j.freeradbiomed.2024.01.046

[52] Micheva-Viteva, S. N. et al. Increased mortality in mice following immunoprophylaxis therapy with high dosage of nicotinamide in burkholderia persistent infections. *Infect. Immun.*10.1128/IAI.00592-18 (2019). 30323029 10.1128/IAI.00592-18 PMC6300628

[53] Mo, Y. et al. Nicotinamide mononucleotide impacts HIV-1 infection by modulating immune activation in T lymphocytes and humanized mice. *EBioMedicine* **98**, 104877 (2023). 37980794 10.1016/j.ebiom.2023.104877 PMC10694053

[54] Pacl, H. T. et al. NAD(H) homeostasis underlies host protection mediated by glycolytic myeloid cells in tuberculosis. *Nat. Commun.* **14**(1), 5472 (2023). 37673914 10.1038/s41467-023-40545-x PMC10482943

[55] Park, H. et al. Combination therapy of niacin and apocynin attenuates lung injury during sepsis in rats. *J. Surg. Res.* **285**, 51–58 (2023). 36640610 10.1016/j.jss.2022.12.020

[56] Pulido, E. J. et al. Inhibition of PARS attenuates endotoxin-induced dysfunction of pulmonary vasorelaxation. *Am. J. Physiol.* **277**(4), L769-776 (1999). 10516218 10.1152/ajplung.1999.277.4.L769

[57] Rodriguez Cetina Biefer, H. et al. Mast cells regulate CD4(+) T-cell differentiation in the absence of antigen presentation. *J. Allergy Clin. Immunol.* **142**(6), 1894-1908e1897 (2018). 29470999 10.1016/j.jaci.2018.01.038 PMC6454881

[58] Scharte, M. et al. Nicotinamide increases systemic vascular resistance in ovine endotoxemia. *Intensive Care Med.* **29**(6), 989–994 (2003). 12728305 10.1007/s00134-003-1738-7

[59] Selli, J., Vural Keles, D., Keles, O. N., Celik, M. & Yetim, Z. Nicotinamide riboside preserves ovarian injury in experimental sepsis model in rats. *Eurasian J. Med.* **55**(2), 128–134 (2023). 36648023 10.5152/eurasianjmed.2023.22255 PMC10440975

[60] Shaw, R. C. et al. A bioassay of treatment of hemorrhagic shock. 3. Effects of a saline solution, ascorbic acid, and nicotinamide upon the toxicity of endotoxin for rats. *Arch. Surg.* **93**(4), 562–566 (1966). 4224203 10.1001/archsurg.1966.01330040026003

[61] Shi, Y. et al. Activated niacin receptor HCA2 inhibits chemoattractant-mediated macrophage migration via Gbetagamma/PKC/ERK1/2 pathway and heterologous receptor desensitization. *Sci. Rep.* **7**, 42279 (2017). 28186140 10.1038/srep42279 PMC5301212

[62] Smith, I. M. & Burmeister, L. F. Biochemically assisted antibiotic treatment of lethal murine Staphylococcus aureus septic shock. *Am. J. Clin. Nutr.* **30**(8), 1364–1368 (1977). 142426 10.1093/ajcn/30.8.1364

[63] Tian, Y. et al. Nicotinamide mononucleotide attenuates LPS-induced acute lung injury with anti-inflammatory, anti-oxidative and anti-apoptotic effects. *J. Surg. Res.* **283**, 9–18 (2023). 36347171 10.1016/j.jss.2022.09.030

[64] Umapathy, N. S. et al. beta-Nicotinamide adenine dinucleotide attenuates lipopolysaccharide-induced inflammatory effects in a murine model of acute lung injury. *Exp. Lung Res.* **38**(5), 223–232 (2012). 22563684 10.3109/01902148.2012.673049 PMC3678723

[65] Wray, G. M., Hinds, C. J. & Thiemermann, C. Effects of inhibitors of poly(ADP-ribose) synthetase activity on hypotension and multiple organ dysfunction caused by endotoxin. *Shock* **10**(1), 13–19 (1998). 9688085 10.1097/00024382-199807000-00003

[66] Wurtele, H. et al. Modulation of histone H3 lysine 56 acetylation as an antifungal therapeutic strategy. *Nat. Med.* **16**(7), 774–780 (2010). 20601951 10.1038/nm.2175 PMC4108442

[67] Xing, X. et al. Effect of nicotinamide against *Candida**albicans*. *Front. Microbiol.* **10**, 595 (2019). 30972047 10.3389/fmicb.2019.00595 PMC6443637

[68] Xu, W. et al. Novel role of resveratrol: suppression of high-mobility group protein box 1 nucleocytoplasmic translocation by the upregulation of sirtuin 1 in sepsis-induced liver injury. *Shock* **42**(5), 440–447 (2014). 25004063 10.1097/SHK.0000000000000225

[69] Yan, Y., Liao, Z., Shen, J., Zhu, Z. & Cao, Y. Nicotinamide potentiates amphotericin B activity against Candida albicans. *Virulence* **13**(1), 1533–1542 (2022). 36068709 10.1080/21505594.2022.2119656 PMC9467617

[70] Ye, M. et al. NAD(H)-loaded nanoparticles for efficient sepsis therapy via modulating immune and vascular homeostasis. *Nat. Nanotechnol.* **17**(8), 880–890 (2022). 35668170 10.1038/s41565-022-01137-w PMC10044491

[71] Yuan, H. et al. Therapeutic benefits of the group B3 vitamin nicotinamide in mice with lethal endotoxemia and polymicrobial sepsis. *Pharmacol. Res.* **65**(3), 328–337 (2012). 22154801 10.1016/j.phrs.2011.11.014

[72] Zhao, G. J. et al. Supplementation with nicotinamide riboside attenuates T cell exhaustion and improves survival in sepsis. *Shock* **60**(2), 238–247 (2023). 37314209 10.1097/SHK.0000000000002153 PMC10476598

[73] Zingarelli, B., Salzman, A. L. & Szabo, C. Protective effects of nicotinamide against nitric oxide-mediated delayed vascular failure in endotoxic shock: Potential involvement of polyADP ribosyl synthetase. *Shock* **5**(4), 258–264 (1996). 8721385 10.1097/00024382-199604000-00005

[74] Singhal, A. & Cheng, C. Y. Host NAD+ metabolism and infections: Therapeutic implications. *Int. Immunol.* **31**(2), 59–67 (2019). 30329059 10.1093/intimm/dxy068

[75] Suchard, M. S. & Savulescu, D. M. Nicotinamide pathways as the root cause of sepsis - an evolutionary perspective on macrophage energetic shifts. *FEBS J.* **289**(4), 955–964 (2022). 33686748 10.1111/febs.15807 PMC9545938

[76] Brenner, C. Viral infection as an NAD(+) battlefield. *Nat. Metab.* **4**(1), 2–3 (2022). 34980922 10.1038/s42255-021-00507-3 PMC10155260

[77] Guo, X. et al. NAD + salvage governs mitochondrial metabolism, invigorating natural killer cell antitumor immunity. *Hepatology* **78**(2), 468–485 (2023). 35815363 10.1002/hep.32658

[78] Raines, N. H. et al. Niacinamide may be associated with improved outcomes in COVID-19-related acute kidney injury: An observational study. *Kidney* **2**(1), 33–41 (2021). 10.34067/KID.0006452020 PMC8785722 35368823

[79] Escadafal, C. et al. Bacterial versus non-bacterial infections: A methodology to support use-case-driven product development of diagnostics. *BMJ Glob. Health* **5**(10), e003141 (2020). 33087393 10.1136/bmjgh-2020-003141 PMC7580043

[80] Singer, M. et al. The Third International Consensus definitions for sepsis and septic shock (Sepsis-3). *JAMA* **315**(8), 801–810 (2016). 26903338 10.1001/jama.2016.0287 PMC4968574

[81] van Vught, L. A. et al. Incidence, risk factors, and attributable mortality of secondary infections in the intensive care unit after admission for sepsis. *JAMA* **315**(14), 1469–1479 (2016). 26975785 10.1001/jama.2016.2691

[82] Grondman, I., Pirvu, A., Riza, A., Ioana, M. & Netea, M. G. Biomarkers of inflammation and the etiology of sepsis. *Biochem. Soc. Trans.* **48**(1), 1–14 (2020). 32049312 10.1042/BST20190029

[83] Poli-de-Figueiredo, L. F., Garrido, A. G., Nakagawa, N. & Sannomiya, P. Experimental models of sepsis and their clinical relevance. *Shock* **30**(Suppl 1), 53–59 (2008). 18704008 10.1097/SHK.0b013e318181a343

[84] Lewis, A. J., Seymour, C. W. & Rosengart, M. R. Current murine models of sepsis. *Surg. Infect. (Larchmt)* **17**(4), 385–393 (2016). 27305321 10.1089/sur.2016.021 PMC4960474

[85] Mestas, J. & Hughes, C. C. Of mice and not men: differences between mouse and human immunology. *J. Immunol.* **172**(5), 2731–2738 (2004). 14978070 10.4049/jimmunol.172.5.2731

[86] Yuan, Y. et al. Early intestinal microbiota changes in aged and adult mice with sepsis. *Front. Cell. Infect. Microbiol.* **12**, 1061444 (2022). 36636721 10.3389/fcimb.2022.1061444 PMC9831679

[87] Djadjo, S. & Bajaj, T. *Niacin* (StatPearls, 2025). 31082080

[88] Bhardwaj, S. S. & Chalasani, N. Lipid-lowering agents that cause drug-induced hepatotoxicity. *Clin. Liver Dis.* **11**(3), 597–613 (2007). 17723922 10.1016/j.cld.2007.06.010 PMC2048990

[89] Meyers, C. D., Liu, P., Kamanna, V. S. & Kashyap, M. L. Nicotinic acid induces secretion of prostaglandin D2 in human macrophages: An in vitro model of the niacin flush. *Atherosclerosis* **192**(2), 253–258 (2007). 16945375 10.1016/j.atherosclerosis.2006.07.014

[90] Li, Z. et al. Niacin reduces plasma CETP levels by diminishing liver macrophage content in CETP transgenic mice. *Biochem. Pharmacol.* **84**(6), 821–829 (2012). 22750059 10.1016/j.bcp.2012.06.020

[91] Manago, A. et al. Extracellular nicotinate phosphoribosyltransferase binds Toll like receptor 4 and mediates inflammation. *Nat. Commun.* **10**(1), 4116 (2019). 31511522 10.1038/s41467-019-12055-2 PMC6739309

[92] Audrito, V., Messana, V. G. & Deaglio, S. NAMPT and NAPRT: two metabolic enzymes with key roles in inflammation. *Front. Oncol.* **10**, 358 (2020). 32266141 10.3389/fonc.2020.00358 PMC7096376

