# Supplementary material for: Meta-analysis of niacin and NAD metabolite treatment in infectious disease animal studies suggests benefit but requires confirmation in clinically relevant models
Source: Sci Rep. 2025 Apr 12;15:12621. doi: 10.1038/s41598-025-95735-y (PMC11993703; doi:10.1038/s41598-025-95735-y)
Supplement: Supplementary file 1 — Supplementary Information 1. [file 41598_2025_95735_MOESM1_ESM.docx]

**Supplemental Figure Legends**

**Supplemental Figure 1**. PRISMA Flow Diagram

**Supplemental Figures 2 to 13**. These figures show the by-study analysis determining whether results of experiments in reports in which more than one treatment group was compared to a common control could or could not be combined in analysis (p≥0.05 or p<0.05 for the differences between groups respectively, see methods) and whether the results of more than one experiment in a study could or could not be combined in analysis (p≥0.05 or p<0.05 for the differences between experiments respectively (see methods). Studies with survival are shown in **Supplemental Figure-2** (**SupFigure-2**), NAD metabolites in **SupFigure-3**, microbe levels in **SupFigure-4**, histology, permeability and chemistry organ injury measures in **SupFigures-5, -6 and -7**, tumor necrosis factor-α (TNFα), interleukin-6 (IL_6) and interleukin-1β (IL-1β) in **SupFigures-8, -9, and -10,** anti-oxidant and oxidation markers in **SupFigures-11 and -12**, and myeloperoxidase (MPO) levels in **SupFigure-13**. See **SupTables-3, -4, -7, -11 and -12** for NAD metabolite, microbe, chemistry, antioxidant and oxidation marker types included in studies respectively.

**Supplemental Figure-14**. Effects of niacin or nicotinamide adenine dinucleotide (NAD) metabolite treatment versus control on the odds ratio of mortality (OR, 95%CIs) in studies [author (y), reference] conducted in either bacterial, microbial toxin (LPS), virus or fungal challenge type models. Also shown are the numbers of animals dying and total numbers of animals in the treatment and control groups. In two studies (Cao and Rodriguez), results of two experiments could not be combined. In Nagai, there were no deaths in either treatment and control groups and no OR could be calculated. Across the 34 studies or individual experiments with analyzable data, niacin or NAD metabolite treatment was associated with an OR on the side of benefit in 27 (79% of analyzable studies), and in 11 of these, the effects were significant (i.e. the 95%CI was entirely on the side of benefit for treatment). The effects of treatment did not differ significantly comparing the challenge types studied (p=0.13). However, while treatment decreased the overall OR of mortality both within and across challenges, heterogeneity of effects due primarily to quantitative differences was significant (I^2^≥60%, p<0.01) except across studies with virus or fungal type challenges (I^2^=0%, p≥0.68). See **SupTable-1** for specific types of microbial challenges employed in studies.

**Supplemental Figure-15**. Effects of niacin or nicotinamide adenine dinucleotide (NAD) metabolite treatment versus control on the odds ratio of mortality (OR, 95%CIs) in studies [author (y), reference] conducted with treatment administered either >1d before, within the 24h before or after, and >1d after challenge. Also noted are the numbers of animals dying and total numbers of animals in the treatment and control groups. In two studies (Cao and Rodriguez), results of two experiments could not be combined. In Nagai, there were no deaths in either treatment and control groups and no OR could be calculated. Across the 31 studies or individual experiments with analyzable data, niacin or NAD metabolite treatment was associated with an OR on the side of benefit in 25 (81% of analyzable studies), and in 10 of these, the effects were significant (i.e. the 95%CI was entirely on the side of benefit for treatment). The effects of treatment did not differ significantly comparing the treatment times studied (p=0.91). However, while treatment decreased the overall OR of mortality both within treatment times and across all studies, heterogeneity of effects due primarily to quantitative differences was significant (I^2^≥68%, p<0.01) except across studies with treatment >24h after challenge (I^2^=0%, p=0.84). See **SupTable-1** for specific times of treatment.

**Supplemental Figure-16**. Effects of niacin or nicotinamide adenine dinucleotide (NAD) metabolite treatment versus control on the odds ratio of mortality (OR, 95%CIs) in studies [author (y), reference] conducted with either a salvage or Preiss-Handler type treatment. Also noted are the numbers of animals dying and total numbers of animals in the treatment and control groups. In two studies (Cao and Rodriguez), results of two experiments could not be combined. In Nagai, there were no deaths in either treatment and control groups and no OR could be calculated. Across the 31 studies or individual experiments with analyzable data, niacin or NAD metabolite treatment was associated with an OR on the side of benefit in 25 (81% of analyzable studies), and in 10 of these, the effects were significant (i.e. the 95%CI was entirely on the side of benefit for treatment). While treatment decreased the overall OR of mortality both across all studies, heterogeneity of effects due primarily to quantitative differences was significant (I^2^≥58%, p<0.01). See **SupTable-1** for specific type of treatment employed in studies.

**Supplemental Figure-17**. Effects of niacin or nicotinamide adenine dinucleotide (NAD) metabolite treatment (TMT) versus control on the standardized mean difference (95%CIs) (SMD) in chemistry injury (**Panel A**), interleukin-1β (IL-1β) (**Panel B**) and myeloperoxidase (MPO) (**Panel C**) measures in studies (author, year of publication) conducted in either mouse, rat, cow or sheep models. Animal numbers (n) for study groups are shown. Data from individual experiments that were pooled within studies based on nonsignificant heterogeneity (p≥0.05) comparing the experiments are shown in **Supplemental Figures-7, -10 and -13**. Twelve, 9 and 9 studies reported the effects of treatment on chemistry, IL-1β and MPO measures respectively, and for studies with more than one experiment, results could be combined for MPO measures but not for 2 studies of chemistry injury measures and 1 for IL-1β measure. Treatment had effects on the side of decreasing SMDs for chemistry, IL-1β and MPO measures in 15, 13 and 9 studies respectively and these decreases were significant in 10, 12, and 6 respectively (i.e. the 95%CI was entirely on the side of decreases with treatment). Effects did not differ significantly across species for IL-1β and MPO measures (p≥0.08) but did for chemistry injury measures (p<0.01). Although primarily quantitative, there was significant heterogeneity across studies within species as well as overall for both chemistry injury and IL-1β measures (I^2^≥72%, p<0.01). There was significant heterogeneity across studies for MPO measures in rats (I^2^≥71%, p<0.03)) but not for mouse or overall MPO measures (I^2^≤30%, p≥0.55).

**Supplemental Figure-18**. Effects of niacin or nicotinamide adenine dinucleotide (NAD) metabolite treatment versus control on the odds ratio of mortality (95%CIs) (OR) in studies [author (y), reference] stratified based on quartile of study size. Shown are the numbers of animals dying and total numbers of animals in the treatment and control groups. In two studies (Cao and Rodriguez), results of two experiments could not be combined. In Nagai, there were no deaths in either treatment and control groups and no OR could be calculated. Across the 31 studies or individual experiments with analyzable data, niacin or NAD metabolite treatment was associated with an OR on the side of benefit in 25 (81% of analyzable studies), and in 10 of these, the effects were significant (i.e. the 95%CI was entirely on the side of benefit for treatment). The effects of treatment did not differ significantly comparing the four quartiles (p=0.10). However, while treatment decreased the overall OR of mortality both within each quartile and across all studies, heterogeneity of effects due primarily to quantitative differences was significant (I^2^≥50%, p≤0.04) except across the second quartile of studies (I^2^=0%, p=0.99).
