# Supplementary figures and images for: Meta-analysis of niacin and NAD metabolite treatment in infectious disease animal studies suggests benefit but requires confirmation in clinically relevant models
Source: Sci Rep. 2025 Apr 12;15:12621. doi: 10.1038/s41598-025-95735-y (PMC11993703; doi:10.1038/s41598-025-95735-y)

Supplemental Figure-1: PRISMA Flow Diagram

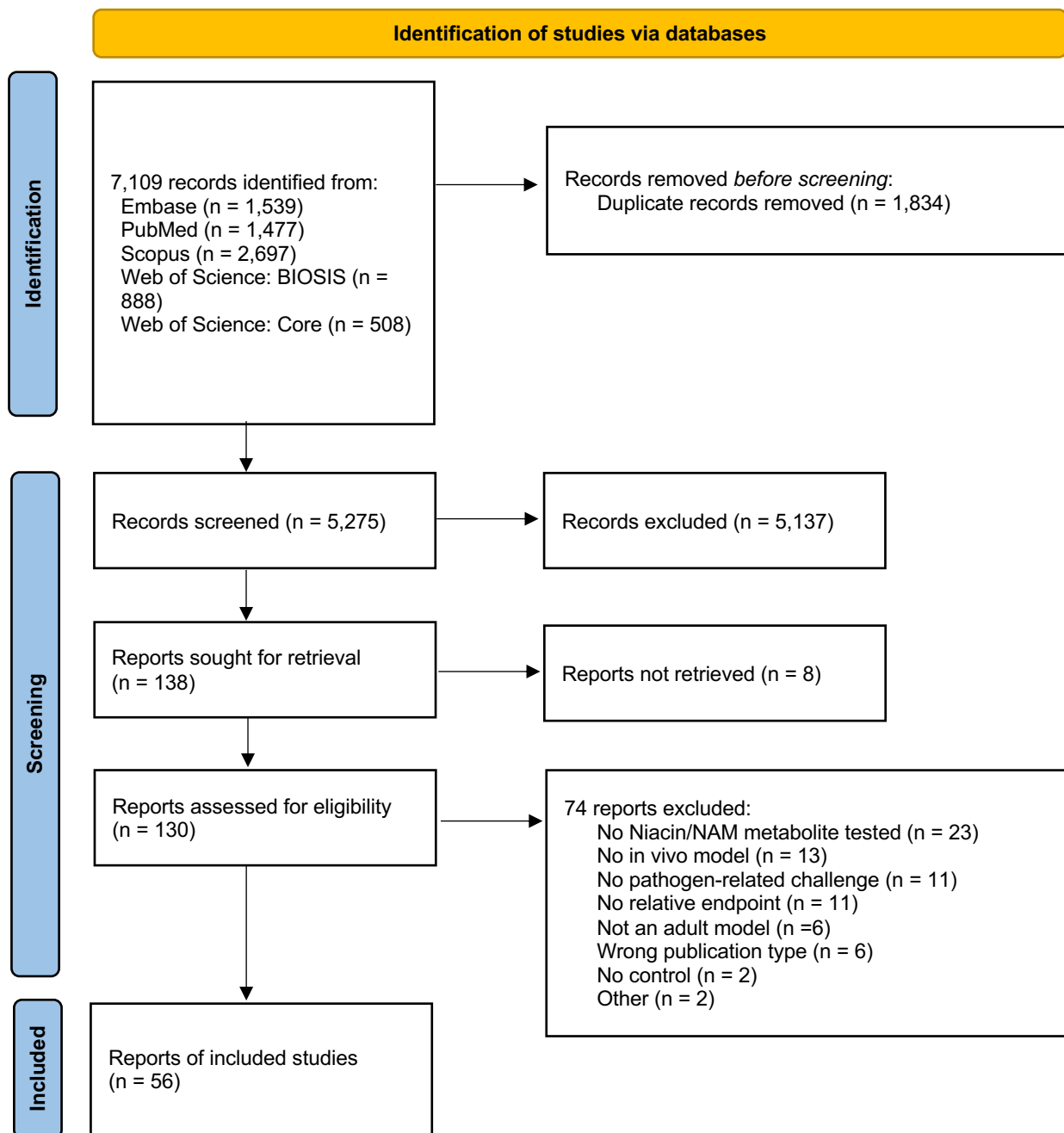

Supplement: Supplementary file 2 — Supplementary Information 2. [file 41598_2025_95735_MOESM2_ESM.pdf]

SupFigure-2. Mortality by-study analysis

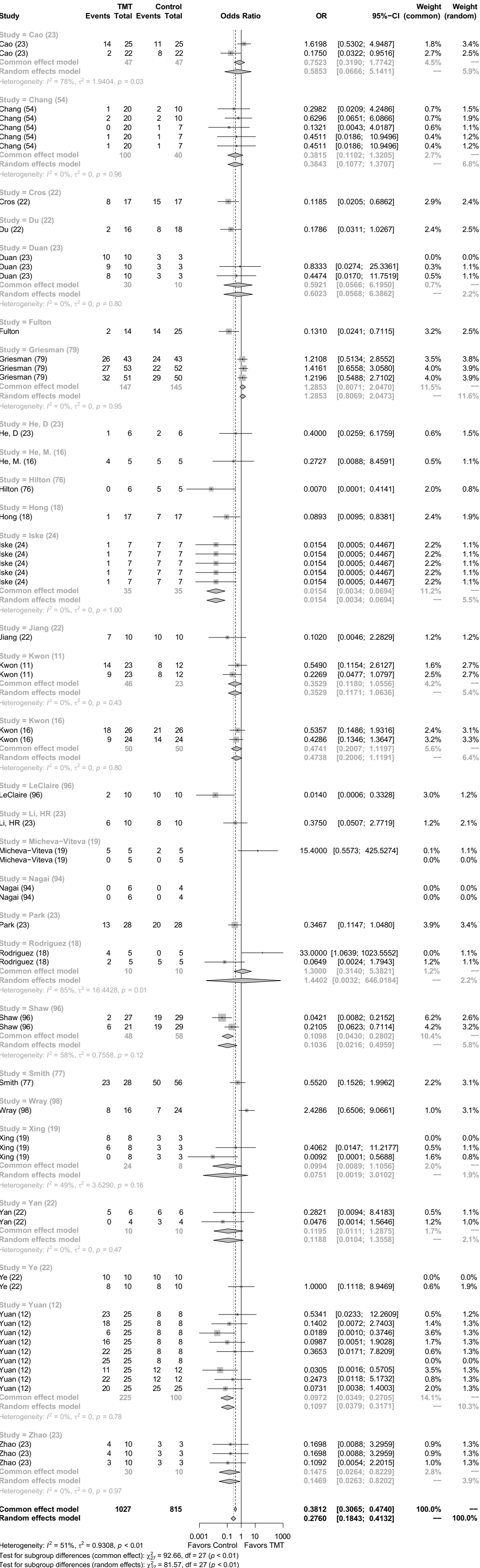

Supplement: Supplementary file 3 — Supplementary Information 3. [file 41598_2025_95735_MOESM3_ESM.pdf]

Supfigure-5. Histologic organ injury by-study analysis

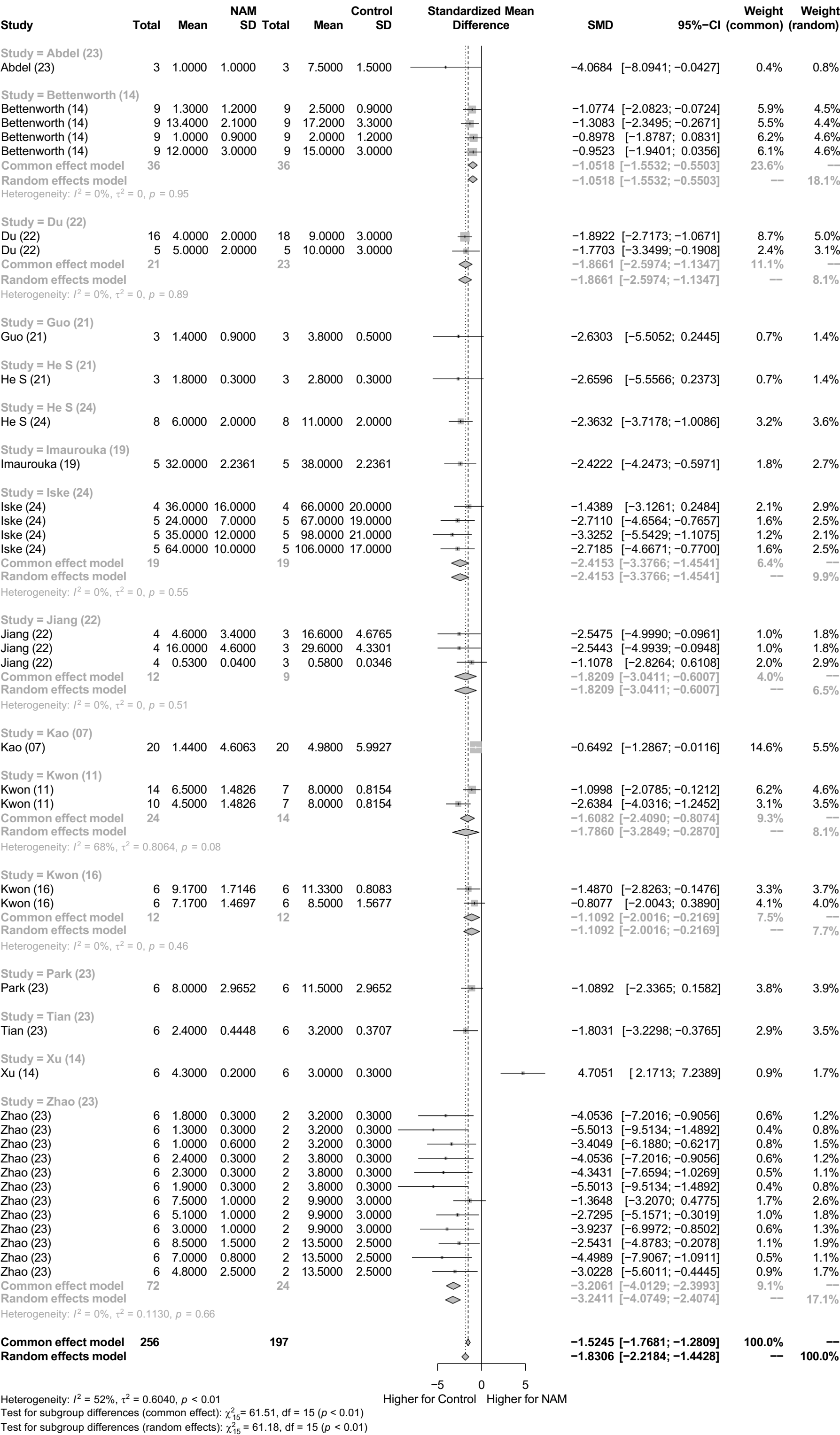

Supplement: Supplementary file 6 — Supplementary Information 6. [file 41598_2025_95735_MOESM6_ESM.pdf]

SupFigure-6. Permeability organ injury by-study analysis

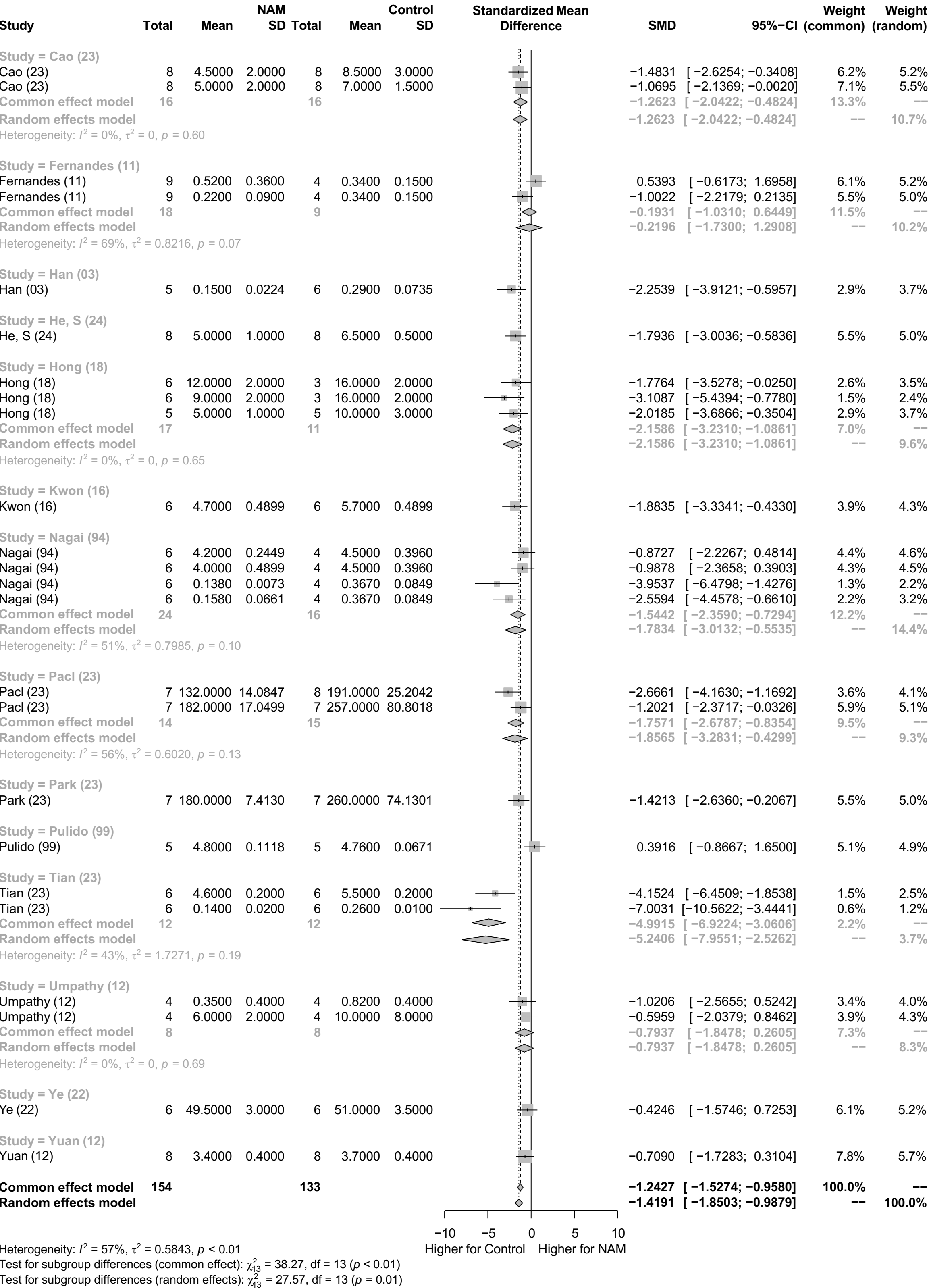

Supplement: Supplementary file 7 — Supplementary Information 7. [file 41598_2025_95735_MOESM7_ESM.pdf]

SupFigure-7. Chemistry organ injury by-study analysis

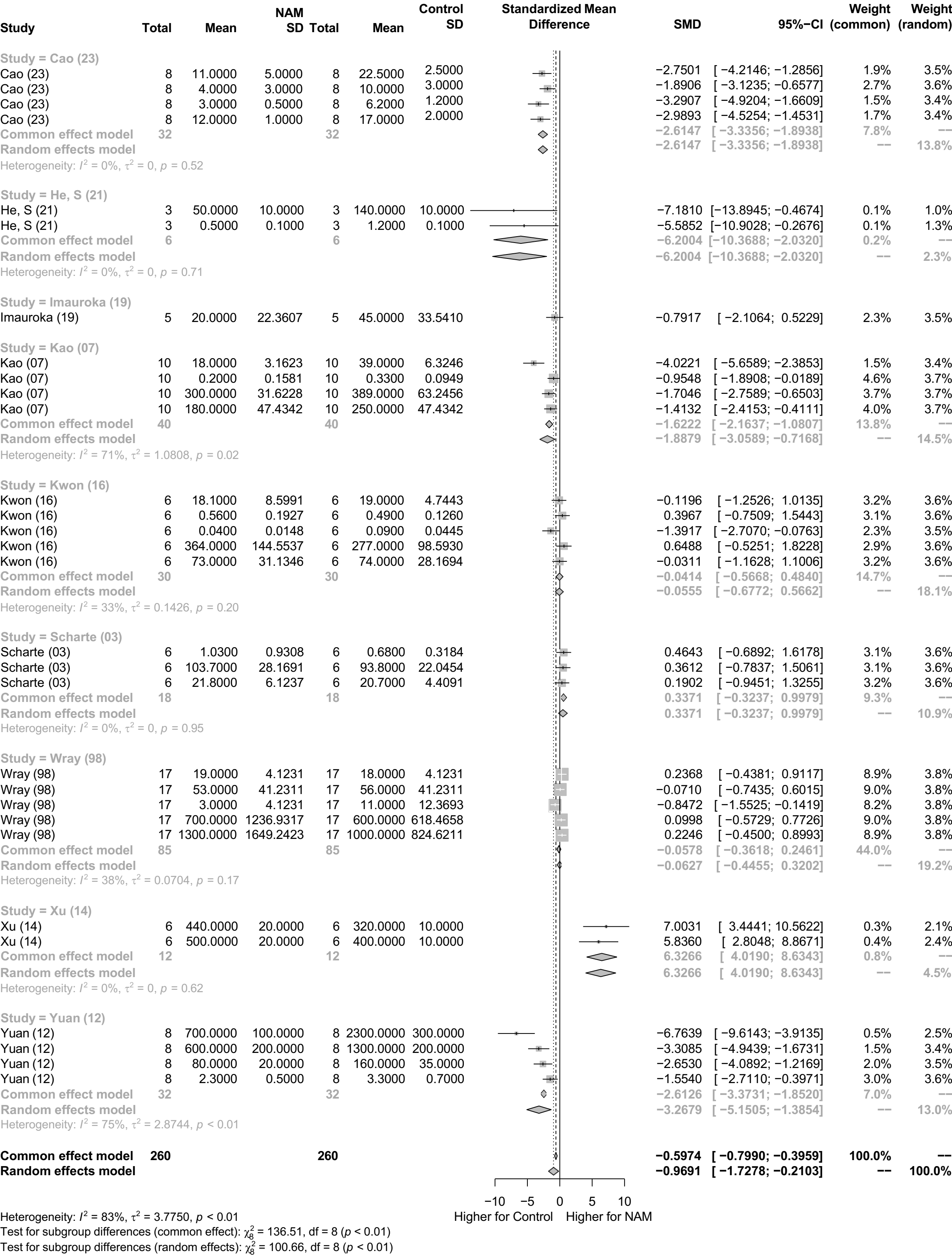

Supplement: Supplementary file 8 — Supplementary Information 8. [file 41598_2025_95735_MOESM8_ESM.pdf]

SupFigure-8. TNF-α by-study analysis

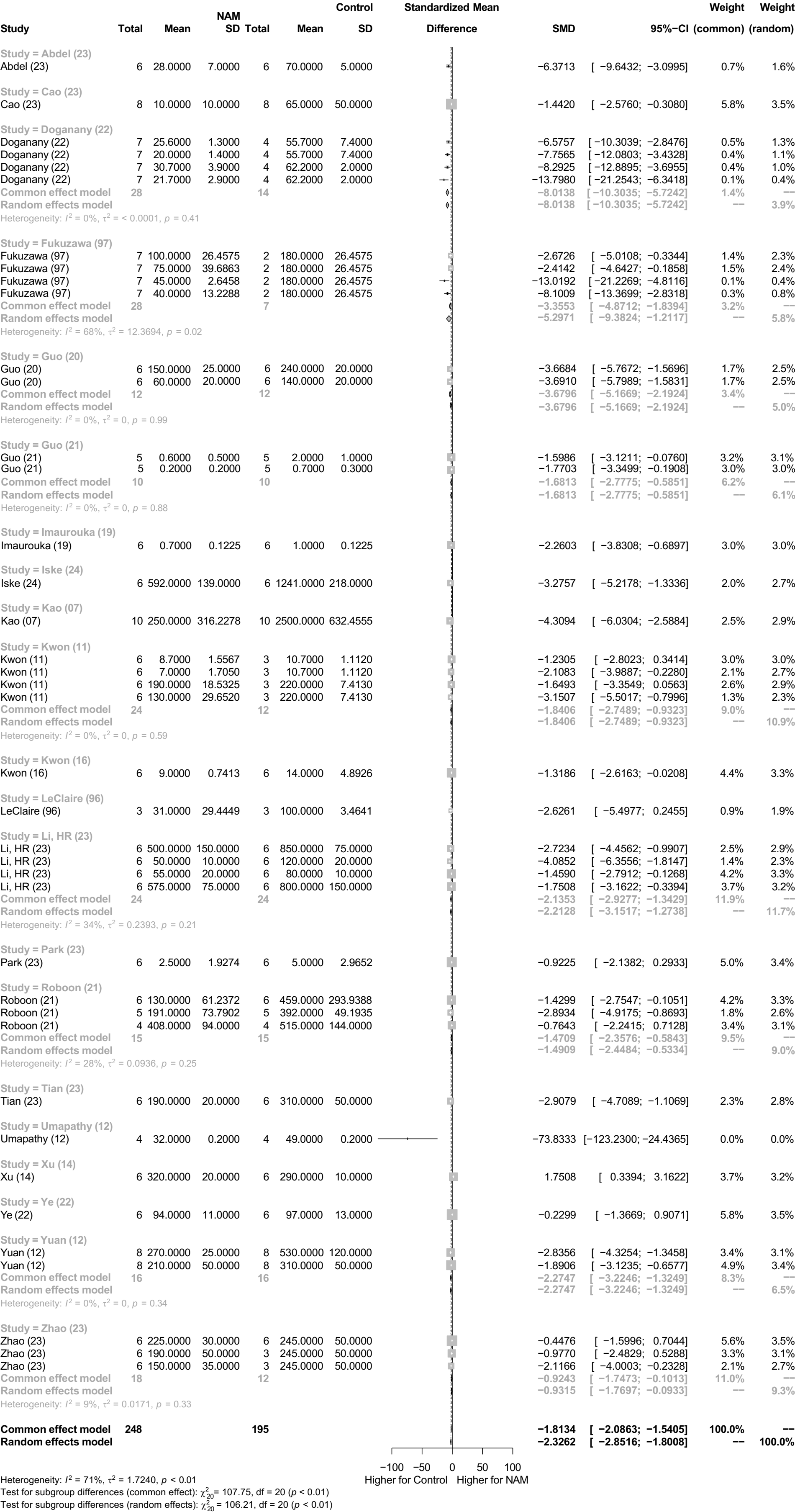

Supplement: Supplementary file 9 — Supplementary Information 9. [file 41598_2025_95735_MOESM9_ESM.pdf]

SupFigure-9. IL-6 by-study analysis

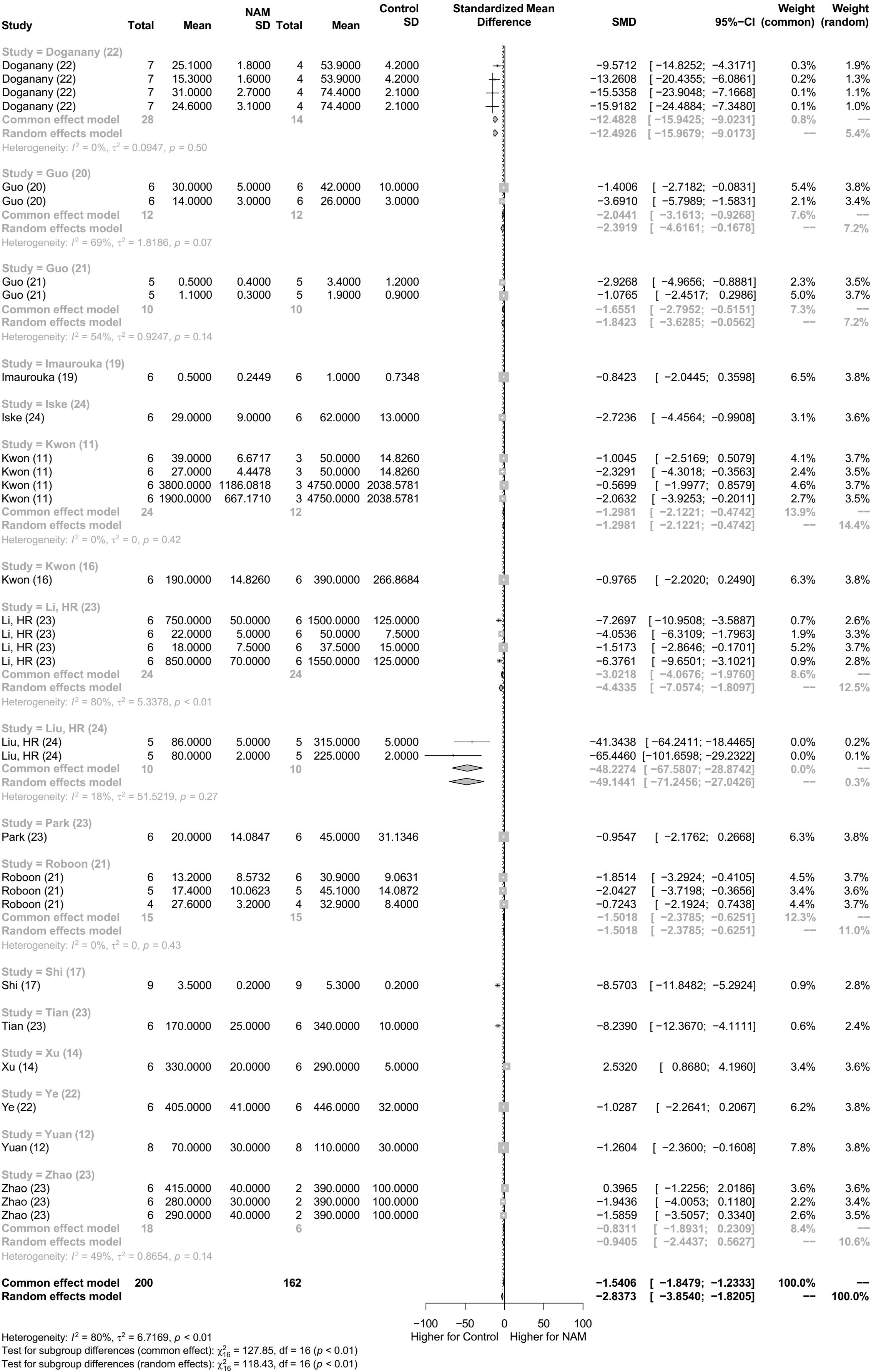

Supplement: Supplementary file 10 — Supplementary Information 10. [file 41598_2025_95735_MOESM10_ESM.pdf]

SupFigure-11. Antioxidant by-study analysis

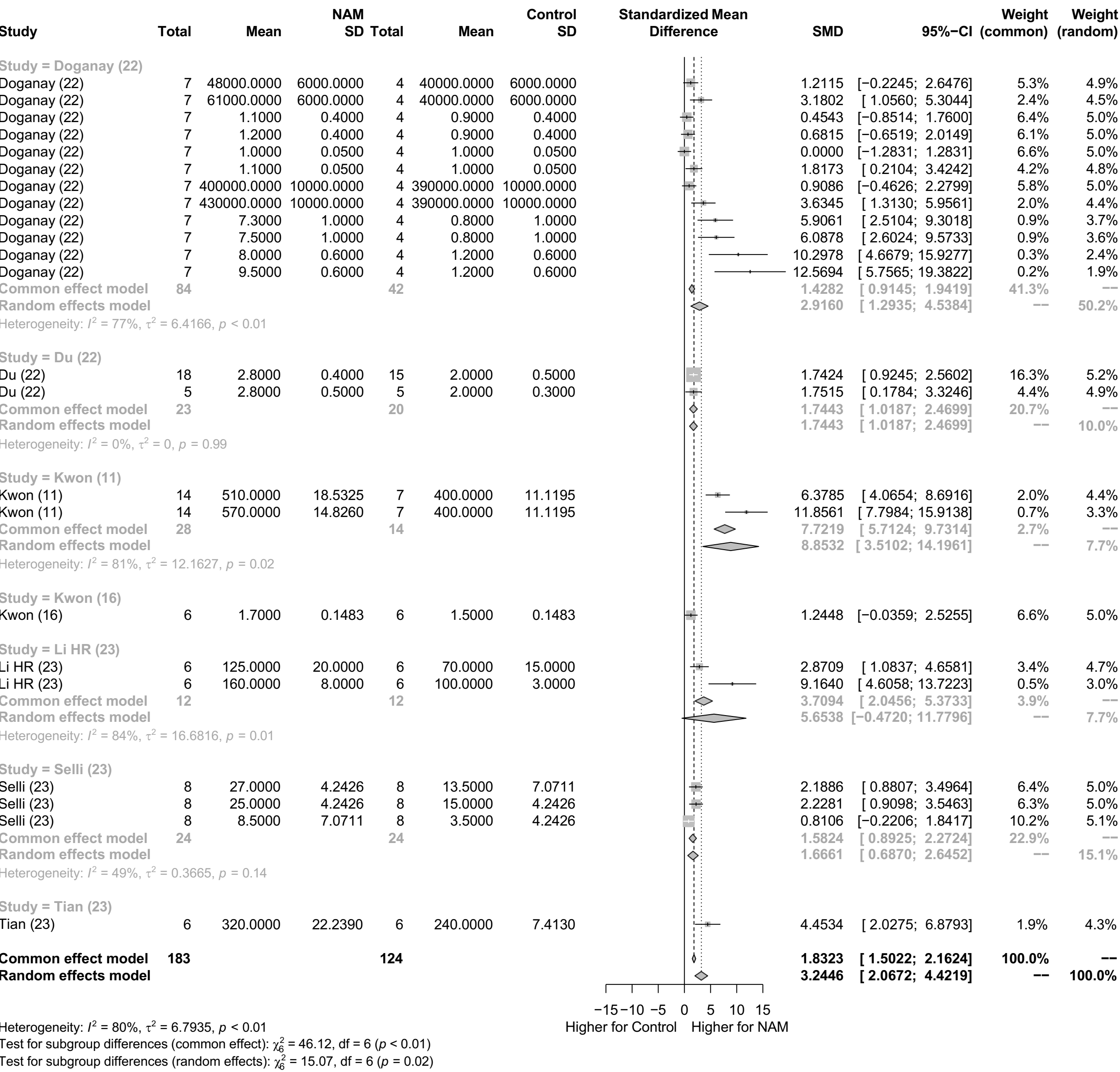

Supplement: Supplementary file 12 — Supplementary Information 12. [file 41598_2025_95735_MOESM12_ESM.pdf]

SupFigure-12. Oxidation marker by-study analysis

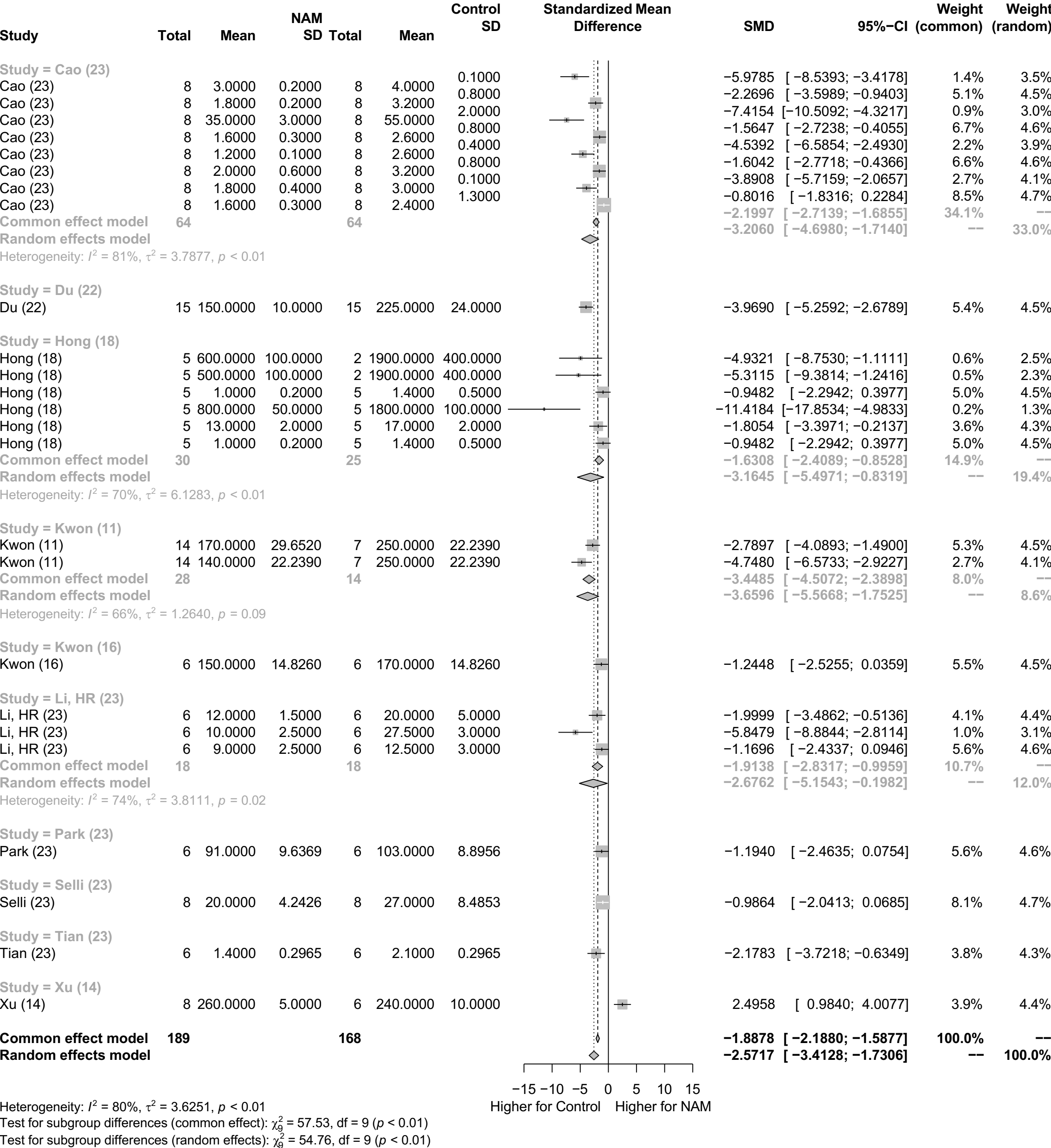

Supplement: Supplementary file 13 — Supplementary Information 13. [file 41598_2025_95735_MOESM13_ESM.pdf]

SupFigure-13. Myeloperoxidase by-study analysis

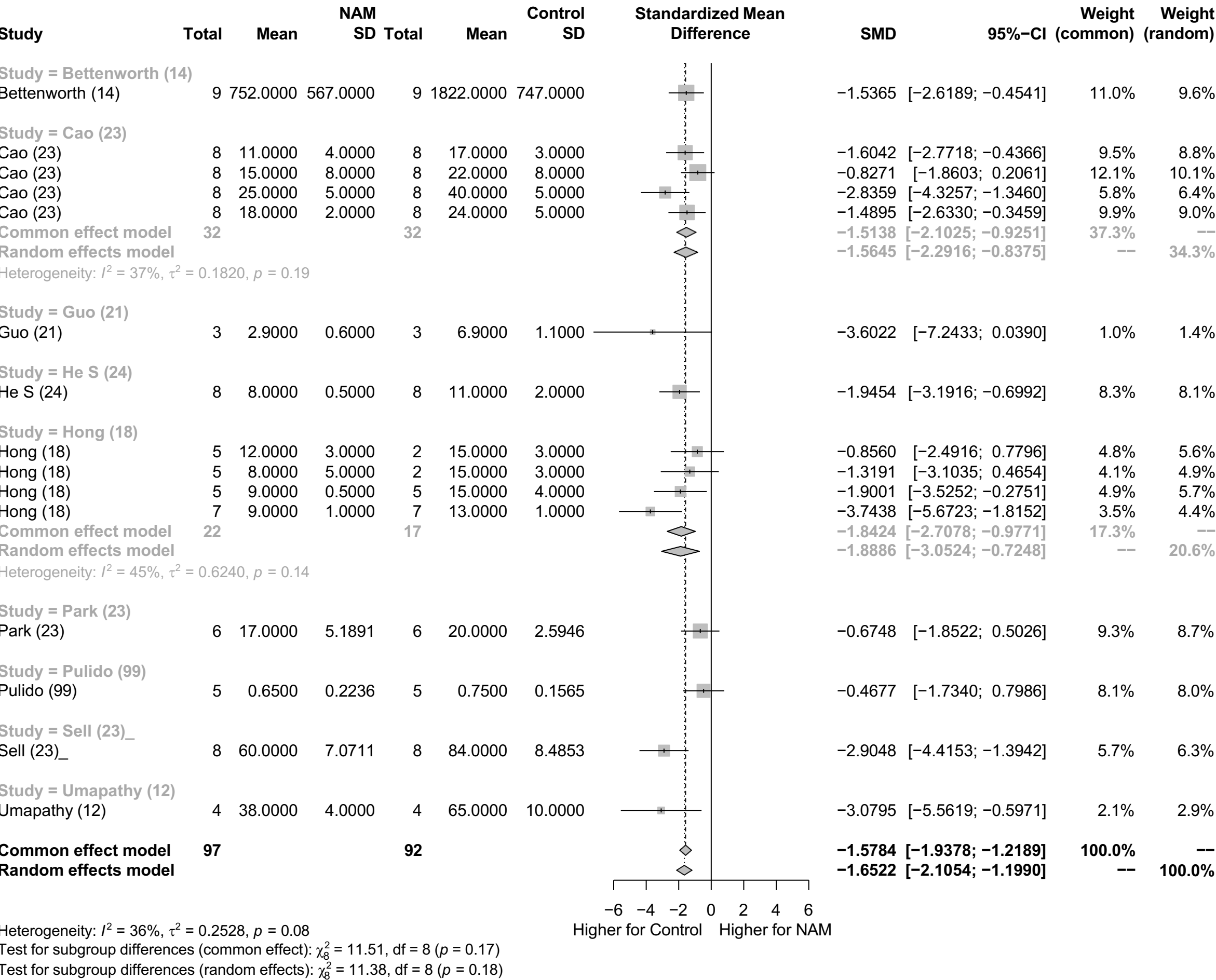

Supplement: Supplementary file 14 — Supplementary Information 14. [file 41598_2025_95735_MOESM14_ESM.pdf]

SupFigure-14. Challenge type mortality sensitivity analysis

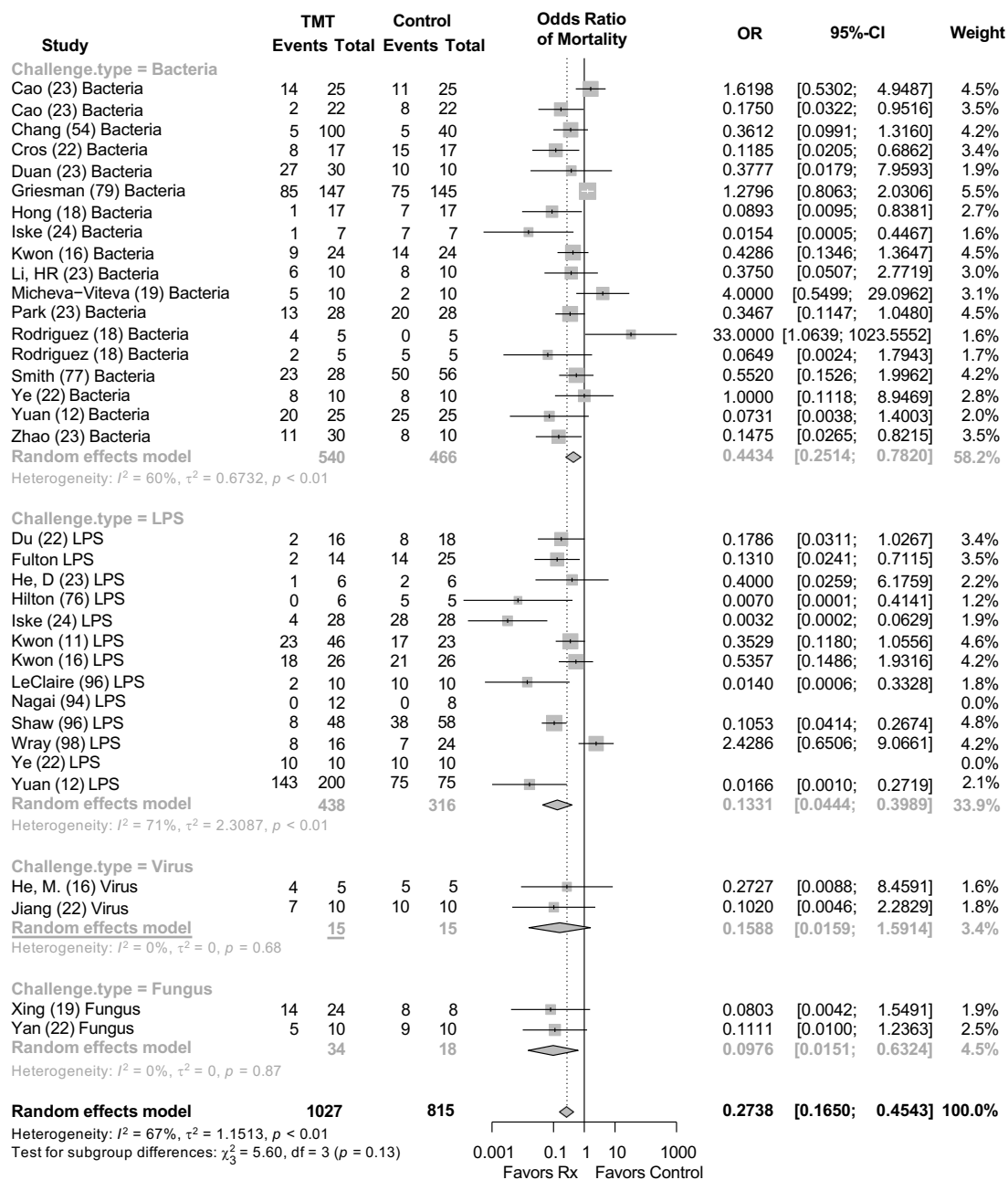

Supplement: Supplementary file 15 — Supplementary Information 15. [file 41598_2025_95735_MOESM15_ESM.pdf]

SupFigure-15. Treatment time mortality sensitivity analysis

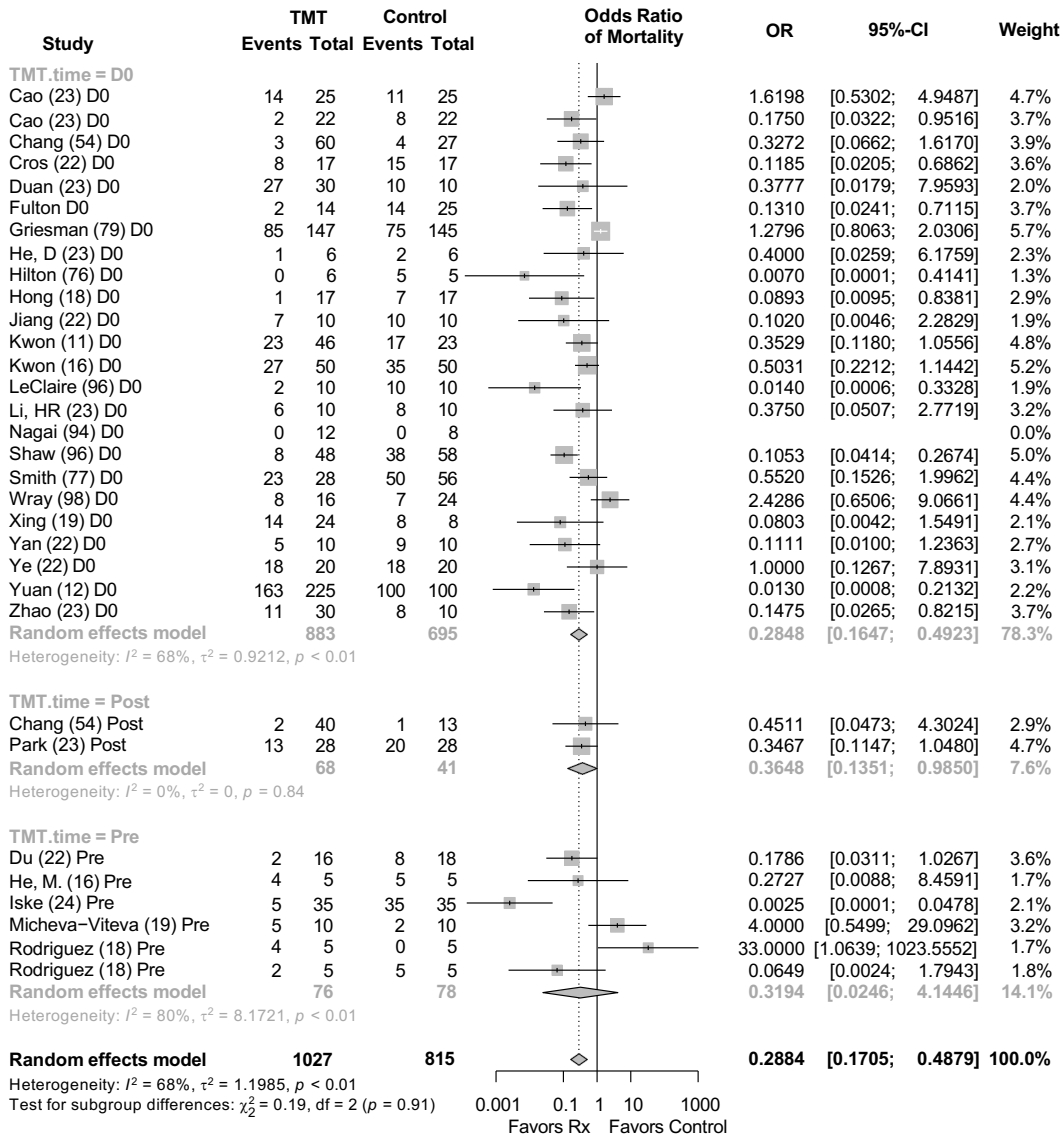

Supplement: Supplementary file 16 — Supplementary Information 16. [file 41598_2025_95735_MOESM16_ESM.pdf]

SupFigure-16. Treatment pathway type mortality sensitivity analysis

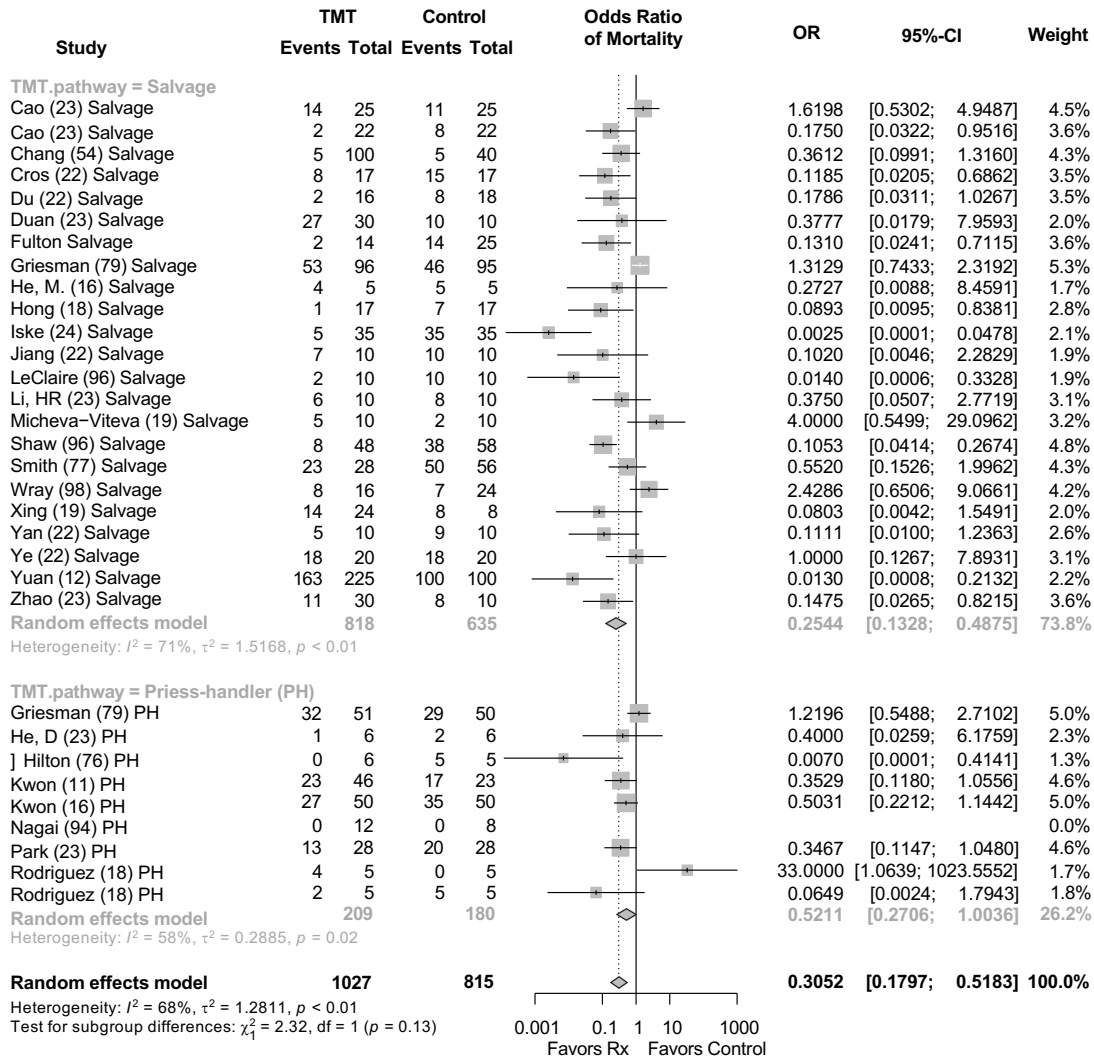

Supplement: Supplementary file 17 — Supplementary Information 17. [file 41598_2025_95735_MOESM17_ESM.pdf]

SupFigure 17

A. Chemistry measures

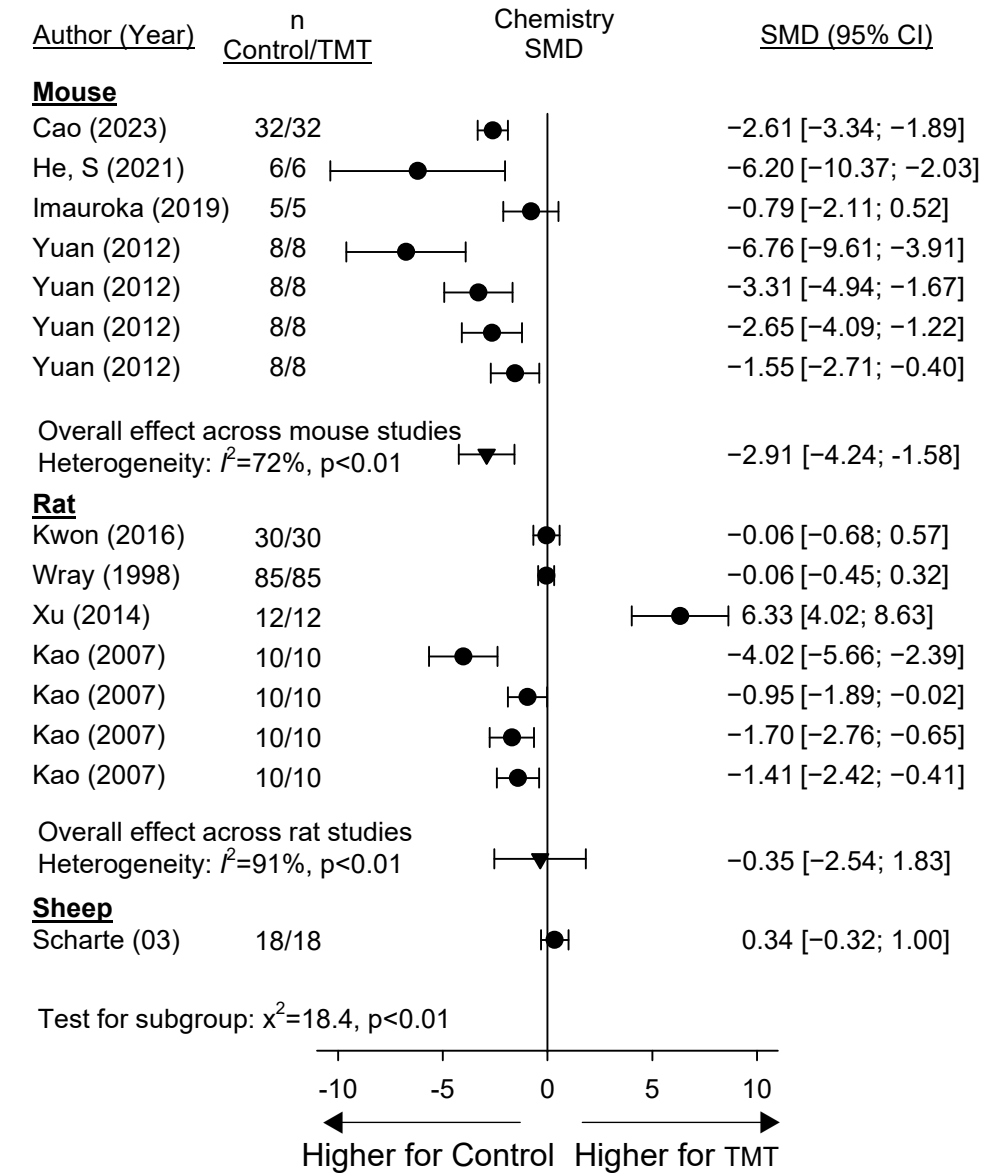

B. IL-1 $\beta$  measures

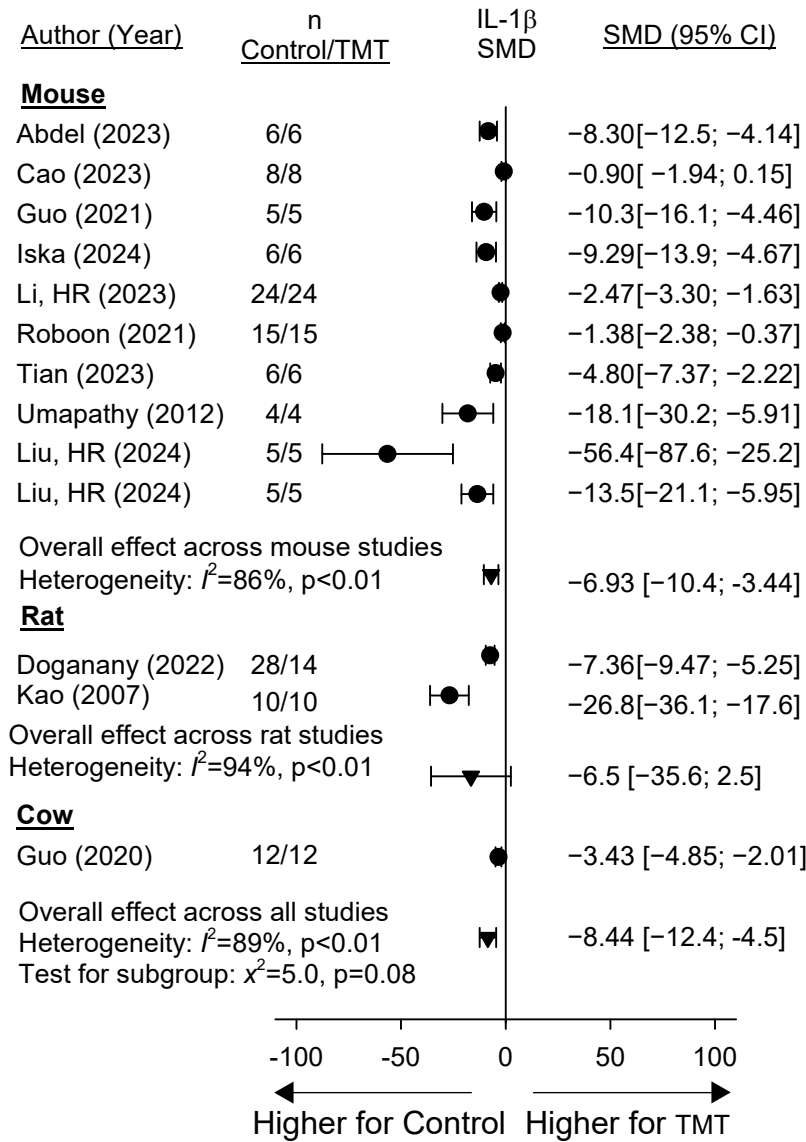

C. Myeloperoxidase measures

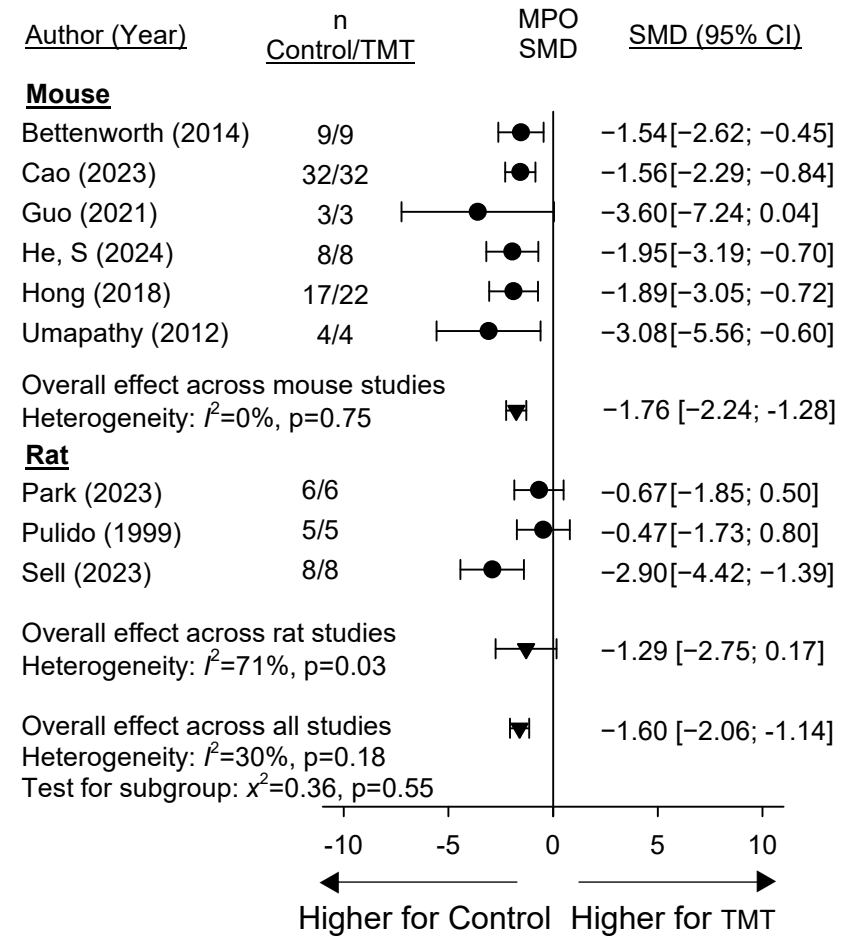

Supplement: Supplementary file 18 — Supplementary Information 18. [file 41598_2025_95735_MOESM18_ESM.pdf]

SupFigure-18. Study size mortality sensitivity analysis

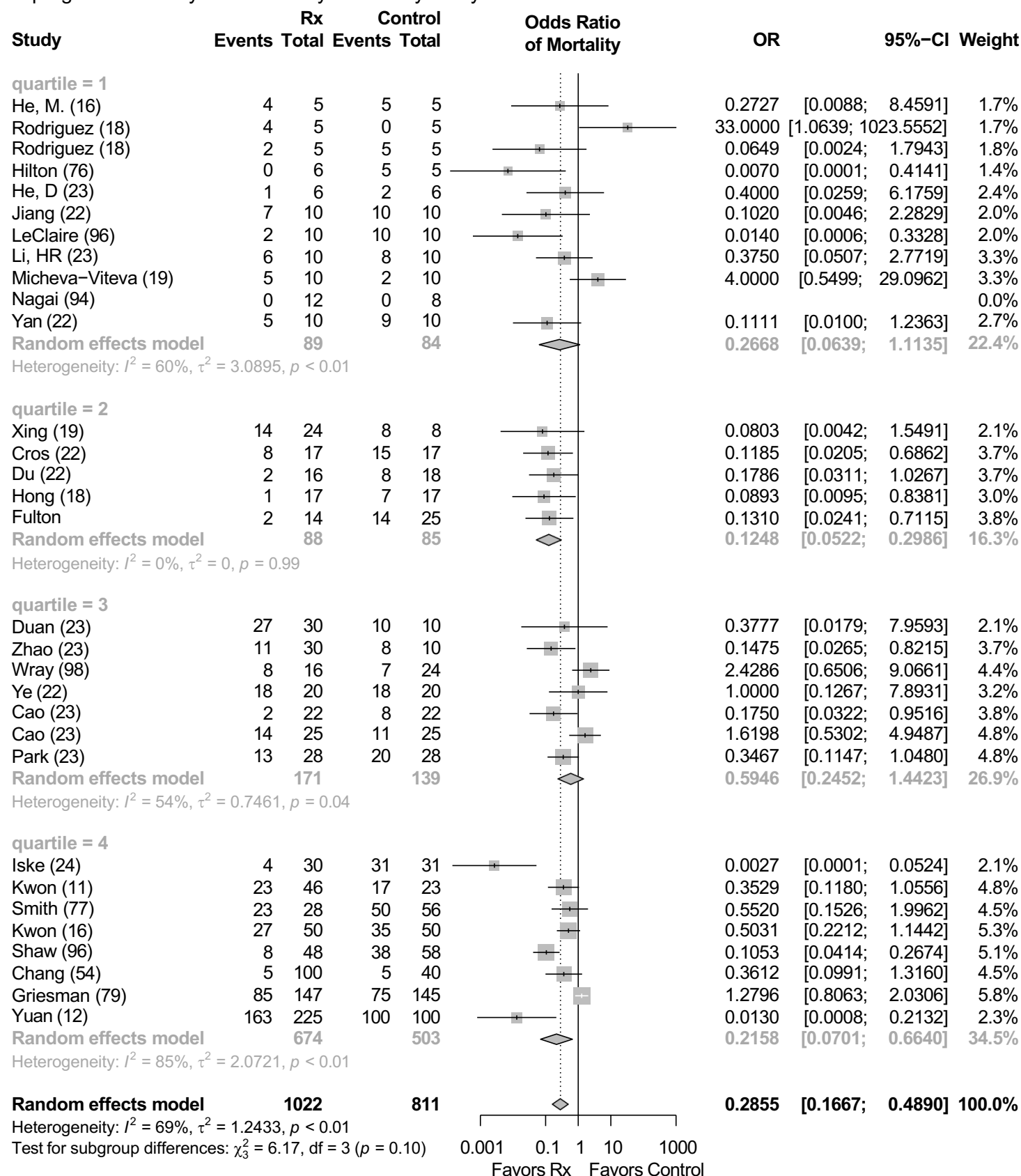

Supplement: Supplementary file 19 — Supplementary Information 19. [file 41598_2025_95735_MOESM19_ESM.pdf]
