# Supplementary material for: Meta-analysis of niacin and NAD metabolite treatment in infectious disease animal studies suggests benefit but requires confirmation in clinically relevant models
Source: Sci Rep. 2025 Apr 12;15:12621. doi: 10.1038/s41598-025-95735-y (PMC11993703; doi:10.1038/s41598-025-95735-y)
Supplement: Supplementary file 4 — Supplementary Information 4. [file 41598_2025_95735_MOESM4_ESM.pdf]

SupFigure-3. NAD metabolite by-study analysis

Study	NAM Total	NAM Mean	NAM SD	Control Total	Control Mean	Control SD	SMD	95%−CI	Weight (common)	Weight (random)
**Study = Cros (22)**										
Cros (22)	6	67.2000	2.9394	6	58.5000	4.1641	2.2274	[0.6678; 3.7870]	7.3%	6.9%
Cros (22)	6	4.1000	0.4899	6	4.8000	0.7348	−1.0343	[−2.2708; 0.2022]	11.7%	7.6%
Cros (22)	6	5.3000	0.7348	6	4.6000	0.4899	1.0343	[−0.2022; 2.2708]	11.7%	7.6%
**Common effect model**	18			18			0.5326	[−0.2300; 1.2953]	30.7%	--
**Random effects model**							0.7005	[−1.1554; 2.5563]	--	22.0%
Heterogeneity: I^2 = 82%, τ^2 = 2.2178, p < 0.01										
**Study = Du (22)**										
Du (22)	5	15.0000	1.0000	2	13.0000	2.0000	1.3859	[−0.6890; 3.4608]	4.1%	5.7%
Du (22)	5	17.0000	2.0000	2	13.0000	2.0000	1.6575	[−0.5419; 3.8569]	3.7%	5.4%
Du (22)	5	22.0000	2.0000	2	13.0000	2.0000	3.7294	[0.2620; 7.1968]	1.5%	3.4%
**Common effect model**	15			5			1.8667	[0.4829; 3.2506]	9.3%	--
**Random effects model**							1.8667	[0.4829; 3.2506]	--	14.5%
Heterogeneity: I^2 = 0%, τ^2 = 0, p = 0.51										
**Study = He, S (21)**										
He, S (21)	3	260.0000	5.0000	3	200.0000	5.0000	9.5746	[0.7352; 18.4140]	0.2%	0.8%
**Study = He, S (23)**										
He, S (23)	8	13.0000	3.5000	8	9.0000	2.5000	1.2432	[0.1467; 2.3398]	14.9%	7.9%
**Study = Izadpanah (23)**										
Izadpanah (23)	6	1.6800	0.0100	6	0.4100	0.0700	23.4377	[12.0881; 34.7874]	0.1%	0.5%
**Study = Jiang (22)**										
Jiang (22)	3	191.9000	118.8187	3	88.9000	36.1999	0.9357	[−0.8761; 2.7475]	5.4%	6.3%
**Study = Kwon (11)**										
Kwon (11)	6	130.0000	22.2390	3	65.0000	29.6520	2.3482	[0.3672; 4.3292]	4.6%	5.9%
Kwon (11)	6	240.0000	59.3041	3	65.0000	29.6520	2.9569	[0.6991; 5.2146]	3.5%	5.3%
**Common effect model**	12			6			2.6130	[1.1239; 4.1020]	8.1%	--
**Random effects model**							2.6130	[1.1239; 4.1020]	--	11.2%
Heterogeneity: I^2 = 0%, τ^2 = 0, p = 0.69										
**Study = Li, HR (23)**										
Li, HR (23)	6	245.0000	20.0000	6	145.0000	15.0000	5.2198	[2.4619; 7.9778]	2.3%	4.4%
**Study = Mo (23)**										
Mo (23)	6	6.4900	0.1100	6	6.4900	0.1200	0.0000	[−1.1316; 1.1316]	13.9%	7.8%
**Study = Nagai (94)**										
Nagai (94)	5	0.4770	0.0157	5	0.2350	0.0089	17.1370	[7.5793; 26.6947]	0.2%	0.7%
**Study = Roboon (21)**										
Roboon (21)	5	124.3000	27.9508	5	41.9000	16.7705	3.2272	[1.0542; 5.4001]	3.8%	5.5%
Roboon (21)	4	71.1000	6.6000	4	42.2000	5.6000	4.1015	[1.0283; 7.1748]	1.9%	3.9%
**Common effect model**	9			9			3.5186	[1.7444; 5.2928]	5.7%	--
**Random effects model**							3.5186	[1.7444; 5.2928]	--	9.4%
Heterogeneity: I^2 = 0%, τ^2 = 0, p = 0.65										
**Study = Zhao (23)**										
Zhao (23)	3	2.8000	0.2000	1	2.2000	0.3000	1.6926	[−2.3233; 5.7084]	1.1%	2.8%
Zhao (23)	3	3.0000	0.3000	1	2.2000	0.3000	1.5045	[−2.2126; 5.2217]	1.3%	3.1%
Zhao (23)	3	3.3000	0.3000	1	2.2000	0.3000	2.0687	[−2.5747; 6.7121]	0.8%	2.2%
**Common effect model**	9			3			1.7138	[−0.6383; 4.0658]	3.2%	--
**Random effects model**							1.7138	[−0.6383; 4.0658]	--	8.1%
Heterogeneity: I^2 = 0%, τ^2 = 0, p = 0.98										
**Study = Zingarelli (96)**										
Zingarelli (96)	5	35.0000	4.4721	5	14.0000	11.1803	2.2264	[0.4792; 3.9735]	5.9%	6.4%
**Common effect model**	**105**			**83**			**1.3795**	**[0.9568; 1.8021]**	**100.0%**	**--**
**Random effects model**							**2.0460**	**[1.2464; 2.8457]**	**--**	**100.0%**

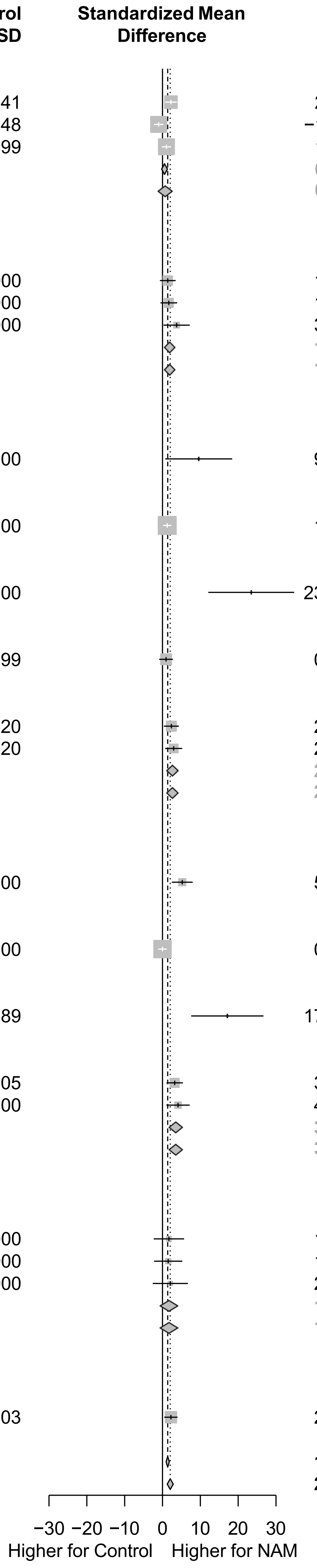

Heterogeneity: I^2 = 71%, τ^2 = 1.7968, p < 0.01
Test for subgroup differences (common effect): χ^2_{12} = 56.11, df = 12 (p < 0.01)
Test for subgroup differences (random effects): χ^2_{12} = 50.43, df = 12 (p < 0.01)
