# Supplementary material for: Meta-analysis of niacin and NAD metabolite treatment in infectious disease animal studies suggests benefit but requires confirmation in clinically relevant models
Source: Sci Rep. 2025 Apr 12;15:12621. doi: 10.1038/s41598-025-95735-y (PMC11993703; doi:10.1038/s41598-025-95735-y)
Supplement: Supplementary file 5 — Supplementary Information 5. [file 41598_2025_95735_MOESM5_ESM.pdf]

SupFigure-4. Microbe level by-study analysis

Study	NAM Total	NAM Mean	NAM SD	Control Total	Control Mean	Control SD	SMD	95%−CI	Weight (common)	Weight (random)
**Study = Bettenworth (14)**										
Bettenworth (14)	9	66.6000	66.3000	9	220.0000	130.8000	−1.4087	[−2.4675; −0.3500]	7.3%	4.2%
Bettenworth (14)	9	26.0000	13.5000	9	49.0000	15.3000	−1.5180	[−2.5969; −0.4391]	7.0%	4.1%
Bettenworth (14)	9	30.0000	9.0000	9	47.0000	9.0000	−1.7987	[−2.9342; −0.6632]	6.3%	4.1%
Bettenworth (14)	9	92.2000	66.3000	9	233.4000	130.8000	−1.2967	[−2.3360; −0.2574]	7.6%	4.2%
**Common effect model**	**36**			**36**			**−1.4935**	**[−2.0316; −0.9553]**	**28.2%**	**--**
**Random effects model**							**−1.4935**	**[−2.0316; −0.9553]**	**--**	**16.6%**
Heterogeneity: $I^2 = 0\%$, $\tau^2 = 0$, $p = 0.93$										
**Study = Cao (23)**										
Cao (23)	8	0.3500	0.2000	8	0.9000	0.1000	−3.2882	[−4.9171; −1.6592]	3.1%	3.5%
Cao (23)	8	1.1000	0.8000	8	3.3000	0.2000	−3.5665	[−5.2848; −1.8483]	2.8%	3.4%
**Common effect model**	**16**			**16**			**−3.4199**	**[−4.6021; −2.2378]**	**5.8%**	**--**
**Random effects model**							**−3.4199**	**[−4.6021; −2.2378]**	**--**	**7.0%**
Heterogeneity: $I^2 = 0\%$, $\tau^2 = 0$, $p = 0.82$										
**Study = Cros (22)**										
Cros (22)	8	0.5000	0.1697	8	1.5000	1.1314	−1.1685	[−2.2521; −0.0849]	6.9%	4.1%
**Study = Iske (24)**										
Iske (24)	3	8.9500	0.3200	3	9.0000	0.0300	−0.1755	[−1.7838; 1.4327]	3.2%	3.6%
Iske (24)	3	7.9000	0.4600	3	8.2000	0.2600	−0.6406	[−2.3434; 1.0621]	2.8%	3.5%
**Common effect model**	**6**			**6**			**−0.3948**	**[−1.5640; 0.7743]**	**6.0%**	**--**
**Random effects model**							**−0.3948**	**[−1.5640; 0.7743]**	**--**	**7.0%**
Heterogeneity: $I^2 = 0\%$, $\tau^2 = 0$, $p = 0.70$										
**Study = Jiang (22)**										
Jiang (22)	3	12.7000	0.3464	3	12.3000	0.5196	0.7227	[−1.0069; 2.4524]	2.7%	3.4%
Jiang (22)	4	35.3000	8.8000	3	45.5000	12.4708	−0.8226	[−2.4455; 0.8003]	3.1%	3.5%
Jiang (22)	4	1.1800	1.0400	3	1.0000	0.2598	0.1841	[−1.3194; 1.6876]	3.6%	3.7%
Jiang (22)	4	10.3000	0.4000	4	9.5900	0.4600	1.4308	[−0.2534; 3.1149]	2.9%	3.5%
Jiang (22)	4	8.9700	0.3600	4	8.7400	0.4000	0.5250	[−0.9047; 1.9547]	4.0%	3.8%
Jiang (22)	4	0.5300	0.0400	3	0.5800	0.0346	−1.1078	[−2.8264; 0.6108]	2.8%	3.4%
Jiang (22)	3	12.4700	0.4157	3	12.5600	0.1559	−0.2287	[−1.8425; 1.3850]	3.1%	3.6%
Jiang (22)	3	10.1600	0.3464	3	10.0200	0.6062	0.2263	[−1.3872; 1.8397]	3.1%	3.6%
**Common effect model**	**29**			**26**			**0.1275**	**[−0.4400; 0.6951]**	**25.3%**	**--**
**Random effects model**							**0.1275**	**[−0.4400; 0.6951]**	**--**	**28.4%**
Heterogeneity: $I^2 = 0\%$, $\tau^2 = < 0.0001$, $p = 0.48$										
**Study = Li (16)**										
Li (16)	4	330.0000	100.0000	4	550.0000	200.0000	−1.2086	[−2.8130; 0.3958]	3.2%	3.6%
Li (16)	4	250.0000	50.0000	4	600.0000	100.0000	−3.8456	[−6.7670; −0.9241]	1.0%	2.2%
Li (16)	4	200.0000	50.0000	4	600.0000	100.0000	−4.3949	[−7.6445; −1.1454]	0.8%	2.0%
Li (16)	4	0.6000	0.1000	4	0.8000	0.1000	−1.7373	[−3.5457; 0.0712]	2.5%	3.3%
Li (16)	4	0.5000	0.0500	4	0.9000	0.1000	−4.3949	[−7.6445; −1.1454]	0.8%	2.0%
Li (16)	4	0.3000	0.1000	4	0.8000	0.2000	−2.7468	[−5.0480; −0.4457]	1.5%	2.8%
Li (16)	4	80.0000	1.0000	4	98.0000	1.0000	−15.6353	[−26.1831; −5.0875]	0.1%	0.3%
Li (16)	4	50.0000	1.0000	4	99.0000	1.0000	−42.5627	[−71.0610; −14.0644]	0.0%	0.0%
Li (16)	4	40.0000	2.0000	4	98.0000	1.0000	−31.8633	[−53.2175; −10.5092]	0.0%	0.1%
Li (16)	4	80.0000	2.0000	1	150.0000	20.0000	−8.0206	[−16.8023; 0.7611]	0.1%	0.4%
Li (16)	4	51.0000	1.0000	1	150.0000	20.0000	−11.7155	[−24.3714; 0.9405]	0.1%	0.2%
Li (16)	4	25.0000	2.0000	1	150.0000	20.0000	−14.3225	[−29.7333; 1.0882]	0.0%	0.1%
**Common effect model**	**48**			**40**			**−2.6950**	**[−3.5983; −1.7917]**	**10.0%**	**--**
**Random effects model**							**−3.7720**	**[−5.3738; −2.1702]**	**--**	**17.2%**
Heterogeneity: $I^2 = 67\%$, $\tau^2 = 2.7685$, $p < 0.01$										
**Study = Mo (23)**										
Mo (23)	6	5.3600	0.3800	6	4.8000	1.2100	0.5762	[−0.5889; 1.7413]	6.0%	4.0%
**Study = Pacl (23)**										
Pacl (23)	7	4.7300	0.4077	8	5.8300	0.1186	−3.5651	[−5.3527; −1.7774]	2.6%	3.4%
Pacl (23)	7	5.0200	0.1853	7	6.1600	0.4077	−3.3692	[−5.1639; −1.5745]	2.5%	3.3%
**Common effect model**	**14**			**15**			**−3.4675**	**[−4.7340; −2.2010]**	**5.1%**	**--**
**Random effects model**							**−3.4675**	**[−4.7340; −2.2010]**	**--**	**6.7%**
Heterogeneity: $I^2 = 0\%$, $\tau^2 = 0$, $p = 0.88$										
**Study = Wurtele (10)**										
Wurtele (10)	6	25.0000	24.4949	6	350.0000	122.4745	−3.3956	[−5.3849; −1.4063]	2.1%	3.1%
**Study = Yan (22)**										
Yan (22)	6	0.2000	0.1483	6	2.6000	0.5189	−5.8033	[−8.8198; −2.7868]	0.9%	2.2%
Yan (22)	6	4.4000	0.8896	6	6.1000	0.6672	−1.9951	[−3.4799; −0.5103]	3.7%	3.7%
**Common effect model**	**12**			**12**			**−2.7378**	**[−4.0699; −1.4056]**	**4.6%**	**--**
**Random effects model**							**−3.6635**	**[−7.3668; 0.0398]**	**--**	**5.9%**
Heterogeneity: $I^2 = 80\%$, $\tau^2 = 5.7800$, $p = 0.03$										
**Common effect model**	**181**			**171**			**−1.2998**	**[−1.5854; −1.0141]**	**100.0%**	**--**
**Random effects model**							**−1.6574**	**[−2.2585; −1.0564]**	**--**	**100.0%**

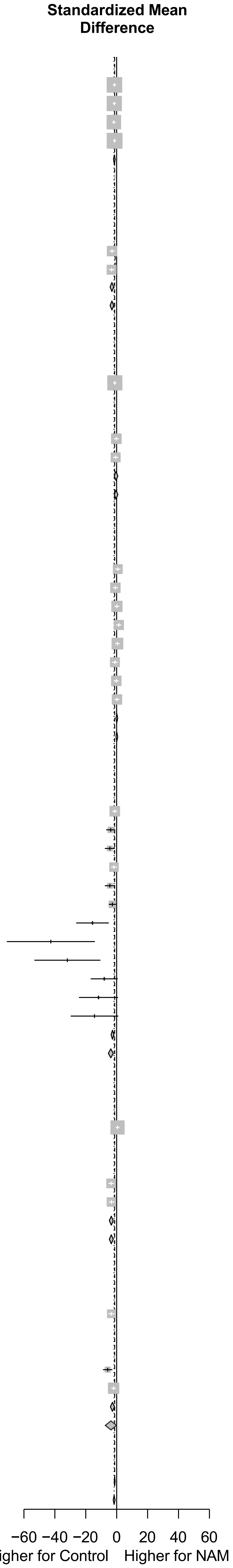

Heterogeneity: $I^2 = 73\%$, $\tau^2 = 1.9692$, $p < 0.01$
Test for subgroup differences (common effect): $\chi^2_9 = 78.63$, df = 9 ($p < 0.01$)
Test for subgroup differences (random effects): $\chi^2_9 = 75.03$, df = 9 ($p < 0.01$)
