# Supplementary material for: Meta-analysis of niacin and NAD metabolite treatment in infectious disease animal studies suggests benefit but requires confirmation in clinically relevant models
Source: Sci Rep. 2025 Apr 12;15:12621. doi: 10.1038/s41598-025-95735-y (PMC11993703; doi:10.1038/s41598-025-95735-y)
Supplement: Supplementary file 11 — Supplementary Information 11. [file 41598_2025_95735_MOESM11_ESM.pdf]

SupFigure-10. IL-1β by-study analysis

Study	NAM Total	NAM Mean	NAM SD	Control Total	Control Mean	Control SD	SMD	95%−CI	Weight (common)	Weight (random)
**Study = Abdel**										
Abdel	6	60.0000	8.0000	6	120.0000	5.0000	−8.2995	[−12.4555; −4.1435]	1.2%	4.7%
**Study = Cao (23)**										
Cao (23)	8	20.0000	20.0000	8	50.0000	40.0000	−0.8968	[−1.9390; 0.1455]	19.4%	5.7%
**Study = Doganany (22)**										
Doganany (22)	7	32.1000	3.8000	4	59.9000	4.7000	−6.1831	[−9.7157; −2.6504]	1.7%	4.9%
Doganany (22)	7	24.0000	1.6000	4	59.9000	4.7000	−11.3197	[−17.4805; −5.1589]	0.6%	3.8%
Doganany (22)	7	30.6000	2.8000	4	63.4000	7.3000	−6.4716	[−10.1478; −2.7955]	1.6%	4.9%
Doganany (22)	7	22.3000	2.1000	4	63.4000	7.3000	−8.6160	[−13.3786; −3.8534]	0.9%	4.4%
Common effect model	28			14			−7.3585	[−9.4687; −5.2482]	4.7%	---
Random effects model							−7.3585	[−9.4687; −5.2482]	---	17.9%
Heterogeneity: $I^2 = 0\%$, $\tau^2 = < 0.0001$, $p = 0.47$										
**Study = Guo (20)**										
Guo (20)	6	15.0000	5.0000	6	35.0000	5.0000	−3.6910	[−5.7989; −1.5831]	4.7%	5.4%
Guo (20)	6	8.0000	2.0000	6	19.0000	4.0000	−3.2098	[−5.1261; −1.2934]	5.7%	5.5%
Common effect model	12			12			−3.4275	[−4.8455; −2.0096]	10.5%	---
Random effects model							−3.4275	[−4.8455; −2.0096]	---	10.9%
Heterogeneity: $I^2 = 0\%$, $\tau^2 = 0$, $p = 0.74$										
**Study = Guo (21)**										
Guo (21)	5	1.0000	0.1000	5	2.8000	0.2000	−10.2766	[−16.0933; −4.4599]	0.6%	3.9%
**Study = Iska (24)**										
Iska (24)	6	840.0000	165.0000	6	4528.0000	491.0000	−9.2912	[−13.9089; −4.6736]	1.0%	4.5%
**Study = Kao (07)**										
Kao (07)	10	100.0000	31.6228	10	1500.0000	63.2456	−26.8141	[−36.0711; −17.5571]	0.2%	2.6%
**Study = Li, HR (23)**										
Li, HR (23)	6	130.0000	40.0000	6	230.0000	45.0000	−2.1674	[−3.7073; −0.6276]	8.9%	5.6%
Li, HR (23)	6	13.0000	5.0000	6	33.0000	4.5000	−3.8799	[−6.0651; −1.6946]	4.4%	5.4%
Li, HR (23)	6	14.5000	5.0000	6	28.0000	4.5000	−2.6189	[−4.3139; −0.9240]	7.3%	5.6%
Li, HR (23)	6	160.0000	25.0000	6	245.0000	50.0000	−1.9842	[−3.4656; −0.5028]	9.6%	5.6%
Common effect model	24			24			−2.4687	[−3.3035; −1.6339]	30.3%	---
Random effects model							−2.4687	[−3.3035; −1.6339]	---	22.2%
Heterogeneity: $I^2 = 0\%$, $\tau^2 = 0$, $p = 0.53$										
**Study = Liu, HR (24)**										
Liu, HR (24)	5	100.0000	2.0000	5	225.0000	2.0000	−56.4190	[−87.6440; −25.1939]	0.0%	0.4%
Liu, HR (24)	5	70.0000	2.0000	5	100.0000	2.0000	−13.5406	[−21.1306; −5.9505]	0.4%	3.2%
Common effect model	10			10			−15.9327	[−23.3080; −8.5574]	0.4%	---
Random effects model							−32.1950	[−73.8590; 9.4691]	---	3.6%
Heterogeneity: $I^2 = 85\%$, $\tau^2 = 784.8750$, $p < 0.01$										
**Study = Roboon (21)**										
Roboon (21)	6	41.4000	28.4141	6	133.5000	64.6665	−1.7015	[−3.0989; −0.3042]	10.8%	5.6%
Roboon (21)	5	53.9000	18.1122	5	133.5000	42.9325	−2.1808	[−3.9103; −0.4513]	7.0%	5.6%
Roboon (21)	4	59.7000	13.2000	4	67.3000	16.2000	−0.4468	[−1.8645; 0.9710]	10.5%	5.6%
Common effect model	15			15			−1.3563	[−2.2189; −0.4937]	28.3%	---
Random effects model							−1.3757	[−2.3827; −0.3687]	---	16.8%
Heterogeneity: $I^2 = 26\%$, $\tau^2 = 0.2057$, $p = 0.26$										
**Study = Tian (23)**										
Tian (23)	6	30.0000	3.0000	6	58.0000	7.0000	−4.7978	[−7.3716; −2.2239]	3.2%	5.3%
**Study = Umapathy (12)**										
Umapathy (12)	4	20.0000	1.0000	4	35.0000	0.2000	−18.0685	[−30.2314; −5.9057]	0.1%	1.9%
**Common effect model**	**134**			**120**			**−2.5762**	**[−3.0353; −2.1170]**	**100.0%**	**---**
**Random effects model**							**−5.8387**	**[−7.8682; −3.8092]**	**---**	**100.0%**

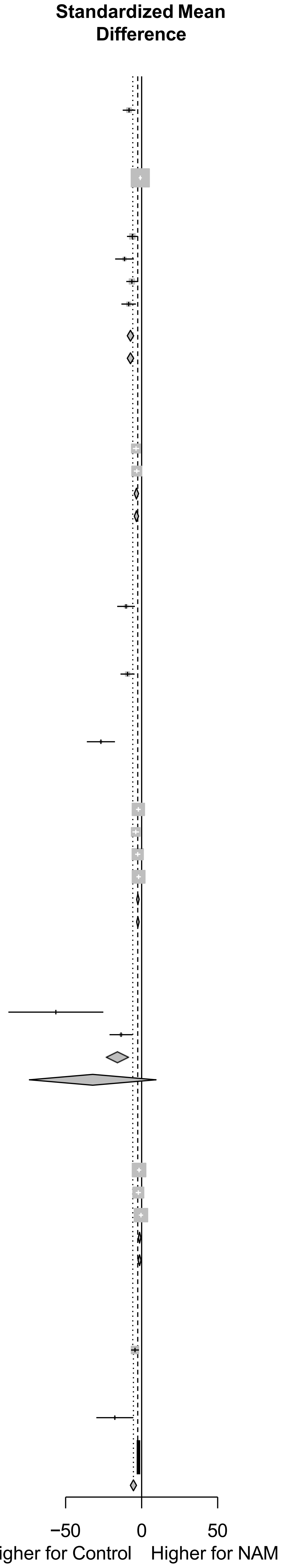

Heterogeneity: $I^2 = 83\%$, $\tau^2 = 18.5364$, $p < 0.01$
Test for subgroup differences (common effect): $\chi^2_{11} = 109.00$, df = 11 ($p < 0.01$)
Test for subgroup differences (random effects): $\chi^2_{11} = 96.08$, df = 11 ($p < 0.01$)
