# Supplementary material for: Meta-analysis of niacin and NAD metabolite treatment in infectious disease animal studies suggests benefit but requires confirmation in clinically relevant models
Source: Sci Rep. 2025 Apr 12;15:12621. doi: 10.1038/s41598-025-95735-y (PMC11993703; doi:10.1038/s41598-025-95735-y)
Supplement: Supplementary file 20 — Supplementary Information 20. [file 41598_2025_95735_MOESM20_ESM.pdf]

SupTable-1. Study summary

	Animal			Challenge			NAD Metabolite Treatment				Observation Time
**Author (year)**	**Type***	**Weight**	**Age**	**Type**	**Dose**	**Route**	**Type**	**Dose**	**Route**	**Time****	**Time##**
Abdel Rasheed (2023) [29]	Mouse Swiss alb. (F)	22–25g	10-12wk	*E. coli* 0127: B8 LPS	500µg/kg, qod X 7d	IP	Niacin	40 mg/kg/d	OG	D0	14d
Bettenworth (2014) [19]	Mouse C57BL/6 (F)	20-28g	6-8wk	*C. rodentium*	$5x10^8$ CFU	Gavage	NAM	250mg/kg	IP	2d pre	12d
Cao (2023) [30]	Mouse C57BL/6 (M)	UC	2mo	Feces	1.8g/kg or 3g/kg	IP	NMN	500mg/kg	IP	1h post	6h, 24h, or 30d
Chang (1954) [31]	Mouse (F)	20±2g	UC	*M. leprae murium*	0.5mL seed susp.	PO	NAM	0.5 % BW	PO	qd starting 1h or 1 or 2mon post	3mon
Cros (2022) [22]	Mouse C57BL/6 (M)	UC	7-12wk	CLP	NA	NA	NMN	185mg/kg	IP	On and qd for 4d post CLP	24 to 96h
Doganay (2022) [32]	Rat Wistar (M)	270± 30g	UC	CLP	NA	NA	NAD	100 or 300mg/kg	IP	qd pre x 5d and 6h post	24h
Du (2022) [33]	Mouse C57BL/6 (M)	20-25g	6-8wk	*E. coli* L2630 LPS	15mg/kg	IV	NMN	100, 300 or 500 mg/kg	IP	qd pre x 7d	12h
Duan (2023) [34]	Mouse C57BL/6 (M)	20-24g	7-8wk	CLP	NA	NA	NAM^	40µmole/kg	IV	2x daily starting 6h post	10d
Fernandes (2011) [20]	Mouse C57BL/6 (M)	UC	8-10wk	*E. coli* 0111:B4 LPS	5ug	IT	NAM	10mg/kg	IT	2h pre or post	36h
Fukuzawa (1997) [35]	Mouse Balb/c (M)	UC	8-10wk	*E.coli* LPS	2mg/kg	IP	NAM	250mg/kg	OG	1, 3 or 5h pre	45-180 min
Fulton (1974) [36]	Rat SD	250-310g	UC	*E. coli* 011:B4 LPS	10mg/kg	IV	NAM	500mg/kg	IP	30min post	24h
Griesman (1979) [37]	Mouse Swiss alb. (M and F)	20-25g	UC	*P. mirabalis*	UC	UC	NAM	10-20mg/kg x 1 or 2	IM	3.5 to 4h post	96h
Guo, W. (2020) [38]	Dairy cows	622± 62kg	UC	Endogenous + California mastitis test	NA	NA	Niacin	30g qd	PO	qd x 7d in cows with mastitis	7d
Guo, W. (2021) [39]	Mouse C57BL/6 (F)	UC	9wk	LPS (Type UC)	UC	MG	Niacin	50 mM	PO	27d from start of study	24h or 6d post delivery

Han<br>(2003) [40]	Mouse<br>C57BL/6 (M)	20-25g	7-8wk	*E. coli* 0111:B4 LPS	17mg/kg	IP	NAD$^+$	132mg/kg	IP	5 min pre and<br>12h post	18h
He, D.<br>(2023) [41]	Mouse<br>C57BL/6 (M)	UC	UC	LPS<br>(Type UC)	8 µg	IC	Niacin	18mg qd	PO	D0 and qd x 30d	28d
He, M.<br>(2016) [42]	Mouse<br>Athymic<br>homozygous<br>nude (M)	UC	4wk	KSHV transformed<br>mesenchymal<br>precursor cells	10^7cells	SC	NAM	300mg/kgqd	IP	qd starting week<br>11 post-inoculation	15wk
He, S.<br>(2022) [11]	Mouse<br>C57BL/6 (M)	20-22g	6-8wks	*E- coli* L2630 LPS	15mg/kg	IV	NMN	500mg/kg	IP	qd 7d pre	12h
He, S.<br>(2024) [12]	Mouse<br>C57BL/6 (M)	20-25g	6wk	*E. coli* LPS	15mg/kg	IP	NMN	500mg/kg	IP	Pre 1h	12h
Hilton<br>(1976) [43]	Dog<br>Mongrel	8-16kg	UC	*E. coli* 055:B5 LPS	2.5mg/kg	IV	Niacin	15mg/kg<br>7.5mg/kg	IV	15mg/kg 1.5h post<br>and 7.5mg/kg<br>2.5, 3.5 and 4.5<br>post	5h
Hong<br>(2018) [21]	Mouse<br>C57BL/6 (M)	UC	2mon	Feces or<br>LPS (Type UC)	3.75g/kg or<br>4mg/kg	IP	NR	100, 300,<br>500mg/kg	IP	30min pre	6h
Imaruoka<br>(2019) [44]	Mouse<br>MRL/lpr (F)	UC	3mon	LPS<br>(Type UC)	20ug/kg on<br>14.5dpc and<br>80ug/kg<br>on 15.5-17.5 dpc)	IP	NAM	500mg/kg	OG	qd from<br>14.5 to 17.5 dpc	18.5 dpc
Iske<br>(2024) [45]	Mouse<br>C57BL/6 (M)	UC	8-12wk	*E. coli* O111:B4 or<br>O55:B5 LPS	54 mg/kg	IP	NAD	40mg	IP	2d pre daily	10, 15, 48,<br>96,,100h
Izadpanah<br>(2023) [46]	Mice<br>transgenic<br>for hACE2<br>K18-hACE2	UC	UC	SARS-CoV-2	5 x 10^2 TCID50	IN	NR	800<br>mg/kg/day	PO	D0	14d
Jiang<br>(2022) [13]	Mouse<br>BALB-C (F)	UC	6-7wks<br>or<br>8-9mon	SARS-CoV-2<br>MASCp6 or p36	1200-6000 PFU	IN	NAD+	1mg/g/d	IP	qd x3 or 14d,<br>starting 0.5d post	3d
Kao<br>(2007) [47]	Rat<br>SD (M)	330-<br>360g	12-<br>15wks	*E. coli* 026 LPS	10mg/kg	IV	NAM	200mg/kg	IV or<br>OG	10 or 30min post	6 or 72h
Kwon, W.Y.<br>(2011) [14]	Rat,<br>SD (M)	300-<br>350g	UC	*E. coli* LPS	10mg/kg	IV	Niacin	360mg/kg<br>or<br>1180mg/kg	OG	10min post	72h

Kwon, W.Y. (2016) [48]	Rat, SD (M)	300-350g	UC	*E. coli* LPS or CLP	10mg/kg	IV	Niacin	360mg/kg	OG	10min post	72h
LeClaire (1996) [49]	Mouse Balb/c	18-20g	UC	SEB + *E. Coli* 055:B5 LPS	3ug and 70ug/mouse	IP	NAM	100ul of 200mM	IV	3h post	7 or 72h
Li, W. (2016) [50]	Mouse HBV-Tg C57BL/6 (M)	18-20g	8-12mk	Endogenous HBV	NA	NA	NAM	10, 100, 200ug/g/ BW	IV	qd for 5d	6d
Li, H.R. (2023) [15]	Mouse C57BL/6 (M)	UC	8-10 wk	CLP	NA	NA	NMN	500 mg/kg	IP	D0	7d 24h
Liu (2024) [51]	Mouse C57BL/6 (M)	UC	6-8 wk	LPS (Type UC)	5 mg/kg/bw	IP	NMN	500 mg/kg/bw	IP	7d pre daily	12h
Micheva-Viteva (2019) [52]	Mouse BALB/c (F)	UC	6wk	*B. pseudomallei K96243*	2 LD50 doses +/-Levo 25mg/kg	IN, IP	NAM	10mM	UC	2d pre and qd 24h post for 5d	21d
Mo (2023) [53]	Mouse NOD.Cg-Prkdcscid Il2rgtm1Wjl/ SzJ (NSG) (F)	UC	6-8 or 9-11wk	HIV-1_{JRFL}	10 ng p24 per mouse	IP	NMN	300 mg/kg qd	OG	4h post daily	28d
Nagai (1994) [16]	Hamster SG (M)	163±3 g	UC	*E. coli* LPS	0.01mg/ 100g BW	IT	Niacin	500 or 250 mg/kg	IP	24h and 0.5h pre, 1h post	24h
Pacl (2023) [54]	Mouse C57BL/6 (M)	UC	8-12 wk	*Mtb* H37Rv	~100 CFU	Inhaled	NAM	1 g/kg qd	PO	3d or 4wks post	4 or 8wk
Park (2023) [55]	Rat SD (M)	320-380g	UC	CLP	NA	NA	Niacin	360 mg/kg	OG	6h post	28d 24h
Pulido (1999) [56]	Rat SD (M)	250-300g	UC	*Salmonella typhi.* LPS	20mg/kg	IP	NAM	200mg/kg	IP	90min post	6h
Roboon (2021) [17]	Mouse ICR WT (M)	30-36g	10-11wk	LPS (Type UC)	10ug at 0d and 1h post final NR dose	IC	NR	400mg/kg	IP	qd for 7d pre	6h
Rodriguez (2018) [57]	Mouse C57BL/6	UC	8-10wk	*L. monocytogenes*	1 x 10^7CFU or 1 x 10^8CFU	IP	NAD+	40mg	IP	qd 5d pre, or qd 2d pre and qd post	10 or 15d
Scharte (2003) [58]	Sheep (F)	40.6± 1.6kg	adult	*S. typhosa* LPS	10ng/kg/min x 24h	IV	NAM	40mg/kg/5min & 10mg/kg/h	IV	1h pre and up to 24h post	24h
Selli (2023) [59]	Rat SD (F)	210-230g	UC	CLP	NA	NA	NR	500mg/kg	IP	30min pre & 12h post	24h
Shaw (1966) [60]	Rat SD	150-300g	UC	*E. coli* 0111:B4 LPS	10mg/kg	IV	NAM	500mg/kg	IV	With or 30min post LPS	24h

Shi (2017) [61]	Mouse C57BL/6	UC	4-6wk	LPS (Type UC)	10mg/kg	UC	Niacin	200mg/kg	OG	With LPS	4h
Smith (1977) [62]	Mouse	28-36g	UC	*S. aureus* 13709	0.1ml of 10^9CFU/ml	IP	NAM	1/16 of LD_{50} dose	SC	1h post	31h
Tian (2023) [63]	Mouse C57BL/6 (M)	UC	8-10wk	LPS (Type UC)	10mg/kg	IT	NMN	500mg/kg	IP	UC	24h
Umapathy (2012) [64]	Mouse C57bl/6 (F)	20-25g	8-10wk	*E. coli* 0111:B4 LPS	0.9mg/kg	IT	NAD^+	5.46mg/kg	IV	15min post	24h
Wray (1998) [65]	Rat (M)	240-320g	UC	*E. coli* LPS	10mg/kg over 20min	IV	NAM	10mg/kg/h	IV	15min pre	6h
Wurtele (2010) [66]	Mouse A/J	UC	8-12wk	*C. albicans*	3x10^5cells	IV	NAM	500mg/kg	IP	30min pre and 8h post or 9h apart for 24h for 1 or 3d	24 or 120h
Xing (2019) [67]	Mouse BALB/c (F)	UC	6wk	*C. albicans* SC5314	5x10^6cells	IV	NAM	1.64, 3.28 or 6.56 mmol/kg	IP	qd 0, 1, 2, 4, 6d post	6d
Xu (2014) [68]	Rat SD (M)	180-220g	UC	CLP	NA	NA	NAM	60mg qd	OG	qd x 3d pre	24h
Yan (2022) [69]	Mouse BALB/c (F)	UC	6wk	*C. albicans* SC5314	5x10^6cells in 200ul NS	IV	NAM	3.28 mmol/kg	IP	qd 0, 1, 2, 4, 6d post	4 or 30d
Ye (2022) [70]	Mice Balb/c (F)	UC	8-12wk	*E. coli* LPS or CLP+ *Pseudomonas*	LPS 7.5 mg/kg PA 1x10^8 CFU	IV or IT	NAD	20 mg/kg−1	IV	1h post or 1h & 24h post	15d 24h
Yuan (2012) [71]	Mouse BALB/c (M)	20-25g	UC	*E. coli* 055:B5 LPS +/- D-Gal or CLP	LPS 10 or 20 and D-G 700mg/kg	IP	NAM	100, 200 or 400 mg/kg	IP	0.5h pre and/or 1h to 2d post	1.5 to 7d
Zhao (2023) [72]	Mouse C57BL/6 (M)	18-22g	6-8wk	CLP	NA	NA	NR	100, 500, or 1000 mg/kg	IP	0h	24h or 7d
Zingarelli (1996) [73]	Rat Wistar (M)	280-300g	UC	*E. coli* LPS	15mg/kg	IV	NAM	200mg/kg, then 10mg/kg/h	IV	1h post	3h

Alb. – albino; BW – body weight; CFU – bacterial or fungal colony forming units; CLP – cecal ligation and puncture; d – day; D-Gal – D-galactosamine; dpc – days post coitus; F – female; h – hour; HBV-TG – transgenic hepatitis B virus producing mice; IC – intracerebral; IP – intraperitoneal; IT – intratracheal; IV – intravenous; KSHV – kaposi sarcoma associated herpes virus; Levo – levofloxacin; LPS – lipopolysaccharide; M – male; mos – months; NA – not applicable; NAD^+ – nicotinamide dinucleotide; NAM – nicotinamide; NCA – niacinamide; NMN – nicotinamide mononucleotide; NR – nicotinamide riboside; OG – oral gastric; PFU – plaque forming units; PO – by mouth; post – time after challenge; pre – time before challenge; qd – every day; qod – every other day; SC – subcutaneous; SD – Sprague Dawley; SEB – *S. aureus* enterotoxin B; SG – Syrian Golden; susp – suspension; UC – unclear

*Unless related to generation of infectious challenge, experimental groups analyzed included only those with wild type animals; **timing of treatment relative to challenge; ##time of measure analyzed post challenge; agents tested NAM, NMN and NR. LPS source noted in the table above, unless not provided by the report.
