# Supplementary material for: Meta-analysis of niacin and NAD metabolite treatment in infectious disease animal studies suggests benefit but requires confirmation in clinically relevant models
Source: Sci Rep. 2025 Apr 12;15:12621. doi: 10.1038/s41598-025-95735-y (PMC11993703; doi:10.1038/s41598-025-95735-y)
Supplement: Supplementary file 21 — Supplementary Information 21. [file 41598_2025_95735_MOESM21_ESM.pdf]

SupTable-2. Non-survivor data*									
**Author (year)**	**Animal**	**Challenge type#**	TMT Type	TMT metabolism pathway	Initial TMT time**	Control total N	Control non-survivor N	TMT Total N	TMT non-survivor N
Cao (2023)	Mouse	Bacteria	NMN	Salvage	D0	25	11	25	14
	Mouse	Bacteria	NMN	Salvage	D0	22	8	22	2
Chang (1954)	Mouse	Bacteria	NAM	Salvage	D0	20	3	20	1
	Mouse	Bacteria	NAM	Salvage	D0			20	2
	Mouse	Bacteria	NAM	Salvage	D0	20	2	20	0
	Mouse	Bacteria	NAM	Salvage	Post			20	1
	Mouse	Bacteria	NAM	Salvage	Post			20	1
Cros (2022)	Mouse	Bacteria	NMN	Salvage	D0	17	15	17	8
Du (2022)	Mouse	LPS	NMN	Salvage	Pre	18	8	16	2
Duan (2023)	Mouse	Bacteria	NAM	Salvage	D0	10	10	10	10
	Mouse	Bacteria	NMN	Salvage	D0			10	9
	Mouse	Bacteria	NR	Salvage	D0			10	8
Fulton (1974)	Rat	LPS	NAM	Salvage	D0	25	14	14	2
Griesman (1979)	Mouse	Bacteria	NAM	Salvage	D0	43	24	43	26
	Mouse	Bacteria	NAM	Salvage	D0	52	22	53	27
	Mouse	Bacteria	Niacin	PH	D0	50	29	51	32
He, D (2023)	Mouse	LPS	Niacin	PH	D0	6	2	6	1
He, M. (2016)	Mouse	Virus	NAM	Salvage	Pre	5	5	5	4
Hilton (1976)	Dog	LPS	Niacin	PH	D0	5	5	6	0
Hong (2018)	Mouse	Bacteria	NR	Salvage	D0	17	7	17	1
Iske (2024)	Mouse	Bacteria	NAD	Salvage	Pre	7	7	7	1
	Mouse	LPS	NAD	Salvage	Pre	6	6	6	1
	Mouse	LPS	NAD	Salvage	Pre	6	6	6	1
	Mouse	LPS	NAD	Salvage	Pre	5	5	5	0
	Mouse	LPS	NAD	Salvage	Pre	6	6	6	1
Jiang (2022)	Mouse	Virus	NMN	Salvage	D0	10	10	10	7
Kwon (2011)	Rat	LPS	Niacin	PH	D0	23	17	23	14
	Rat	LPS	Niacin	PH	D0			23	9

Kwon (2016)	Rat	LPS	Niacin	PH	D0	26	21	26	18
	Rat	Bacteria	Niacin	PH	D0	24	14	24	9
LeClaire (1996)	Mouse	LPS	NAM	Salvage	D0	10	10	10	2
Li, HR (2023)	Mouse	Bacteria	NMN	Salvage	D0	10	8	10	6
Micheva-Viteva (2019)	Mouse	Bacteria	NAM	Salvage	Pre	5	2	5	5
	Mouse	Bacteria	NAM	Salvage	Pre	5	0	5	0
Nagai (1994)	Hamster	LPS	Niacin	PH	D0	8	0	6	0
		LPS	Niacin	PH	D0			6	0
Park (2023)	Rat	Bacteria	Niacin	PH	D0	28	20	28	13
Rodriguez (2018)	Mouse	Bacteria	NAD	Salvage	Pre	5	0	5	4
	Mouse	Bacteria	NAD	Salvage	Pre	5	5	5	2
Shaw (1996)	Rat	LPS	NAM	Salvage	D0	58	38	27	2
	Rat	LPS	NAM	Salvage	D0			21	6
Smith (1977)	Mouse	Bacteria	NAM	Salvage	D0	56	50	28	23
Wray (1998)	Rat	LPS	NAM	Salvage	D0	24	7	16	8
Xing (2019)	Mouse	Fungus	NAM	Salvage	D0	8	8	8	8
	Mouse	Fungus	NAM	Salvage	D0			8	6
	Mouse	Fungus	NAM	Salvage	D0			8	0
Yan (2022)	Mouse	Fungus	NAM	Salvage	D0	6	6	6	5
	Mouse	Fungus	NAM	Salvage	D0	4	3	4	0
Ye (2022)	Mouse	Bacteria	NAD	Salvage	D0	10	10	10	10
	Mouse	Bacteria	NAD	Salvage	D0	10	8	10	8
Yuan (2012)	Mouse	LPS	NAM	Salvage	D0	25	25	25	23
	Mouse	LPS	NAM	Salvage	D0			25	18
	Mouse	LPS	NAM	Salvage	D0			25	6
	Mouse	LPS	NAM	Salvage	D0	25	25	25	16
	Mouse	LPS	NAM	Salvage	D0			25	22
	Mouse	LPS	NAM	Salvage	D0			25	25
	Mouse	LPS	NAM	Salvage	D0	25	25	25	11
	Mouse	LPS	NAM	Salvage	D0			25	22
	Mouse	Bacteria	NAM	Salvage	D0	25	25	25	20
	Mouse	Bacteria	NR	Salvage	D0			10	4

Zhao (2023)	Mouse	Bacteria	NR	Salvage	D0	10	8	10	4
	Mouse	Bacteria	NR	Salvage	D0			10	3

LPS – lipopolysaccharide; N – number of animals; NAD – nicotinamide adenine dinucleotide; NMN – nicotinamide mononucleotide; NR – nicotinamide riboside; PH – Preiss-Handler NAD biosynthesis pathway; Rx – treatment; Salvage – Salvage NAD biosynthesis pathway

*See SupTable-1 for more detailed information about challenge and treatment regimens and measurement times; **Rx Time – ≥ 1 day before challenge = pre; day of challenge = D0; ≥1 day after challenge = post; # – Bacteria included single types of bacterial challenge as well as cecal ligation and fecal challenges
