# Supplementary material for: Meta-analysis of niacin and NAD metabolite treatment in infectious disease animal studies suggests benefit but requires confirmation in clinically relevant models
Source: Sci Rep. 2025 Apr 12;15:12621. doi: 10.1038/s41598-025-95735-y (PMC11993703; doi:10.1038/s41598-025-95735-y)
Supplement: Supplementary file 22 — Supplementary Information 22. [file 41598_2025_95735_MOESM22_ESM.pdf]

SupTable-3. Measured NAD metabolite data*

Author (Year)	Animal Type	Challenge Type	Rx Type	Initial Rx time **	Metabolite type#	Metabolite Source	Measure type	Variance type	Control N	Control measure	Control variance	Rx N	Rx measure	Rx variance
Cros (2022)	mouse	Bacteria	NMN	D0	NAD^+	Blood	Mean	SEM	6	58.5	1.7	6	67.2	1.2
	mouse	Bacteria	NMN	D0	NAD^+	Heart	Mean	SEM	6	4.8	0.3	6	4.1	0.2
	mouse	Bacteria	NMN	D0	NAD^+	Lung	Mean	SEM	6	4.6	0.2	6	5.3	0.3
Du (2022)	mouse	LPS	NMN 100	Pre	NAD^+	Lung	Mean	SD	5	13	2	5	15	1
	mouse	LPS	NMN 300	Pre	NAD^+	Lung	Mean	SD				5	17	2
	mouse	LPS	NMN 500	Pre	NAD^+	Lung	Mean	SD				5	22	2
He, S (2021)	mouse	LPS	NMN	Pre	NAD^+	Kidney	Mean	SD	3	200	5	3	260	5
He, S (2024)	mouse	LPS	NMN	D0	NAD	Lung	Mean	SD	8	9.0	2.5	8	13.0	3.5
Izadpanah (2023)	mouse	virus	NR	D0	NAM	plasma	Mean	SD	6	0.41×10^9	0.07×10^9	6	1.68×10^9	0.01×10^9
Jiang (2022)	mouse	virus	NMN	D0	NAD^+	Lung	Mean	SEM	3	88.9	20.9	3	191.9	68.6
Kwon (2011)	Rat	LPS	Niacin 360	D0	NAD^+	Lung	Median	IQR	6	65	50, 90	6	130	110, 140
	Rat	LPS	Niacin 1180	D0	NAD^+	Lung	Median	IQR				6	240	220, 300
Li, HR (2023)	mouse	Bacteria	NMN	D0	NAD	Brain	Mean	SD	6	145	15	6	245	20
Mo (2023)	mouse	virus	NMN	D0	NAD	Plasma	Mean	SD	6	Log_{10} 6.49	Log_{10} 0.12	6	Log_{10} 6.49	Log_{10} 0.11
Nagai (1994)	hamster	LPS	Niacin 500	Pre	NAD	Lung	Mean	SEM	5	0.235	0.004	5	0.477	0.007
Roboon (2021)	mouse	LPS	NR	Pre	NAD^+	Brain	Mean	SEM	5	41.9	7.5	5	124.3	12.5
	mouse	LPS	NR	D0	NAD^+	Brain	Mean	SEM	4	42.2	2.8	4	71.1	3.3
Zhao (2023)	mouse	Bacteria	NR 100	D0	NAD	T cells	Mean	SD	3	2.2	0.3	3	2.8	0.2
	mouse	Bacteria	NR 500	D0	NAD	T cells	Mean	SD				3	3.0	0.3
	mouse	Bacteria	NR 1000	D0	NAD	T cells	Mean	SD				3	3.3	0.3
Zingarelli (1996)	rat	LPS	NAM	D0	NAD	Mac	Mean	SEM	4-6	14	5	4-6	35	2

LPS – lipopolysaccharide; Mac – macrophage; Rx – treatment; NAD – nicotinamide adenine dinucleotide; N – number of animals; NMN – nicotinamide mononucleotide; NR – nicotinamide riboside; SD – standard deviation; IQR – 25 to 75% quartiles; SEM – standard error of the mean; T cells – circulating T cells; *See SupTable-1 for more detailed information about challenge and treatment regimens and measurement times; **Rx Time – ≥ 1 day before challenge = pre; day of challenge = D0; ≥1 day after challenge = post; # – NAD metabolite measured
