# Supplementary material for: Meta-analysis of niacin and NAD metabolite treatment in infectious disease animal studies suggests benefit but requires confirmation in clinically relevant models
Source: Sci Rep. 2025 Apr 12;15:12621. doi: 10.1038/s41598-025-95735-y (PMC11993703; doi:10.1038/s41598-025-95735-y)
Supplement: Supplementary file 23 — Supplementary Information 23. [file 41598_2025_95735_MOESM23_ESM.pdf]

SupTable-4. Microbe level data*

Author (year)	Animal	Challenge type	Rx Type	Initial Rx Time**	Parameter	Measure type	Variance type	Control N	Control measure	Control variance	Rx N	Rx measure	Rx variance
Bettenworth (2014)	Mouse	Bacteria	NAM	post	Fecal, Log$_{10}$CFU/g	Mean	SE	9	7.1x10^{7}	1.4x10^{1}	9	1.2x10^{5}	1.2x10^{1}
	Mouse	Bacteria	NAM	post	CFU# spleen/MLN	Mean	SE	9	220	43.6	9	66.6	22.1
	Mouse	Bacteria	NAM	post	% mice infected organs	Mean	SE	9	49	5.1	9	26	4.5
	Mouse	Bacteria	NAM	pre	Fecal, Log$_{10}$CFU/g	Mean	SE	9	1.2x10^{8}	1.7x10^{1}	9	1.3x10^{5}	1.4x10^{1}
	Mouse	Bacteria	NAM	pre	% mice infected organs	Mean	SE	9	47	3	9	30	3
	Mouse	Bacteria	NAM	pre	CFU# spleen/MLN	Mean	SE	9	233.4	43.6	9	92.2	22.1
Cao (2023)	Mouse	Bacteria	NMN	D0	CFUx10^{8}/ml peritoneal fluid	Mean	SD	8	0.9	0.1	8	0.35	0.2
	Mouse	Bacteria	NMN	D0	CFUx10^{6}/ml blood	Mean	SD	8	3.3	0.2	8	1.1	0.8
Cros (2022)	Mouse	CLP	NMN	D0	Bac DNA pg/ml	Mean	SEM	8	1.5	0.4	8	0.5	0.06
Iske (2024)	Mouse	Bacteria	NAD	Pre	Log$_{10}$CFU/g kidney	Mean	SD	3	9	0.03	3	8.95	0.32
	Mouse	Bacteria	NAD	Pre	Log$_{10}$CFU/g liver	Mean	SD	3	8.2	0.26	3	7.9	0.46
Jiang (2022)	Mouse 8-9m	Virus MASCp36	NAD+	D0	Virus Log$_{10}$ copies/g lung	Mean	SEM	3	12.3	0.3	3	12.7	0.2
	Mouse 8-9m	Virus p36	NAD+	D0	% infected lung area	Mean	SEM	3	45.5	7.2	4	35.3	4.4
	Mouse 8-9m	Virus p36	NAD+	D0	Relative intensity	Mean	SEM	3	1.0	0.15	4	1.18	0.52
	Mouse 6-7wk	Virus p6	NAD+	D0	Virus Log$_{10}$ copies/g Lung	Mean	SEM	4	9.59	0.23	4	10.3	0.20
	Mouse 6-7wk	Virus p6	NAD+	D0	Virus copies/g trachea	Mean	SEM	4	8.74	0.2	4	8.97	0.18
	Mouse 6-7wk	Virus p6	NAD+	D0	% infected alveolar area	Mean	SEM	3	0.58	0.02	4	0.53	0.02
	Mouse 8-9m	Virus p36	NMN	D0	Virus copies/g lung	Mean	SEM	3	12.56	0.09	3	12.47	0.24
	Mouse 8-9m	Virus p36	NMN	D0	Virus copies/g trachea	Mean	SEM	3	10.02	0.35	3	10.16	0.20

Li (2016)	Mouse	Virus	NAM 10	Post	Copies/10ul serum	Mean	SD	4	5.5x10^5	2.0 x10^5	4	3.3x10^5	1.0 x10^5
	Mouse	Virus	NAM 100	Post	Copies/10ul serum	Mean	SD	4	6.0x10^5	1.0 x10^5	4	2.5x10^5	0.5 x10^5
	Mouse	Virus	NAM 200	Post	Copies/10ul serum	Mean	SD	4	6.0x10^5	1.0 x10^5	4	2.0x10^5	0.5 x10^5
	Mouse	Virus	NAM 10	Post	HBsAg secretion %	Mean	SD	4	0.80	0.10	4	0.60	0.10
	Mouse	Virus	NAM 100	Post	HBsAg secretion %	Mean	SD	4	0.90	0.10	4	0.50	0.05
	Mouse	Virus	NAM 200	Post	HBsAg secretion %	Mean	SD	4	0.80	0.20	4	0.30	0.10
	Mouse	Virus	NAM 10	Post	HBeAg secretion%	Mean	SD	4	98	1	4	80	1
	Mouse	Virus	NAM 100	Post	HBeAg secretion%	Mean	SD	4	99	1	4	50	1
	Mouse	Virus	NAM 200	Post	HBeAg secretion%	Mean	SD	4	98	1	4	40	2
	Mouse	Virus	NAM 10	Post	Copies/mg liver tissue	Mean	SD	4	1.5 x10^5	0.2x10^5	4	8x10^4	0.2x10^4
	Mouse	Virus	NAM 100	Post	Copies/mg liver tissue	Mean	SD				4	5.1x10^4	0.1x10^4
	Mouse	Virus	NAM 200	Post	Copies/mg liver tissue	Mean	SD				4	2.5x10^4	0.2x10^4
Micheva-Viteva (2019)	Mouse	Bacteria	NAM	Pre	CFU/g liver	Median	IQR	5	0	5	4	5x10^1	25
	Mouse	Bacteria	NAM	Pre	CFU/g spleen	Median	IQR	5	2x10^2	250	4	2x10^8	5x10^8
	Mouse	Bacteria	NAM	Pre	CFU/g lung	Median	IQR	5	3x10^1	75	4	2x10^3	2.5x10^3
Mo (2023)	Mouse	Virus	NMN	D0	Plasma log copies/mL	Mean	SD	6	4.80	1.21	6	5.36	0.38
Pacl (2023)	Mouse	Bacteria	NAM	Post	Lung Log$_{10}$ CFU at 4wks	Median	IQR	8	5.83 Log 10	(5.71, 5.87)	7	4.73 Log 10	(4.25, 4.80)
	Mouse	Bacteria	NAM	Post	Lung Log$_{10}$ CFU at 8wks	Median	IQR	7	6.16 Log 10	(5.94, 6.49)	7	5.02 Log 10	(4.86, 5.11)
Wurtele (2010)	Mouse	Fungus	NAM	D0	CFU/Kidney	Mean	SEM	6	3.5 x10^5	0.5x10^5	6	2.5 x10^4	1.0x10^4

Yan (2022)	Mouse	Fungus	NAM +AmB	D0	Log_{10}CFU/mg kidney	Median	IQR	6	2.6 Log 10	2,2.7 Log10	6	0.2 Log10	0.1,0.3 Log10
	Mouse	Fungus	NAM+ NS	D0	Log_{10}CFU/mg kidney	Median	IQR	6	6.1 Log10	5.5, 6.4 Log10	6	4.4 Log10	4.1, 5.3 Log10
Zhao (2023)	Mouse	Bacteria	NAM 100	D0	CFU/10^3/ml peritoneal fluid	Mean	SD	6	1400	300	6	600	150
	Mouse	Bacteria	NAM 500	D0	CFU/10^3/ml peritoneal fluid	Mean	SD				6	125	50
	Mouse	Bacteria	NAM 1000	D0	CFU/10^3/ml peritoneal fluid	Mean	SD				6	135	60
	Mouse	Bacteria	NAM 100	D0	CFU/10^3/ml blood	Mean	SD	6	1250	225	6	495	175
	Mouse	Bacteria	NAM 500	D0	CFU/10^3/ml blood	Mean	SD				6	275	100
	Mouse	Bacteria	NAM 1000	D0	CFU/10^3/ml blood	Mean	SD				6	250	125

AmB – amphotericin B; BAL – bronchoalveolar lavage; CLP – cecal ligation and puncture; CFU – colony forming unit; Fung – fungus; GAL – D-galactosamine; HBeAg and HBsAg – hepatitis B e and s antigen; HPC – hippocampal; IQR – 25 to 75% quartiles; LPS – lipopolysaccharide; MLN – mesenteric lymph nodes; N – number of animals; NAD – nicotinamide adenine dinucleotide; NMN – nicotinamide mononucleotide; NR – nicotinamide riboside; NS – normal saline; Rx – treatment group; SD – standard deviation; SEM – standard error of the mean

**Rx Time – ≥ 1 day before challenge = pre, day of challenge = D0, ≥1 day after challenge = post

*See SupTable-1 for more detailed information about challenge and treatment regimens and measurement times.
