# Supplementary material for: Meta-analysis of niacin and NAD metabolite treatment in infectious disease animal studies suggests benefit but requires confirmation in clinically relevant models
Source: Sci Rep. 2025 Apr 12;15:12621. doi: 10.1038/s41598-025-95735-y (PMC11993703; doi:10.1038/s41598-025-95735-y)
Supplement: Supplementary file 24 — Supplementary Information 24. [file 41598_2025_95735_MOESM24_ESM.pdf]

SupTable-5. Histologic organ injury data*

Author (year)	Animal Type	Challenge Type	Rx Type	Initial Rx time**	Parameter	Measure type	Variance type	Control N	Control measure	Control variance	Rx N	Rx measure	Rx variance
Abdel (2023)	Mouse	LPS	Niacin	D0	Brain injury score	Mean	SD	3	7.5	1.5	3	1.0	1.0
Bettenworth (2014)	Mouse	Bacteria	NAM	Post	Colonic injury score	Mean	SE	9	2.5	0.3	9	1.3	0.4
	Mouse	Bacteria	NAM	Post	Crypt length	Mean	SE	9	17.2	1.1	9	13.4	0.7
	Mouse	Bacteria	NAM	Pre	Colonic injury score	Mean	SE	9	2	0.4	9	1	0.3
	Mouse	Bacteria	NAM pre	Pre	Colonic injury score	Mean	SE	9	15	1	9	12	1
Du (2022)	Mouse	LPS	NMN	Pre	LIS	Mean	SD	18	9	3	16	4	2
	Mouse	LPS	NMN	Pre	LIS	Mean	SD	5	10	3	5	5	2
Guo (2021)	Mouse	LPS	Niacin	Pre	Mammary gland score	Mean	SD	3	3.8	0.5	3	1.4	0.9
He S (2021)	Mouse	LPS	NMN	Pre	Tubular injury score	Mean	SD	3	2.8	0.3	3	1.8	0.3
He S (2024)	Mouse	LPS	NMN	D0	LIS	Mean	SD	8	11	2	8	6	2
Imaurouka (v19)	Mouse	LPS	NAM	Post	PAS+ kidney area %	Mean	SEM	5	38	1	5	32	1
Iske (2024)	Mouse	LPS	NAD	Pre	Ileal PMNs	Mean	SD	4	66	20	4	36	16
	Mouse	LPS	NAD	Pre	Kidney PMNs	Mean	SD	5	67	19	5	24	7
	Mouse	LPS	NAD	Pre	Lung PMNs	Mean	SD	5	98	21	5	35	12
	Mouse	LPS	NAD	Pre	Liver PMNs	Mean	SD	5	106	17	5	64	10
Jiang (2022)	Mouse	Virus p36	NAD+	D0	Blocked airway ratio %	Mean	SEM	3	16.6	2.7	4	4.6	1.7
	Mouse	Virus p36	NAD+	D0	Blocked airway area %	Mean	SEM	3	29.6	2.5	4	16.0	2.3
	Mouse	Virus p6	NAD+	D0	Alveolar septal thickness	Mean	SEM	3	0.58	0.02	4	0.53	0.02
Kao (2007)	Rat	LPS	NCA	D0	LIS	Mean	SEM	20	4.98	1.34	20	1.44	1.03
Kwon (2011)	Rat	LPS	Niacin 360	D0	LIS	Median	IQR	14	8.0	7.0, 8.1	14	6.5	5.0, 7.0
	Rat	LPS	Niacin 1180	D0	LIS	Median	IQR	14	8.0	7.0, 8.1	10	4.5	3.0, 5.0

Kwon (2016)	Rat	LPS	Niacin 360	D0	LIS	Mean	SEM	6	11.33	0.33	6	9.17	0.70
	Rat	CLP	Niacin 360	D0	LIS	Mean	SEM	6	8.50	0.64	6	7.17	0.60
Park (2023)	Rat	Bacteria	Niacin	D0	LIS	Median	IQR	6	11.5	10, 14	6	8	7, 11
Tian (2023)	Mouse	LPS	NMN	D0	LIS	Median	IQR	6	3.2	2.8, 3.3	6	2.4	2.0, 2.6
Xu (2014)	Rat	CLP	NAM	Pre	Liver injury score	Mean	SD	6	3.0	0.3	6	4.3	0.2
Zhao (2023)	Mouse	Bacteria	NR 100	D0	LIS	Mean	SD	6	3.2	0.3	6	1.8	0.3
	Mouse	Bacteria	NR 500	D0	LIS	Mean	SD				6	1.3	0.3
	Mouse	Bacteria	NR 1000	D0	LIS	Mean	SD				6	1.0	0.6
	Mouse	Bacteria	NR 100	D0	Heart injury score	Mean	SD	6	3.8	0.3	6	2.4	0.3
	Mouse	Bacteria	NR 500	D0	Heart injury score	Mean	SD				6	2.3	0.3
	Mouse	Bacteria	NR 1000	D0	Heart injury score	Mean	SD				6	1.9	0.3
	Mouse	Bacteria	NR 100	D0	Liver injury score	Mean	SD	6	9.9	3	6	7.5	1
	Mouse	Bacteria	NR 500	D0	Liver injury score	Mean	SD				6	5.1	1
	Mouse	Bacteria	NR 1000	D0	Liver injury score	Mean	SD				6	3.0	1
	Mouse	Bacteria	NR 100	D0	Kidney injury score	Mean	SD	6	13.5	2.5	6	8.5	1.5
	Mouse	Bacteria	NR 500	D0	Kidney injury score	Mean	SD				6	7.0	0.8
	Mouse	Bacteria	NR 1000	D0	Kidney injury score	Mean	SD				6	4.8	2.5

CLP – cecal ligation and puncture; IQR – 25 to 75% quartiles; LIS – lung injury score; LPS – lipopolysaccharide; Rx – treatment; NAD – nicotinamide adenine dinucleotide; N – number of animals; NMN – nicotinamide mononucleotide; NR – nicotinamide riboside; PAS+ – periodic acid-Schiff positive: SD – standard deviation; IQR – 25 to 75% quartiles; SEM – standard error of the mean.

*See SupTable-1 for more detailed information about challenge and treatment regimens and measurement times; **Rx Time – ≥ 1 day before challenge = pre, day of challenge = D0, ≥1 day after challenge = post
