# Supplementary material for: Meta-analysis of niacin and NAD metabolite treatment in infectious disease animal studies suggests benefit but requires confirmation in clinically relevant models
Source: Sci Rep. 2025 Apr 12;15:12621. doi: 10.1038/s41598-025-95735-y (PMC11993703; doi:10.1038/s41598-025-95735-y)
Supplement: Supplementary file 25 — Supplementary Information 25. [file 41598_2025_95735_MOESM25_ESM.pdf]

SupTable-6. Permeability organ injury data*													
Author (year)	Animal	Challenge Type	Rx Type	Initial Rx time**	Parameter	Measure type	Variance type	Control N	Control measure	Control variance	Rx N	Rx measure	Rx variance
Cao (2023)	Mouse	Bacteria	NMN	D0	Evans blue ug/g Lung tissue	Mean	SD	8	8.5	3.0	8	4.5	2.0
	Mouse	Bacteria	NMN	D0	Lung W/D	Mean	SD	8	7.0	1.5	8	5.0	2.0
Fernandes (2011)	Mouse	LPS	NAM	pre	BAL protein g/L	Mean	SEM	9	0.34	0.05	9	0.52	0.12
	Mouse	LPS	NAM	post	BAL protein g/L	Mean	SEM				9	0.22	0.03
Han (2003)	Mouse	LPS	NAD+	D0	FD4 clearance nl/min/cm ileum	Mean	SEM	6	290	30	5	150	10
He, S (2024)	Mouse	LPS	NMN	D0	W/D	Mean	SD	8	6.5	0.5	8	5.0	1.0
Hong (2018)	Mouse	Feces	NR 300	pre	Evans blue ug/g lung/min	Mean	SD	6	16	2	6	12.0	2
	Mouse	Feces	NR 500	pre	Evans blue ug/g lung/min	Mean	SD				6	9	2
	Mouse	LPS	NR 300	pre	Evans blue ug/g lung/min	Mean	SD	5	10	3	5	5	1
Kwon (2016)	Rat	CLP	Niacin	D0	Lung W/D	Mean	SEM	6	5.7	0.2	6	4.7	0.2
Nagai (1994)	Hamster	LPS	Niacin 500	pre	Lung W/D	Mean	SEM	8	4.5	0.1	6	4.2	0.1
	Hamster	LPS	Niacin 250	pre	Lung W/D	Mean	SEM				6	4.0	0.2
	Hamster	LPS	Niacin 500	pre	BAL protein ug/ml	Mean	SEM	8	367	21	6	138	3
	Hamster	LPS	Niacin 250	pre	BAL protein ug/ml	Mean	SEM				6	158	27
Pacl (2023)	Mouse	Bacteria	NAM	Post	Lung wgt	Median	IQR	8	191	(172, 206)	7	132	(122, 141)
	Mouse	Bacteria	NAM	Post	Lung wgt	Median	IQR	7	257	(235, 344)	7	182	(171, 194)
Park (2022)	Mouse	Bacteria	Niacin	D0	Lung wgt	Median	IQR	7	260	(240, 340)	7	180	(175, 185)
Pulido (1999)	Rat	LPS	NAM	D0	Lung W/D	Mean	SE	5	4.76	0.03	5	4.80	0.05
Tian (2023)	Mouse	LPS	NMN	D0	Lung W/D	Mean	SD	6	5.5	0.2	6	4.6	0.2

	Mouse	LPS	NMN	D0	BAL protein ug/mL	Mean	SD	6	260	10	6	140	20
Umpathy (2012)	Mouse	LPS	NAD+	D0	BAL protein mg/mL	Mean	SEM	4	0.82	O.2	4	0.35	0.2
	Mouse	LPS	NAD+	D0	Evans blue ug/g lung tissue	Mean	SEM	4	10	4	4	6	1
Ye (2022)	Mouse	LPS	NAD	D0	Evans blue ug/g lung tissue	Mean	SD	6	51	3.5	6	49.5	3
Yuan (2012)	Mouse	LPS	NAM	D0	Lung W/D	Mean	SD	8	3.7	0.4	8	3.4	0.4

Bac – bacteria; BAL – bronchoalveolar lavage; CLP – cecal ligation and puncture; GAL – D-galactosamine; HPC – hippocampal; IQR – 25 to 75% quartiles; LPS – lipopolysaccharide; N – number of animals; NAD – nicotinamide adenine dinucleotide; NMN – nicotinamide mononucleotide; NR – nicotinamide riboside; Rx – treatment group; SD – standard deviation; SEM – standard error of the mean

*See SupTable-1 for more detailed information about challenge and treatment regimens and measurement times; **Rx Time – ≥ 1 day before challenge = pre, day of challenge = D0, ≥1 day after challenge = post
