# Supplementary material for: Meta-analysis of niacin and NAD metabolite treatment in infectious disease animal studies suggests benefit but requires confirmation in clinically relevant models
Source: Sci Rep. 2025 Apr 12;15:12621. doi: 10.1038/s41598-025-95735-y (PMC11993703; doi:10.1038/s41598-025-95735-y)
Supplement: Supplementary file 26 — Supplementary Information 26. [file 41598_2025_95735_MOESM26_ESM.pdf]

SupTable-7. Chemical organ injury data*													
Author (year)	Animal Type	Challenge Type	Rx Type	Initial Rx Time**	Parameter	Measure type	Variance type	Control N	Control measure	Control variance	Rx N	Rx measure	Rx variance
Cao (2023)	Mouse	Bac	NMN	D0	ALT (IU/L)	Mean	SD	8	22.5	2.5	8	11.0	5.0
	Mouse	Bac	NMN	D0	AST (IU/L)	Mean	SD	8	10	3	8	4	3
	Mouse	Bac	NMN	D0	Cre (mg/L)	Mean	SD	8	6.2	1.2	8	3.0	0.5
	Mouse	Bac	NMN	D0	BUN (mg/L)	Mean	SD	8	17	2	8	12	1
He, S (2021)	Mouse	LPS	NMN	Pre	BUN (mg/dL)	Mean	SD	3	140	10	3	50	10
	Mouse	LPS	NMN	Pre	Cre (mg/dL)	Mean	SD	3	1.2	0.1	3	0.5	0.1
Imaurouka (2019)	Mouse	LPS	NAM	Pre	Cre (mg/dL)	Mean	SEM	5	45	15	5	20	10
Kao (2007)	Rat	LPS	NAM	D0	BUN (mg/dL)	Mean	SEM	10	39	2	10	18	1
	Rat	LPS	NAM	D0	Cre (mg/dL)	Mean	SEM	10	0.33	0.03	10	0.20	0.05
	Rat	LPS	NAM	D0	AST (SGOT) (u/L)	Mean	SEM	10	389	20	10	300	10
	Rat	LPS	NAM	D0	ALT (SGPT) (u/L)	Mean	SEM	10	250	15	10	180	15
Kwon (2016)	Rat	Bac	Niacin	D0	BUN (mg/dL)	Median	IQR	6	19.0	15.1, 21.5	6	18.1	12.2, 23.8
	Rat	Bac	Niacin	D0	Cre (mg/dL)	Median	IQR	6	0.49	0.42, 0.59	6	0.56	0.41, 0.67
	Rat	Bac	Niacin	D0	Bilirubin (mg/dL)	Median	IQR	6	0.09	0.06, 0.12	6	0.04	0.03, 0.05
	Rat	Bac	Niacin	D0	AST (mg/dL)	Median	IQR	6	277	213, 346	6	364	219, 414
	Rat	Bac	Niacin	D0	ALT (mg/dL)	Median	IQR	6	74	57, 95	6	73	34, 76
Scharte (2003)	Sheep	Bac	NAM	D0	Bilirubin (Umol/L)	Mean	SEM	6	0.68	0.13	6	1.03	0.38
	Sheep	Bac	NAM	D0	AST (U/L)	Mean	SEM	6	93.8	9.0	6	103.7	11.5
	Sheep	Bac	NAM	D0	ALT (U/L)	Mean	SEM	6	20.7	1.8	6	21.8	2.5
Wray (1998)	Rat	LPS	NAM	D0	BUN (mmole/L)	Mean	SEM	17	18	1	17	19	1
	Rat	LPS	NAM	D0	Cre (mmole/L)	Mean	SEM	17	56	10	17	53	10
	Rat	LPS	NAM	D0	Bilirubin (mmole/L)	Mean	SEM	17	11	3	17	3	1
	Rat	LPS	NAM	D0	ALT (U/L)	Mean	SEM	17	600	150	17	700	300
	Rat	LPS	NAM	D0	AST (U/L)	Mean	SEM	17	1000	200	17	1300	400
Xu (2014)	Rat	Bac	NAM	Pre	ALT (U/L)	Mean	SD	6	320	10	6	440	20
	Rat	Bac	NAM	Pre	AST (U/L)	Mean	SD	6	400	10	6	500	20
Yuan (2012)	Mouse	LPS/GAL	NAM	Pre	ALT (U/L)	Mean	SD	8	2300	300	8	700	100
	Mouse	LPS/GAL	NAM	Pre	AST (U/L)	Mean	SD	8	1300	200	8	600	200
	Mouse	LPS	NAM	Pre	ALT (U/L)	Mean	SD	8	160	35	8	80	20
	Mouse	LPS	NAM	Pre	BUN (mmol/L)	Mean	SD	8	3.3	0.7	8	2.3	0.5

ALT and AST – alanine and aspartate aminotransferase; BUN – blood urea nitrogen; Cre – creatinine; GAL – D-galactosamine; IQR – 25 and 75% quartiles; LPS – lipopolysaccharide; Rx – treatment; N – number of animals; NAD – nicotinamide adenine dinucleotide; NMN – nicotinamide mononucleotide; SD – standard deviation; SEM – standard error of the mean; SGOT – serum glutamic oxaloacetic transaminase; SGPT – Serum glutamic pyruvic transaminase

*See SupTable-1 for more detailed information about challenge and treatment regimens and measurement times; **Rx Time – ≥ 1 day before challenge = pre, day of challenge = D0, ≥1 day after challenge = post
