# Supplementary material for: Meta-analysis of niacin and NAD metabolite treatment in infectious disease animal studies suggests benefit but requires confirmation in clinically relevant models
Source: Sci Rep. 2025 Apr 12;15:12621. doi: 10.1038/s41598-025-95735-y (PMC11993703; doi:10.1038/s41598-025-95735-y)
Supplement: Supplementary file 27 — Supplementary Information 27. [file 41598_2025_95735_MOESM27_ESM.pdf]

SupTable-8. Tumor necrosis factor-α data*

Author (year)	Animal Type	Challenge Type	Rx Type	Initial Rx Time**	Parameter	Measure type	Variance type	Control N	Control measure	Control variance	Rx N	Rx measure	Rx variance
Abdel (2023)	Mouse	LPS	Niacin	D0	HPC TNFα pg/mg	Mean	SD	6	70	5	6	28	7
Cao (2023)	Mouse	Bacteria	NMN	D0	Serum TNFα pg/ml	Mean	SD	8	65	50	8	10	10
Doganany (2022)	Rat	CLP	NAD 100	Pre	Liver TNFα % + cells	Mean	SD	7	55.7	7.4	7	25.6	1.3
	Rat	CLP	NAD 300	Pre	Liver TNFα % + cells	Mean	SD				7	20.0	1.4
	Rat	CLP	NAD 100	Pre	Kidney TNFα % + cells	Mean	SD	7	62.2	2.0	7	30.7	3.9
	Rat	CLP	NAD 300	Pre	Kidney TNFa % + cells	Mean	SD				7	21.7	2.9
Fukuzawa (1997)	Mouse	LPS	NAM	D0	Blood TNFα pg/ml	Mean	SE	7	180	10	7	100	10
	Mouse	LPS	NAM	D0	Blood TNFα% of cont	Mean	SE	7	-	-	7	75	15
	Mouse	LPS	NAM	D0	Blood TNFα% of cont	Mean	SE				7	45	1
	Mouse	LPS	NAM	D0	Blood TNFα% of cont	Mean	SE				7	40	5
Guo (2020)	Cow	Mastitis	Niacin	D0	Blood TNFα, ng/L	Mean	SD	6	240	20	6	150	25
	Cow	Mastitis	Niacin	D0	Milk TNFα, ng/L	Mean	SD	6	140	20	6	60	20
Guo (2021)	Mouse	LPS	Niacin	Pre	M-Gland TNFα mRNA foldΔ	Mean	SD	5	2	1	5	0.6	0.5
	Mouse	LPS	Niacin	Pre	TNFα pg/mg mam gland	Mean	SD	5	0.7	0.3	5	0.2	0.2
Imaurouka (2019)	Mouse	LPS	NAM	Post	Renal TNFα mRNA#	Mean	SEM	6	1	0.05	6	0.7	0.05
Iske (2024)	Mouse	LPS	NAD	Pre	Serum TNFα pg/ml	Mean	SD	6	1241	218	6	592	139
Kao (2007)	Rat	LPS	NCA	D0	Plasma TNFα pg/ml	Mean	SEM	10	2500	200	10	250	100
Kwon (2011)	Rat	LPS	Niacin 360	D0	Lung TNFα mRNA (x nonLPS)	Median	IQR	6	10.7	10, 11.5	6	8.7	8, 10.1
	Rat	LPS	Niacin 1180	D0	Lung TNFα mRNA (x nonLPS)	Median	IQR	6			6	7	6.2, 8.5
	Rat	LPS	Niacin 360	D0	Serum TNFα (pg/mL)	Median	IQR	6	220	215, 225	6	190	175, 200

	Rat	LPS	Niacin 1180	D0	Serum TNFα (pg/mL)	Median	IQR	6			6	130	110, 150
Kwon (2016)	rat	LPS	Niacin	D0	Lung TNFα mRNA (x nonLPS)	Median	IQR	6	14	9.5, 16.1	6	9.0	8.5, 9.5
LeClaire ('96)	Mouse	SEB + LPS	Niacin	D0	Serum TNFα%maxresponse	Mean	SEM	3	100	2	3	31	17
Li HR (2023)	Mouse	Bacteria	NMN	D0	Plasma TNFα pg/ml	Mean	SD	6	850	75	6	500	150
Li HR (2023)	Mouse	Bacteria	NMN	D0	HPC TNFα pg/mg	Mean	SD	6	120	20	6	50	10
Li HR (2023)	Mouse	Bacteria	NMN	D0	HPC TNFα pg/mg	Mean	SD	6	80	10	6	55	20
Li HR (2023)	Mouse	Bacteria	NMN	D0	Plasma TNFα pg/ml	Mean	SD	6	800	150	6	575	75
Park (2023)	Rat	Bacteria	Niacin	D0	Lung TNFα mRNA fold change	Median	IQR	6	5.0	(3.5, 7.5)	6	2.5	(2.2, 4.8)
Roboon (2021)	Mouse	LPS	NR	Pre	HPC TNFα relative mRNA	Mean	SEM	6	459	120	6	130	25
	Mouse	LPS	NR	Pre	HPC TNFα relative mRNA	Mean	SEM	5	392	22	5	191	33
	Mouse	LPS	NR	D0	HPC TNFα relative mRNA	Mean	SEM	4	515	72	4	408	47
Tian (2023)	Mouse	LPS	NMN	D0	BAL TNFα pg/ml	Mean	SD	6	310	50	6	190	20
Umapathy (2012)	Mouse	LPS	NAD+	D0	Lung TNFα RNA foldΔ	Mean	SEM	4	49	0.1	4	32	0.1
Xu (2014)	Rat	CLP	NAM	Pre	Serum TNFα pg/ml	Mean	SD	6	290	10	6	320	20
Ye (2022)	Mouse	Bacteria	NAD	D0	Serum TNF pg/ml	Mean	SD	6	97	13	6	94	11
Yuan (2012)	Mouse	LPS+Gal	NAM	D0	Blood TNFα pg/mL	Mean	SD	8	530	120	8	270	25
	Mouse	LPS	NAM	D0	Blood TNFα pg/mL	Mean	SD	8	310	50	8	210	50
Zhao (2023)	Mouse	Bacteria	NR 100	D0	Plasma TNFα pg/ml	Mean	SD	6	245	50	6	225	30
Zhao (2023)	Mouse	Bacteria	NR 500	D0	Plasma TNFα pg/ml	Mean	SD	6	245	50	6	190	50
Zhao (2023)	Mouse	Bacteria	NR 1000	D0	Plasma TNFα pg/ml	Mean	SD	6	245	50	6	150	35

BAL – bronchoalveolar lavage; CLP – cecal ligation and puncture; GAL – D-galactosamine; HPC – hippocampal; IQR – 25 to 75% quartiles; LPS – lipopolysaccharide; N – number of animals; NAD – nicotinamide adenine dinucleotide; NMN – nicotinamide mononucleotide; NR – nicotinamide riboside; Rx – treatment group; SD – standard deviation; SEM – standard error of the mean

*See SupTable-1 for more detailed information about challenge and treatment regimens and measurement times; **Rx Time – ≥ 1 day before challenge = pre, day of challenge = D0, ≥1 day after challenge = post
