# Supplementary material for: Meta-analysis of niacin and NAD metabolite treatment in infectious disease animal studies suggests benefit but requires confirmation in clinically relevant models
Source: Sci Rep. 2025 Apr 12;15:12621. doi: 10.1038/s41598-025-95735-y (PMC11993703; doi:10.1038/s41598-025-95735-y)
Supplement: Supplementary file 28 — Supplementary Information 28. [file 41598_2025_95735_MOESM28_ESM.pdf]

SupTable-9. Interleukin-6 data*													
Author (year)	Animal Type	Challenge Type	Rx Type	Initial Rx Time **	Parameter	Measure type	Variance type	Control N	Control measure	Control variance	Rx N	Rx measure	Rx variance
Doganany (2022)	Rat	CLP	NAD 100	Pre	Liver IL-6 % + cells	Mean	SD	7	53.9	4.2	7	25.1	1.8
	Rat	CLP	NAD 300	Pre	Liver IL-6 % + cells	Mean	SD				7	15.3	1.6
	Rat	CLP	NAD 100	Pre	Kidney IL-6 % + cells	Mean	SD	7	74.4	2.1	7	31.0	2.7
	Rat	CLP	NAD 300	Pre	Kidney IL-6 % + cells	Mean	SD				7	24.6	3.1
Guo (2020)	Cow	Mastitis	Niacin	D0	Blood IL-6, ng/L	Mean	SD	6	42	10	6	30	5
	Cow	Mastitis	Niacin	D0	Milk IL-6, ng/L	Mean	SD	6	26	3	6	14	3
Guo (2021)	Mouse	LPS	Niacin	Pre	M-Gland IL-6 mRNA foldΔ	Mean	SD	5	3.4	1.2	5	0.5	0.4
	Mouse	LPS	Niacin	Pre	IL-6 pg/mg mam gland	Mean	SD	5	1.9	0.9	5	1.1	0.3
Imaurouka (2019)	Mouse	LPS	NAM	Post	Renal IL-6 mRNA#	Mean	SEM	6	1.0	0.3	6	0.5	0.1
Iske (2024)	Mouse	LPS	NAD	Pre	Serum IL-6 pg/ml	Mean	SD	6	62	13	6	29	9
Kwon (2011)	Rat	LPS	Niacin 360	D0	Lung IL-6 mRNA (xnonLPS)	Median	IQR	6	50	40, 60	6	39	35, 44
	Rat	LPS	Niacin 1180	D0	Lung IL-6 mRNA (xnonLPS)	Median	IQR				6	27	23, 29
	Rat	LPS	Niacin 360	D0	Serum IL-6 (pg/mL)	Median	IQR	6	4,750	4000, 6750	6	3800	2500, 4100
	Rat	LPS	Niacin 1180	D0	Serum IL-6 (pg/mL)	Median	IQR				6	1900	1600, 2500
Kwon (2016)	rat	LPS	Niacin	D0	Lung IL-6 mRNA (xnonLPS)	Median	IQR	6	390	190, 550	6	190	180, 200
Li HR (2023)	Mouse	Bacteria	NMN	D0	Plasma IL-6 pg/ml	Mean	SD	6	1500	125	6	750	50
	Mouse	Bacteria	NMN	D0	HPC IL-6 pg/mg	Mean	SD	6	50	7.5	6	22	5
	Mouse	Bacteria	NMN	D0	HPC IL-6 pg/mg	Mean	SD	6	37.5	15	6	18	7.5
	Mouse	Bacteria	NMN	D0	Plasma IL-6 pg/ml	Mean	SD	6	1550	125	6	850	70
Liu (2024)	Mouse	Bacteria	NMN	Pre	Serum IL-6 pg/ml	Mean	SE	5	315	5	5	85	5
	Mouse	Bacteria	NMN	Pre	Peritoneal Mac IL-6 relative mRNA	Mean	SE	5	225	2	5	80	2

Park (2023)	Rat	Bacteria	Niacin	D0	Lung IL-6 mRNA fold change	Median	IQR	6	45	(33, 75)	6	20	(19, 38)
Roboon (2021)	Mouse	LPS	NR	Pre	IL-6 relative mRNA	Mean	SEM	6	30.9	3.7	6	13.2	3.5
	Mouse	LPS	NR	Pre	IL-6 relative mRNA	Mean	SEM	5	45.1	6.3	5	17.4	4.5
	Mouse	LPS	NR	D0	IL-6 relative mRNA	Mean	SEM	4	32.9	4.2	4	27.6	1.6
Shi (2017)	Mouse	LPS	Niacin	D0	Ascites IL-6, OD450	Mean	SD	9	5.3±0.2	0.2	9	3.5	0.2
Tian (2023)	Mouse	LPS	NMN	D0	BAL IL-6 pg/ml	Mean	SD	6	340	10	6	170	25
Xu (2014)	Rat	CLP	NAM	Pre	Serum IL-6 pg/ml	Mean	SD	6	290	5	6	330	20
Ye (2022)	Mouse	Bacteria	NAD	D0	Serum IL-6 pg/ml	Mean	SD	6	446	32	6	405	41
Yuan (2012)	Mouse	LPS	NAM	D0	Blood IL-6 pg/mL	Mean	SD	8	110	30	8	70	30
Zhao (2023)	Mouse	Bacteria	NR 100	D0	Plasma IL-6 pg/ml	Mean	SD	6	390	100	6	415	40
	Mouse	Bacteria	NR 500	D0	Plasma IL-6 pg/ml	Mean	SD	6	390	100	6	280	30
	Mouse	Bacteria	NR 1000	D0	Plasma IL-6 pg/ml	Mean	SD	6	390	100	6	290	40

BAL – bronchoalveolar lavage; CLP – cecal ligation and puncture; GAL – D-galactosamine; HPC – hippocampal; ; IQR – 25 to 75% quartiles; LPS – lipopolysaccharide; mam – mammary gland; N – number of animals; NAD – nicotinamide adenine dinucleotide; NMN – nicotinamide mononucleotide; NR – nicotinamide riboside; Rx – treatment group; SD – standard deviation; SEM – standard error of the mean

*See SupTable-1 for more detailed information about challenge and treatment regimens and measurement times; **Rx Time – ≥ 1 day before challenge = pre, day of challenge = D0, ≥1 day after challenge = post
