# Supplementary material for: Meta-analysis of niacin and NAD metabolite treatment in infectious disease animal studies suggests benefit but requires confirmation in clinically relevant models
Source: Sci Rep. 2025 Apr 12;15:12621. doi: 10.1038/s41598-025-95735-y (PMC11993703; doi:10.1038/s41598-025-95735-y)
Supplement: Supplementary file 29 — Supplementary Information 29. [file 41598_2025_95735_MOESM29_ESM.pdf]

SupTable – 10. Interleukin-1β data*

Author (year)	Animal Type	Challenge Type	Rx Type	Initial Rx Time**	Parameter	Measure type	Variance type	Control N	Control measure	Control variance	Rx N	Rx measure	Rx variance
Abdel (2023)	Mouse	LPS	NA	D0	HPC IL-1β pg/mg	Mean	SD	6	120	5	6	60	8
Cao (2023)	Mouse	Bacteria	NMN	D0	Serum IL-1β pg/ml	Mean	SD	8	50	40	8	20	20
Doganany (2022)	Rat	CLP	NAD 100	Pre	Liver IL-1β % + cells	Mean	SD	7	59.9	4.7	7	32.1	3.8
	Rat	CLP	NAD 300	Pre	Liver IL-1β % + cells	Mean	SD	7				24.0	1.6
	Rat	CLP	NAD 100	Pre	Kidney IL-1β % + cells	Mean	SD	7	63.4	7.3	7	30.6	2.8
	Rat	CLP	NAD 300	Pre	Kidney IL-1β % + cells	Mean	SD	7				22.3	2.1
Guo (2020)	Cow	Mastitis	Niacin	D0	Blood IL-1β, ng/L	Mean	SD	6	35	5	6	15	5
	Cow	Mastitis	Niacin	D0	Milk IL-1β, ng/L	Mean	SD	6	19	4	6	8	2
Guo (2021)	Mouse	LPS	Niacin	Pre	IL-1β pg/mg mam gland	Mean	SD	5	2.8	0.2	5	1	0.1
Iske (2024)	Mouse	LPS	NAD	Pre	Serum IL-1β pg/ml	Mean	SD	6	4528	491	6	840	165
Kao (2007)	Rat	LPS	Niacin	D0	Plasma IL-1β pg/ml	Mean	SEM	10	1500	20	10	100	50
Li HR (2023)	Mouse	Bacteria	NMN	D0	Plasma IL-1β pg/ml	Mean	SD	6	230	45	6	130	40
	Mouse	Bacteria	NMN	D0	Hippo IL-1β pg/mg	Mean	SD	6	33	4.5	6	13	5
	Mouse	Bacteria	NMN	D0	Hippo IL-1β pg/mg	Mean	SD	6	28	4.5	6	14.5	5
	Mouse	Bacteria	NMN	D0	Plasma IL-1β pg/ml	Mean	SD	6	245	50	6	160	25
Liu (2024)	Mouse	Bacteria	NMN	Pre	Serum IL-1β pg/ml	Mean	SEM	5	225	2	5	100	2
	Mouse	Bacteria	NMN	Pre	Peritoneal Mac IL-1β relative mRNA	Mean	SEM	5	100	2	5	70	2
Roboon (2021)	Mouse	LPS	NR	Pre	IL-1β relative mRNA	Mean	SEM	6	133.5	26.4	6	41.4	11.6
	Mouse	LPS	NR	Pre	IL-1β relative mRNA	Mean	SEM	5	133.5	19.2	5	53.9	8.1
	Mouse	LPS	NR	D0	IL-1β relative mRNA	Mean	SEM	4	67.3	8.1	4	59.7	6.6
Tian (2023)	Mouse	LPS	NMN	D0	BAL IL-1β pg/ml	Mean	SD	6	58	7	6	30	3
Umapathy (2012)	Mouse	LPS	NAD+	D0	Lung IL-1β RNA foldΔ	Mean	SEM	4	35	0.1	4	20	0.5

BAL – bronchoalveolar lavage; CLP – cecal ligation and puncture; GAL – D-galactosamine; HPC – hippocampal; ; IQR – 25 to 75% quartiles; LPS – lipopolysaccharide; mam – mammary gland; N – number of animals; NAD – nicotinamide adenine dinucleotide; NMN – nicotinamide mononucleotide; NR – nicotinamide riboside; Rx – treatment group; SD – standard deviation; SEM – standard error of the mean

*See SupTable-1 for more detailed information about challenge and treatment regimens and measurement times; **Rx Time – ≥ 1 day before challenge = pre, day of challenge = D0, ≥1 day after challenge = post
