# Supplementary material for: Meta-analysis of niacin and NAD metabolite treatment in infectious disease animal studies suggests benefit but requires confirmation in clinically relevant models
Source: Sci Rep. 2025 Apr 12;15:12621. doi: 10.1038/s41598-025-95735-y (PMC11993703; doi:10.1038/s41598-025-95735-y)
Supplement: Supplementary file 30 — Supplementary Information 30. [file 41598_2025_95735_MOESM30_ESM.pdf]

SupTable-11. Catalase (CAT), glutathione (GSH), and superoxide dismutase (SOD) antioxidant data*

Author (year)	Animal Type	Challenge Type	Rx Type	Initial Rx Time**	Parameter	Measure type	Variance type	Control N	Control measure	Control variance	Rx N	Rx measure	Rx variance
Doganay (2022)	Rat	Bacteria	NAD 100	Pre	Serum GSH nM/mg	Mean	SD	7	40,000	6,000	7	48,000	6,000
	Rat	Bacteria	NAD 300	Pre	Serum GSH nM/mg	Mean	SD	7	40,000	6,000	7	61,000	6,000
	Rat	Bacteria	NAD 100	Pre	Kidney GSH nM/mg	Mean	SD	7	0.90	0.40	7	1.1	0.4
	Rat	Bacteria	NAD 300	Pre	Kidney GSH nM/mg	Mean	SD	7	0.90	0.40	7	1.2	0.4
	Rat	Bacteria	NAD 100	Pre	Liver GSH nM/mg	Mean	SD	7	1.0	0.05	7	1	0.05
	Rat	Bacteria	NAD 300	Pre	Liver GSH nM/mg	Mean	SD	7	1.0	0.05	7	1.1	0.05
	Rat	Bacteria	NAD 100	Pre	Serum CAT nM/mg	Mean	SD	7	390,000	10,000	7	400,000	10,000
	Rat	Bacteria	NAD 300	Pre	Serum CAT nM/mg	Mean	SD	7	390,000	10,000	7	430,000	10,000
	Rat	Bacteria	NAD 100	Pre	Kidney CAT nM/mg	Mean	SD	7	0.8	1.0	7	7.3	1.0
	Rat	Bacteria	NAD 300	Pre	Kidney CAT nM/mg	Mean	SD	7	0.8	1.0	7	7.5	1.0
	Rat	Bacteria	NAD 100	Pre	Liver CAT nM/mg	Mean	SD	7	1.2	0.6	7	8.0	0.6
	Rat	Bacteria	NAD 300	Pre	Liver CAT nM/mg	Mean	SD	7	1.2	0.6	7	9.5	0.6
Du (2022)	Mouse	LPS	NMN	Pre	Lung MnSOD % vs control	Mean	SD	15	2.0	0.5	18	2.8	0.4
	Mouse	LPS	NMN	Pre	Lung MnSOD % vs control	Mean	SD	5	2.0	0.3	5	2.8	0.5
Kwon (2011)	Rat	LPS	Niacin 390	D0	Lung GSH pmol/mg tissue	Median	IQR	14	400	(390, 405)	14	510	(505, 530)

	Rat	LPS	Niacin 1180	D0	Lung GSH pmol/mg tissue	Median	IQR	14	400	(390, 405)	10	570	(560, 580)
Kwon (2016)	Rat	LPS	Niacin	Do	Lung GSH/GSSG	Median	IQR	6	1.5	(1.4, 1.6)	6	1.7	(1.6, 1.8)
Li, HR (2023)	Mouse	Bacteria	NMN	D0	HPC SOD U/mg protein	Mean	SD	6	70	15	6	125	20
	Mouse	Bacteria	NMN	D0	HPC SOD U/mg protein	Mean	SD	6	100	3	6	160	8
Park (2023)	Rat	Bacteria	Niacin	D0	Lung GSH Pmol/mg protein	Median	IQR	6	230	(190, 240)	6	260	(250, 280)
Selli (2023)	Rat	Bacteria	NR	D0	SOD Activity ovary U/mg tissue	Mean	SEM	8	13.5	2.5	8	27	1.5
	Rat	Bacteria	NR	D0	CAT Activity ovary U/mg tissue	Mean	SEM	8	15	1.5	8	25	1.5
	Rat	Bacteria	NR	D0	GSH ovary nmol/mg tissue	Mean	SEM	8	3.5	1.5	8	8.5	2.5
Tian (2023)	Mouse	LPS	NMN	UC	Lung SOD U/mg tissue	Median	IQR	6	240	(235, 245)	6	320	(300, 330)

HPC – hippocampal; IQR – 25 to 75% quartiles; LPS – lipopolysaccharide; N – number of animals; NAD – nicotinamide adenine dinucleotide; NMN – nicotinamide mononucleotide; NR – nicotinamide riboside; Rx – treatment group; SD – standard deviation; SEM – standard error of the mean; UC - unclear

*See SupTable-1 for more detailed information about challenge and treatment regimens and measurement times; **Initial Rx Time – ≥ 1 day before challenge = pre, day of challenge = D0, ≥1 day after challenge = post
