# Supplementary material for: Meta-analysis of niacin and NAD metabolite treatment in infectious disease animal studies suggests benefit but requires confirmation in clinically relevant models
Source: Sci Rep. 2025 Apr 12;15:12621. doi: 10.1038/s41598-025-95735-y (PMC11993703; doi:10.1038/s41598-025-95735-y)
Supplement: Supplementary file 31 — Supplementary Information 31. [file 41598_2025_95735_MOESM31_ESM.pdf]

SubTable-12. Malondialdehyde (MDA), protein carbonyl and reactive oxygen species (ROS) oxidation marker data*													
Author (year)	Animal Type	Challenge Type	Rx Type	Initial Rx Time**	Parameter	Measure type	Variance type	Control N	Control measure	Control variance	Rx N	Rx measure	Rx variance
Cao (2023)	Mouse	Bacteria	NMN	D0	Heart ROS Nmol/min	Mean	SD	8	4	0.1	8	3.0	0.2
	Mouse	Bacteria	NMN	D0	Heart MDA Umol/L	Mean	SD	8	3.2	0.8	8	1.8	0.2
	Mouse	Bacteria	NMN	D0	Lung ROS Nmol/min	Mean	SD	8	55	2.0	8	35	3.0
	Mouse	Bacteria	NMN	D0	Lung MDA Umol/L tissue	Mean	SD	8	2.6	0.8	8	1.6	0.3
	Mouse	Bacteria	NMN	D0	Liver MDA Umol/L	Mean	SD	8	2.6	0.4	8	1.2	0.1
	Mouse	Bacteria	NMN	D0	Liver protein carbonyl fold change	Mean	SD	8	3.2	0.8	8	2.0	0.6
	Mouse	Bacteria	NMN	D0	Kidney MDA Umol/L	Mean	SD	8	3.0	0.1	8	1.8	0.4
	Mouse	Bacteria	NMN	D0	Kidney protein carbonyl fold change	Mean	SD	8	22.4	1.3	8	1.6	0.3
Du (2022)	Mouse	LPS	NMN	Pre	Lung ROS % vs control	Mean	SD	15	225	24	18	150	10
Hong (2018)	Mouse	Bacteria	NR 300	D0	Lung MDA umol/mg protein	Mean	SD	5	1900	400	5	600	100
	Mouse	Bacteria	NR 500	D0	Lung MDA umol/mg protein	Mean	SD	5	1900	400	5	50	100
	Mouse	Bacteria	NR 500	D0	Lung protein carbonyl fold change %	Mean	SD	5	1.4	0.05	5	1.0	0.2
	Mouse	LPS	NR 300	D0	Lung MDA umol/mg protein	Mean	SD	5	1800	100	5	800	50
	Mouse	Bacteria	NR 500	D0	Heart MDA umol/mg protein	Mean	SD	7	17	2	7	13	2

	Mouse	Bacteria	NR 500	D0	Lung protein carbonyl fold change %	Mean	SD	7	1.4	0.5	7	1.0	0.2
Kwon (2011)	Rat	LPS	Niacin 390	D0	Lung MDA pmol/mg tissue	Median	IQR	14	250	(230, 260)	14	170	(120, 160)
	Rat	LPS	Niacin 1180	D0	Lung MDA pmol/mg tissue	Median	IQR	14	250	(230, 260)	10	140	(130, 160)
Kwon (2016)	Rat	LPS	Niacin	Do	Lung MDA pmol/mg tissue	Median	IQR	6	170	(160, 180)	6	150	(140, 160)
Li, HR (2023)	Mouse	Bacteria	NMN	D0	HPC MDA Nmol/mg protein	Mean	SD	6	20	5	6	12	1.5
	Mouse	Bacteria	NMN	D0	HPC ROS % area stained	Mean	SD	6	27.5	3.0	6	10.0	2.5
	Mouse	Bacteria	NMN	D0	HPC MDA Nmol/mg protein	Mean	SD	6	12.5	3.0	6	9.0	2.5
Park (2023)	Rat	Bacteria	Niacin	D0	Lung MDA Pmol/mg protein	Median	IQR	6	103	(98, 110)	6	91	(85, 98)
Selli (2023)	Rat	Bacteria	NR	D0	MDA ovary nmol/mg tissue	Mean	SEM	8	27	3	8	20	1.5
Tian (2023)	Mouse	LPS	NMN	UC	Lung MDA nM/mg tissue	Median	IQR	6	2.1	(2.0, 2.4)	6	1.4	(1.2, 1.6)
Xu (2014)	Rat	Bacteria	NAM	Pre	Liver MDA Nmol/g	Mean	SD	6	240	10	6	260	5

HPC – hippocampal; IQR – 25 to 75% quartiles; LPS – lipopolysaccharide; N – number of animals; NAD – nicotinamide adenine dinucleotide; NMN – nicotinamide mononucleotide; NR – nicotinamide riboside; Rx – treatment group; SD – standard deviation; SEM – standard error of the mean; UC - unclear

*See SupTable-1 for more detailed information about challenge and treatment regimens and measurement times; **Initial Rx Time – ≥ 1 day before challenge = pre, day of challenge = D0, ≥1 day after challenge = post
