# Supplementary material for: Meta-analysis of niacin and NAD metabolite treatment in infectious disease animal studies suggests benefit but requires confirmation in clinically relevant models
Source: Sci Rep. 2025 Apr 12;15:12621. doi: 10.1038/s41598-025-95735-y (PMC11993703; doi:10.1038/s41598-025-95735-y)
Supplement: Supplementary file 32 — Supplementary Information 32. [file 41598_2025_95735_MOESM32_ESM.pdf]

SupTable-13. Myeloperoxidase (MPO) data*													
Author (year)	Animal Type	Challenge Type	Rx Type	Initial Rx Time**	Parameter	Measure type	Variance type	Control N	Control measure	Control variance	Rx N	Rx measure	Rx variance
Bettenworth (2014)	Mouse	Bacteria	NAM	Pre	Colonic MPO u/mg protein	Mean	SEM	9	1822	249	9	752	189
Cao (2023)	Mouse	Bacteria	NMN	D0	Heart MPO U/g tissue	Mean	SD	8	17	3	8	11	4
	Mouse	Bacteria	NMN	D0	Lung MPO U/g tissue	Mean	SD	8	22	8	8	15	8
	Mouse	Bacteria	NMN	D0	Liver MPO U/g tissue	Mean	SD	8	40	5	8	25	5
	Mouse	Bacteria	NMN	D0	Kidney MPO U/g tissue	Mean	SD	8	24	5	8	18	2
Guo (2021)	Mouse	LPS	Niacin	Pre	Mammary Gland MPO, OD	Mean	SD	3	6.9	1.1	3	2.9	0.6
He S (2024)	Mouse	LPS	NMN	D0	Lung MPO U/g tissue	Mean	SD	8	11.0	2.0	8	8.0	0.5
Hong (2018)	Mouse	Bacteria	NR 300	D0	Lung MPO activity U/g tissue	Mean	SD	5	15	3	5	12	3
	Mouse	Bacteria	NR 500	D0	Lung MPO activity U/g tissue	Mean	SD	5	15	3	5	9	2
	Mouse	LPS	NR 300	D0	Lung MPO activity U/g tissue	Mean	SD	5	15	3	5	9	0.5
	Mouse	Bacteria	NR 500	D0	Lung MPO activity U/g tissue	Mean	SD	7	13	1	7	9	1
Kwon (2016)	Rat	LPS	Niacin	Do	Lung MPO mU/mg protein	Median	IQR	6	7.5	(5.5, 9.5)	6	5.5	(4.0, 6.5)
Park (2023)	Rat	Bacteria	Niacin	D0	Lung MPO mU/mg protein	Median	IQR	6	20	(19.5, 23)	6	17	(12, 19)
Pulido (1999)	Rat	LPS	NAM	D0	Lung MPO u/mg tissue	Mean	SEM	5	0.75	0.07	5	0.65	0.10
Selli (2023)	Rat	Bacteria	NR	D0	MPO Activity ovary U/mg tissue	Mean	SEM	8	84	3	8	60	2.5

Umapathy (2012)	Mouse	LPS	NAD	D0	Lung MPO activity u/mg/min	Mean	SEM	4	65	5	4	38	2

IQR – 25 to 75% quartiles; LPS – lipopolysaccharide; N – number of animals; NAD – nicotinamide adenine dinucleotide; NMN – nicotinamide mononucleotide; NR – nicotinamide riboside; Rx – treatment group; SD – standard deviation; SEM – standard error of the mean

*See SupTable-1 for more detailed information about challenge and treatment regimens and measurement times; **Initial Rx Time – ≥ 1 day before challenge = pre, day of challenge = D0, ≥1 day after challenge = post
