# Supplementary material for: Meta-analysis of niacin and NAD metabolite treatment in infectious disease animal studies suggests benefit but requires confirmation in clinically relevant models
Source: Sci Rep. 2025 Apr 12;15:12621. doi: 10.1038/s41598-025-95735-y (PMC11993703; doi:10.1038/s41598-025-95735-y)
Supplement: Supplementary file 33 — Supplementary Information 33. [file 41598_2025_95735_MOESM33_ESM.pdf]

SupTable-14. Quality of evidence						
	Sample size or power calculation described	Randomization of animals to receive treatment described	Blinding of treatment administration described	Blinding of survival outcome assessment described*	Blinding of histology measures described	Blinding of nonsurvival, nonhistologic measures described
Abdel ('23)	no	yes	no	na	no	no
Bettenworth ('14)	no	no	no	na	yes	no
Cao ('23)	no	no	no	no	na	na
Chang ('54)	no	no	no	no	na	no
Cros ('22)	no	yes	no	no	na	no
Doganany ('22)	no	yes	no	na	na	no
Du ('22)	no	yes	no	no	yes	no
Duan (2023)	no	yes	no	no	na	na
Fernandez ('10)	no	no	no	na	na	no
Fukuzawa ('96)	no	no	no	na	na	no
Fulton ('74)	no	no	no	no	na	na
Griesman ('79)	no	yes	no	no	na	na
Guo ('20)	no	no	no	na	na	no
Guo ('21)	no	no	no	na	yes	no
Han ('03)	no	no	no	na	na	na
He, D. ('23)	no	no	no	no	na	no
He, M. ('16)	no	no	no	no	na	no
He, S. ('22)	no	no	no	na	yes	no
He, S. ('24)	no	yes	no	na	Yes	no
Hilton ('76)	no	no	no	no	na	no
Hong ('18)	no	no	no	no	na	no
Imarouka ('19)	no	no	no	na	no	no
Iske ('24)	no	yes	no	no	no	no
Izadoanah ('23)	no	no	no	na	na	no
Jiang ('22)	no	no	no	no	no	no
Kao ('07)	no	no	no	na	yes	no
Kwon ('11)	no	no	no	no	yes	no
Kwon ('16)	yes	yes	no	no	yes	no
LeClaire ('96)	no	no	no	no	na	no

Li W. Y. ('16)	no	yes	no	na	na	no
Li H. R. ('23)	no	no	no	no	na	no
Liu ('24)	no	no	no	na	na	no
Micheva-Viteva ('19)	no	no	no	no	na	no
Mo (2023)	yes	no	no	na	na	no
Nagai ('94)	no	no	no	no	na	no
Pacl ('23)	no	no	no	na	na	no
Park ('23)	yes	yes	no	no	yes	no
Pulido ('99)	no	no	no	na	na	no
Roboon ('21)	no	no	no	na	na	no
Rodriguez-C-B ('18)	no	no	no	no	na	no
Scharte ('03)	no	no	no	na	na	no
Selli ('23)	no	no	no	na	na	no
Shaw ('96)	no	no	no	no	na	na
Shi ('17)	no	no	no	na	na	no
Smith ('77)	no	no	no	no	na	na
Tian ('23)	no	no	no	na	no	no
Umpathy ('12)	no	no	no	na	na	no
Wray ('98)	no	no	no	no	na	no
Wurtele ('10)	no	no	no	na	na	no
Xing ('19)	no	no	no	no	na	na
Xu ('14)	no	no	no	na	no	no
Yan ('22)	no	no	no	no	na	no
Ye ('22)	no	yes	no	no	na	no
Yuan ('12)	no	no	no	no	na	no
Zhao ('23)	no	yes	no	no	no	no
Zingarelli ('96)	no	no	no	na	na	no

na – not applicable; uc – unclear;
