# Supplementary material for: Meta-analysis of niacin and NAD metabolite treatment in infectious disease animal studies suggests benefit but requires confirmation in clinically relevant models
Source: Sci Rep. 2025 Apr 12;15:12621. doi: 10.1038/s41598-025-95735-y (PMC11993703; doi:10.1038/s41598-025-95735-y)
Supplement: Supplementary file 34 — Supplementary Information 34. [file 41598_2025_95735_MOESM34_ESM.pdf]

Supplementary File-2. Supplementary Materials

Title of Review:

Database: PubMed/MEDLINE
Platform: US National Library of Medicine
Date Searched: March 4, 2024
Database Date Coverage: 1946–present
Date Limits: None
Other Limits/Filters: Language: English only; Subset: MEDLINE

Set	Concept	Search Strategy
#1	Niacin	("vitamin B3"[Title/Abstract] OR niacinamide*[Title/Abstract] OR nicotinamide*[Title/Abstract] OR "vitamin PP"[Title/Abstract] OR "vitamin B 3"[Title/Abstract] OR "3 Pyridinecarboxamide"[Title/Abstract] OR "3-Pyridinecarboxamide"[Title/Abstract] OR Enduramide[Title/Abstract] OR Nicobion[Title/Abstract] OR "Niacinamide"[Mesh] OR niacin[Title/Abstract] OR "Niacin"[Mesh] OR "nicotinic acid*"[Title/Abstract] OR "3 pyridine carboxylic acid"[tiab:~0] OR "3 pyridinecarboxylic acid*"[Title/Abstract] OR "lithium nicotinate*"[Title/Abstract] OR "nicotinamide riboside*"[Title/Abstract] OR "nicotinamide ribose*"[Title/Abstract] OR "nicotinamide-beta-riboside"[Supplementary Concept] OR "nicotinamide dinucleotide*"[Title/Abstract] OR "nicotinamide adenine dinucleotide*"[Title/Abstract] OR "Diphosphopyridine Nucleotide*"[Title/Abstract] OR "dihydronicotinamide adenine dinucleotide*"[Title/Abstract] OR nadide[Title/Abstract] OR NADH[Title/Abstract] OR "Coenzyme I"[Title/Abstract] OR "coenzyme i"[Title/Abstract] OR ((NAD[Title/Abstract] OR NAD+[Title/Abstract]) AND niacin[Title/Abstract]) OR ((NAD[Title/Abstract] OR NAD+[Title/Abstract]) AND "vitamin B3"[Title/Abstract]) OR ((NAD[Title/Abstract] OR NAD+[Title/Abstract]) AND "vitamin B 3"[Title/Abstract]) OR "NAD"[Mesh] OR "nicotinamide mononucleotide*"[Title/Abstract] OR "Nicotinamide Mononucleotide"[Mesh] OR "nicotinamide nucleotide*"[Title/Abstract])
#2	Infection	(sepsis[Majr] OR sepsis[Title/Abstract] OR septic[Title/Abstract] OR septicemia[Title/Abstract] OR septicaemia[Title/Abstract] OR "blood stream infection*"[Title/Abstract] OR pyemia*[Title/Abstract] OR pyaemia*[Title/Abstract] OR "blood poisoning*"[Title/Abstract] OR bacteremia[Title/Abstract] OR bacteraemia[Title/Abstract] OR fungemia[Title/Abstract] OR fungaemia[Title/Abstract] OR parasitemia[Title/Abstract] OR parasitaemia[Title/Abstract] OR viremia[Title/Abstract] OR viraemia[Title/Abstract] OR bacterial infections[Majr] OR "bacterial infection"[Title/Abstract] OR "bacterial infections"[Title/Abstract] OR critical illness[Majr] OR "critical illness"[Title/Abstract] OR "critical illnesses"[Title/Abstract] OR "intensive care"[Title/Abstract] OR "critically

		ill"[Title/Abstract] OR critical care[Majr] OR "critical care"[Title/Abstract] OR endotoxins[Majr] OR endotoxin[Title/Abstract] OR endotoxins[Title/Abstract] OR endotoxemia[Majr] OR endotoxemia[Title/Abstract] OR endotoxaemia[Title/Abstract] OR endotoxinemia[Title/Abstract] OR lipopolysaccharides[Majr] OR lipopolysaccharide[Title/Abstract] OR lipopolysaccharides[Title/Abstract] OR "systemic inflammation"[Title/Abstract] OR "systemic inflammatory"[Title/Abstract] OR "Bacterial Infections and Mycoses"[Majr] OR "Virus Diseases"[Majr] OR "viral infection*"[Title/Abstract] OR "virus infection*"[Title/Abstract] OR Infections[Mesh])
#3	Animal Models	("animal experimentation"[Mesh] OR "animal experiment*"[Title/Abstract] OR "models, animal"[Mesh] OR "animal model*"[Title/Abstract] OR "animal disease model*"[Title/Abstract] OR "Animals, Laboratory"[Mesh] OR "disease models, animal"[Mesh] OR "eulipotyphla"[Mesh] OR "Animals"[Mesh:noexp] OR animals[Title/Abstract] OR animal[Title/Abstract] OR "non human primate*"[Title/Abstract] OR "non-human primate*"[Title/Abstract] OR "nonhuman primate*"[Title/Abstract] OR "animal population groups"[Mesh] OR "vertebrates"[Mesh:noexp] OR "mammals"[Mesh:noexp] OR "primates"[Mesh:noexp] OR "artiodactyla"[Mesh] OR "carnivora"[Mesh] OR "elephants"[Mesh] OR "hyraxes"[Mesh] OR "lagomorpha"[Mesh] OR "marsupialia"[Mesh] OR "monotremata"[Mesh] OR "perissodactyla"[Mesh] OR "rodentia"[Mesh] OR "scandentia"[Mesh] OR "xenarthra"[Mesh] OR "haplorhini"[Mesh:noexp] OR "strepsirhini"[Mesh] OR "platyrrhini"[Mesh] OR "catarrhini"[Mesh:noexp] OR "cercopithecidae"[Mesh] OR "hylobatidae"[Mesh] OR "hominidae"[Mesh:noexp] OR "gorilla gorilla"[Mesh] OR "pan paniscus"[Mesh] OR "pan troglodytes"[Mesh] OR "pongo pygmaeus"[Mesh] OR mice[Title/Abstract] OR mus[Title/Abstract] OR mouse[Title/Abstract] OR murine[Title/Abstract] OR woodmouse[Title/Abstract] OR rats[Title/Abstract] OR rat[Title/Abstract] OR murinae[Title/Abstract] OR muridae[Title/Abstract] OR cottonrat[Title/Abstract] OR cottonrats[Title/Abstract] OR hamster[Title/Abstract] OR hamsters[Title/Abstract] OR cricetinae[Title/Abstract] OR rodentia[Title/Abstract] OR rodent[Title/Abstract] OR rodents[Title/Abstract] OR pigs[Title/Abstract] OR pig[Title/Abstract] OR minipig*[Title/Abstract] OR swine[Title/Abstract] OR swines[Title/Abstract] OR piglets[Title/Abstract] OR piglet[Title/Abstract] OR boar[Title/Abstract] OR boars[Title/Abstract] OR "sus scrofa"[Title/Abstract] OR ferrets[Title/Abstract] OR ferret[Title/Abstract] OR polecat[Title/Abstract] OR polecats[Title/Abstract] OR "mustela putorius"[Title/Abstract] OR "guinea pigs"[Title/Abstract] OR "guinea pig"[Title/Abstract] OR cavia[Title/Abstract] OR callithrix[Title/Abstract] OR marmoset[Title/Abstract] OR marmosets[Title/Abstract] OR cebuella[Title/Abstract] OR hapale[Title/Abstract] OR octodon[Title/Abstract] OR chinchilla[Title/Abstract] OR chinchillas[Title/Abstract] OR gerbillinae[Title/Abstract] OR gerbil[Title/Abstract] OR gerbils[Title/Abstract] OR jird[Title/Abstract] OR jirds[Title/Abstract] OR rabbits[Title/Abstract] OR rabbit[Title/Abstract] OR hares[Title/Abstract] OR hare[Title/Abstract] OR cats[Title/Abstract] OR cat[Title/Abstract] OR felis[Title/Abstract] OR

		feline*[Title/Abstract] OR dogs[Title/Abstract] OR dog[Title/Abstract] OR canine[Title/Abstract] OR canines[Title/Abstract] OR canis[Title/Abstract] OR sheep[Title/Abstract] OR sheeps[Title/Abstract] OR mouflon[Title/Abstract] OR mouflons[Title/Abstract] OR ovis[Title/Abstract] OR goats[Title/Abstract] OR goat[Title/Abstract] OR capra[Title/Abstract] OR capras[Title/Abstract] OR chamois[Title/Abstract] OR haplorhini[Title/Abstract] OR monkey[Title/Abstract] OR monkeys[Title/Abstract] OR anthropoidea[Title/Abstract] OR anthropoids[Title/Abstract] OR saguinus[Title/Abstract] OR tamarin[Title/Abstract] OR tamarins[Title/Abstract] OR leontopithecus[Title/Abstract] OR hominidae[Title/Abstract] OR ape[Title/Abstract] OR apes[Title/Abstract] OR "pan paniscus"[Title/Abstract] OR bonobo[Title/Abstract] OR bonobos[Title/Abstract] OR "pan troglodytes"[Title/Abstract] OR gibbon[Title/Abstract] OR gibbons[Title/Abstract] OR siamang[Title/Abstract] OR siamangs[Title/Abstract] OR nomascus[Title/Abstract] OR symphalangus[Title/Abstract] OR chimpanzee[Title/Abstract] OR chimpanzees[Title/Abstract] OR prosimian[Title/Abstract] OR prosimians[Title/Abstract] OR "bush baby"[Title/Abstract] OR "bush babies"[Title/Abstract] OR galagos[Title/Abstract] OR galago[Title/Abstract] OR pongidae[Title/Abstract] OR gorilla[Title/Abstract] OR gorillas[Title/Abstract] OR "pongo pygmaeus"[Title/Abstract] OR orangutan[Title/Abstract] OR orangutans[Title/Abstract] OR lemur[Title/Abstract] OR lemurs[Title/Abstract] OR lemuridae[Title/Abstract] OR horse[Title/Abstract] OR horses[Title/Abstract] OR equus[Title/Abstract] OR equine*[Title/Abstract] OR cow[Title/Abstract] OR cows[Title/Abstract] OR calf[Title/Abstract] OR calves[Title/Abstract] OR bovine*[Title/Abstract] OR bull[Title/Abstract] OR sciuridae[Title/Abstract] OR squirrel[Title/Abstract] OR squirrels[Title/Abstract] OR chipmunk[Title/Abstract] OR chipmunks[Title/Abstract] OR suslik[Title/Abstract] OR susliks[Title/Abstract] OR vole[Title/Abstract] OR voles[Title/Abstract] OR lemming[Title/Abstract] OR lemmings[Title/Abstract] OR muskrat[Title/Abstract] OR muskrats[Title/Abstract] OR lemmus[Title/Abstract] OR otter[Title/Abstract] OR otters[Title/Abstract] OR marten[Title/Abstract] OR martens[Title/Abstract] OR martes[Title/Abstract] OR weasel[Title/Abstract] OR badger[Title/Abstract] OR badgers[Title/Abstract] OR ermine[Title/Abstract] OR mink[Title/Abstract] OR minks[Title/Abstract] OR sable[Title/Abstract] OR sables[Title/Abstract] OR gulo[Title/Abstract] OR gulos[Title/Abstract] OR wolverine[Title/Abstract] OR wolverines[Title/Abstract] OR mustela[Title/Abstract] OR llama[Title/Abstract] OR llamas[Title/Abstract] OR alpaca[Title/Abstract] OR alpacas[Title/Abstract] OR camelid[Title/Abstract] OR camelids[Title/Abstract] OR guanaco[Title/Abstract] OR guanacos[Title/Abstract] OR foxes[Title/Abstract] OR fox[Title/Abstract] OR donkey[Title/Abstract] OR donkeys[Title/Abstract] OR mule[Title/Abstract] OR mules[Title/Abstract] OR zebra[Title/Abstract] OR zebras[Title/Abstract] OR shrew[Title/Abstract] OR shrews[Title/Abstract] OR bison[Title/Abstract] OR bisons[Title/Abstract] OR buffalo[Title/Abstract] OR buffaloes[Title/Abstract] OR deer[Title/Abstract] OR deers[Title/Abstract] OR bear[Title/Abstract] OR

		bears[Title/Abstract] OR panda[Title/Abstract] OR pandas[Title/Abstract] OR "wild hog"[Title/Abstract] OR "wild hogs"[Title/Abstract] OR "wild boars"[Title/Abstract] OR "wild boar"[Title/Abstract] OR fitchew[Title/Abstract] OR fitch[Title/Abstract] OR beaver[Title/Abstract] OR beavers[Title/Abstract] OR jerboa[Title/Abstract] OR jerboas[Title/Abstract] OR capybara[Title/Abstract] OR capybaras[Title/Abstract])
#4		#1 AND #2 AND #3
#5	Limit Applied: Subset and Language	#4 AND medline[subset] AND English[language]
#6	Limit Applied: Exclude Publication Types	#5 NOT ("retracted publication"[Publication Type] OR "retraction of publication"[Publication Type] OR "retraction of publication"[Title/Abstract] OR "retraction notice"[Title] OR "retracted publication"[Title/Abstract] OR "Preprint"[Publication Type] OR letter[Publication Type] OR editorial[Publication Type] OR comment[Publication Type] OR news[Publication Type] OR "Congress"[Publication Type] OR "Consensus Development Conference"[Publication Type] OR editorial[Title/Abstract] OR commentary[Title/Abstract] OR "conference abstract*"[Title/Abstract] OR "conference proceeding*"[Title/Abstract] OR symposium*[Title/Abstract] OR "Published Erratum"[Publication Type] OR errata[Title/Abstract] OR erratum[Title/Abstract] OR corrigenda[Title/Abstract] OR corrigendum[Title/Abstract] OR protocol[Title] OR protocols[Title] OR "meta-analysis"[Title/Abstract] OR "meta-analyses"[Title/Abstract] OR metanalyses[Title/Abstract] OR metanalysis[Title/Abstract] OR metaanalyses[Title/Abstract] OR metaanalysis[Title/Abstract] OR "meta analyses"[Title/Abstract] OR "meta analysis"[Title/Abstract] OR "Review"[Publication Type] OR "systematic review*"[Title/Abstract] OR "Systematic Review"[Publication Type] OR "Meta-Analysis" [Publication Type] OR "Network Meta-Analysis"[Mesh] OR "integrative review"[Title/Abstract])

Notes: The limit for language (i.e., English) and to the MEDLINE subset were applied to the main search using the filters available in PubMed. The keywords were searched in the title and abstract fields in PubMed (i.e., [Title/Abstract]), and the controlled vocabulary terms are indicated with [Mesh], if the MeSH term was a major focus of the article as [Majr], or if the MeSH term was not exploded to automatically include all narrower terms this was indicated with [Mesh:Noexp]. Phrases were enclosed in quotation marks to force the searching of the exact terms in order presented. No other limits were applied to the searches.

Database: Web of Science: Core Collection*
Platform: Clarivate Analytics
Date Searched: March 4, 2024
Database Date Coverage: 1900–present
Date Limits: None
Other Limits/Filters: Language: English

Set	Concept	Search Strategy
#1	Niacin	TS=("vitamin B3" OR niacinamide* OR nicotinamide* OR "vitamin PP" OR "vitamin B 3" OR "3 Pyridinecarboxamide" OR "3-Pyridinecarboxamide" OR Enduramide OR Nicobion OR niacin OR "nicotinic acid*" OR "3 pyridine carboxylic acid*" OR "3 pyridinecarboxylic acid*" OR "lithium nicotinate*" OR "nicotinamide riboside*" OR "nicotinamide ribose*" OR "nicotinamide dinucleotide*" OR "nicotinamide adenine dinucleotide*" OR "diphosphopyridine nucleotide*" OR "dihydronicotinamide adenine dinucleotide*" OR nadide OR NADH OR "Coenzyme I" OR "coenzyme i" OR ((NAD OR NAD+) AND niacin) OR ((NAD OR NAD+) AND "vitamin B3") OR ((NAD OR NAD+) AND "vitamin B 3") OR "nicotinamide mononucleotide*" OR "nicotinamide nucleotide*")
#2	Infection	TS=(sepsis OR septic OR septicemia OR septicaemia OR "blood stream infection*" OR pyemia* OR phyohemia* OR pyaemia* OR "blood poisoning*" OR bacteremia OR bacteraemia OR fungemia OR fungaemia OR parasitemia OR parasitaemia OR viremia OR viraemia OR "bacterial infection" OR "bacterial infections" OR "critical illness" OR "critical illnesses" OR "intensive care" OR "critically ill" OR "critical care" OR endotoxin OR endotoxins OR endotoxemia OR endotoxaemia OR endotoxinemia OR lipopolysaccharide OR lipopolysaccharides OR "systemic inflammation" OR "systemic inflammatory" OR "viral infection*" OR "virus infection*")
#3	Animal Models	TS=(animals OR animal OR mammal* OR "non-human primate*" OR "nonhuman primate*" OR "non human primate*" OR mice OR mus OR mouse OR murine OR woodmouse OR rats OR rat OR murinae OR muridae OR cottonrat OR cottonrats OR hamster OR hamsters OR cricetinae OR rodentia OR rodent OR rodents OR pigs OR pig OR swine OR swines OR piglets OR piglet OR boar OR boars OR "sus scrofa" OR ferrets OR ferret OR polecat OR polecats OR "mustela putorius" OR "guinea pigs" OR "guinea pig" OR cavia OR callithrix OR marmoset OR marmosets OR cebuella OR hapale OR octodon OR chinchilla OR chinchillas OR gerbillinae OR gerbil OR gerbils OR jird OR jirds OR rabbits OR rabbit OR hares OR hare OR cats OR cat OR felis OR feline* OR dogs OR dog OR canine OR canines OR canis OR sheep OR sheeps OR mouflon OR mouflons OR ovis OR goats OR goat OR capra OR capras OR rupicapra OR rupicapras OR chamois OR haplorhini OR monkey OR monkeys OR anthropoidea OR anthropoids OR saguinus OR tamarin OR tamarins OR leontopithecus OR hominidae OR ape OR apes OR "pan paniscus" OR bonobo OR bonobos OR "pan troglodytes" OR gibbon OR gibbons OR siamang OR siamangs OR nomascus OR symphalangus OR chimpanzee OR chimpanzees OR prosimian OR prosimians OR "bush baby" OR "bush babies" OR galagos OR galago OR pongidae OR gorilla OR gorillas OR

		"pongo pygmaeus" OR orangutan OR orangutans OR lemur OR lemurs OR lemuridae OR horse OR horses OR equus OR equine* OR cow OR calf OR bovine* OR bull OR sciuridae OR squirrel OR squirrels OR chipmunk OR chipmunks OR suslik OR susliks OR vole OR voles OR lemming OR lemmings OR muskrat OR muskrats OR lemmus OR otter OR otters OR marten OR martens OR martes OR weasel OR badger OR badgers OR ermine OR mink OR minks OR sable OR sables OR gulo OR gulos OR wolverine OR wolverines OR mustela OR llama OR llamas OR alpaca OR alpacas OR camelid OR camelids OR guanaco OR guanacos OR foxes OR donkey OR donkeys OR mule OR mules OR zebra OR zebras OR shrew OR shrews OR bison OR bisons OR buffalo OR buffaloes OR deer OR deers OR bear OR bears OR panda OR pandas OR "wild hog" OR "wild boar" OR fitchew OR fitch OR beaver OR beavers OR jerboa OR jerboas OR capybara OR capybaras)
#4		#1 AND #2 AND #3
#5	Limit Applied: Language	#4 AND (LA=("ENGLISH"))
#6	Limit applied: Exclude Publication Types	#5 NOT TI=(editorial OR commentary OR "conference abstract*" OR "conference proceeding*" OR symposium* OR errata OR erratum OR corrigenda OR corrigendum OR protocol OR protocols OR "meta-analysis" OR "meta-analyses" OR metanalyses OR metanalysis OR metaanalyses OR metaanalysis OR "meta analyses" OR "meta analysis" OR "systematic review*" OR "integrative review" OR retraction OR retracted OR "scoping review")
#7	Limit applied: Exclude Publication Types	#6 NOT DT=(Editorial Material OR Letter OR News Item OR Note OR Book OR Book Chapter OR Excerpt OR Item About an Individual OR Meeting Abstract OR Meeting Summary OR Reprint OR Review OR Retracted Publication OR Retraction)

Notes: The limit for language (i.e., English) was applied to the main search using the filter available. The keywords were searched in the Topic field (i.e., TS) which searches the title, abstract, author keywords, and KeyWordsPlus fields. Phrases were enclosed in double quotation marks to force the searching of the exact terms in order presented. The search terms to limit by publication type was searched in the Title field only (i.e., TI) and the Document Type field (i.e., DT). No other limits were applied to the searches.

*Science Citation Index Expanded (SCI-EXPANDED)--1900-present
Social Sciences Citation Index (SSCI)--1900-present
Conference Proceedings Citation Index – Science (CPCI-S)--1990-present
Conference Proceedings Citation Index – Social Science & Humanities (CPCI-SSH)--1990-present
Book Citation Index – Science (BKCI-S)--2005-present
Book Citation Index – Social Sciences & Humanities (BKCI-SSH)--2005-present
Emerging Sources Citation Index (ESCI)--2005-present
Current Chemical Reactions (CCR-EXPANDED)--1985-present

Index Chemicus (IC)--1993-present

Database: Web of Science: BIOSIS
Platform: Clarivate Analytics
Date Searched: March 4, 2024
Database Date Coverage: 1900–present
Date Limits: None
Other Limits/Filters: Language: English

Set	Concept	Search Strategy
#1	Niacin	TS=("vitamin B3" OR niacinamide* OR nicotinamide* OR "vitamin PP" OR "vitamin B 3" OR "3 Pyridinecarboxamide" OR "3-Pyridinecarboxamide" OR Enduramide OR Nicobion OR niacin OR "nicotinic acid*" OR "3 pyridine carboxylic acid*" OR "3 pyridinecarboxylic acid*" OR "lithium nicotinate*" OR "nicotinamide riboside*" OR "nicotinamide ribose*" OR "nicotinamide dinucleotide*" OR "nicotinamide adenine dinucleotide*" OR "diphosphopyridine nucleotide*" OR "dihydronicotinamide adenine dinucleotide*" OR nadide OR NADH OR "Coenzyme I" OR "coenzyme i" OR ((NAD OR NAD+) AND niacin) OR ((NAD OR NAD+) AND "vitamin B3") OR ((NAD OR NAD+) AND "vitamin B 3") OR "nicotinamide mononucleotide*" OR "nicotinamide nucleotide*")
#2	Infection	TS=(sepsis OR septic OR septicemia OR septicaemia OR "blood stream infection*" OR pyemia* OR phyohemia* OR pyaemia* OR "blood poisoning*" OR bacteremia OR bacteraemia OR fungemia OR fungaemia OR parasitemia OR parasitaemia OR viremia OR viraemia OR "bacterial infection" OR "bacterial infections" OR "critical illness" OR "critical illnesses" OR "intensive care" OR "critically ill" OR "critical care" OR endotoxin OR endotoxins OR endotoxemia OR endotoxaemia OR endotoxinemia OR lipopolysaccharide OR lipopolysaccharides OR "systemic inflammation" OR "systemic inflammatory" OR "viral infection*" OR "virus infection*")
#3	Animal Models	TS=(animals OR animal OR mammal* OR "non-human primate*" OR "nonhuman primate*" OR "non human primate*" OR mice OR mus OR mouse OR murine OR woodmouse OR rats OR rat OR murinae OR muridae OR cottonrat OR cottonrats OR hamster OR hamsters OR cricetinae OR rodentia OR rodent OR rodents OR pigs OR pig OR swine OR swines OR piglets OR piglet OR boar OR boars OR "sus scrofa" OR ferrets OR ferret OR polecat OR polecats OR "mustela putorius" OR "guinea pigs" OR "guinea pig" OR cavia OR callithrix OR marmoset OR marmosets OR cebuella OR hapale OR octodon OR chinchilla OR chinchillas OR gerbillinae OR gerbil OR gerbils OR jird OR jirds OR rabbits OR rabbit OR hares OR hare OR cats OR cat OR felis OR feline* OR dogs OR dog OR canine OR canines OR canis OR sheep OR sheeps OR mouflon OR mouflons OR ovis OR goats OR goat OR capra OR capras OR rupicapra OR rupicapras OR chamois OR haplorhini OR monkey OR monkeys OR anthropoidea OR anthropoids OR saguinus OR tamarin OR tamarins OR leontopithecus OR hominidae OR ape OR apes OR "pan paniscus" OR bonobo OR bonobos OR "pan troglodytes" OR gibbon OR gibbons OR siamang OR siamangs OR nomascus OR symphalangus OR chimpanzee OR chimpanzees OR prosimian OR prosimians OR "bush baby" OR "bush babies" OR galagos OR galago OR pongidae OR gorilla OR gorillas OR

		"pongo pygmaeus" OR orangutan OR orangutans OR lemur OR lemurs OR lemuridae OR horse OR horses OR equus OR equine* OR cow OR calf OR bovine* OR bull OR sciuridae OR squirrel OR squirrels OR chipmunk OR chipmunks OR suslik OR susliks OR vole OR voles OR lemming OR lemmings OR muskrat OR muskrats OR lemmus OR otter OR otters OR marten OR martens OR martes OR weasel OR badger OR badgers OR ermine OR mink OR minks OR sable OR sables OR gulo OR gulos OR wolverine OR wolverines OR mustela OR llama OR llamas OR alpaca OR alpacas OR camelid OR camelids OR guanaco OR guanacos OR foxes OR donkey OR donkeys OR mule OR mules OR zebra OR zebras OR shrew OR shrews OR bison OR bisons OR buffalo OR buffaloes OR deer OR deers OR bear OR bears OR panda OR pandas OR "wild hog" OR "wild boar" OR fitchew OR fitch OR beaver OR beavers OR jerboa OR jerboas OR capybara OR capybaras)
#4		#1 AND #2 AND #3
#5	Limit Applied: Language	#4 AND (LA=("ENGLISH"))
#6	Limit applied: Exclude Publication Types	#5 NOT TI=(editorial OR commentary OR "conference abstract*" OR "conference proceeding*" OR symposium* OR errata OR erratum OR corrigenda OR corrigendum OR protocol OR protocols OR "meta-analysis" OR "meta-analyses" OR metanalyses OR metanalysis OR metaanalyses OR metaanalysis OR "meta analyses" OR "meta analysis" OR "systematic review*" OR "integrative review" OR retraction OR retracted OR "scoping review")
#7	Limit applied: Exclude Publication Types	#6 NOT DT=(Editorial Material OR Letter OR News Item OR Note OR Book OR Book Chapter OR Excerpt OR Item About an Individual OR Meeting Abstract OR Meeting Summary OR Reprint OR Review OR Retracted Publication OR Retraction)

Notes: The limit for language (i.e., English) was applied to the main search using the filter available. The keywords were searched in the Topic field (i.e., TS) which searches the title, abstract, author keywords, and KeyWordsPlus fields. Phrases were enclosed in double quotation marks to force the searching of the exact terms in order presented. The search terms to limit by publication type was searched in the Title field only (i.e., TI) and the Document Type field (i.e., DT). No other limits were applied to the searches.

Database: Scopus
Platform: Elsevier
Date Searched: March 4, 2024
Database Date Coverage: 1788–present
Date Limits: None
Other Limits/Filters: Language: English

Set	Concept	Search Strategy
#1	Niacin	TITLE-ABS-KEY({vitamin B3} OR niacinamide* OR nicotinamide* OR {vitamin PP} OR {vitamin B 3} OR {3 Pyridinecarboxamide} OR {3-Pyridinecarboxamide} OR Enduramide OR Nicobion OR niacin OR {nicotinic acid*} OR {3 pyridine carboxylic acid*} OR {3 pyridinecarboxylic acid*} OR {lithium nicotinate*} OR {nicotinamide riboside*} OR {nicotinamide ribose*} OR {nicotinamide dinucleotide*} OR {nicotinamide adenine dinucleotide*} OR {diphosphopyridine nucleotide*} OR {dihydronicotinamide adenine dinucleotide*} OR nadide OR NADH OR {Coenzyme I} OR {coenzyme i} OR ((NAD OR NAD+) AND niacin) OR ((NAD OR NAD+) AND {vitamin B3}) OR ((NAD OR NAD+) AND {vitamin B 3}) OR {nicotinamide mononucleotide*} OR {nicotinamide nucleotide*})
#2	Infection	TITLE-ABS-KEY(sepsis OR septic OR septicemia OR septicaemia OR {blood stream infection*} OR pyemia* OR phyohemia* OR pyaemia* OR {blood poisoning*} OR bacteremia OR bacteraemia OR fungemia OR fungaemia OR parasitemia OR parasitaemia OR viremia OR viraemia OR {bacterial infection} OR {bacterial infections} OR {critical illness} OR {critical illnesses} OR {intensive care} OR {critically ill} OR {critical care} OR endotoxin OR endotoxins OR endotoxemia OR endotoxaemia OR endotoxinemia OR lipopolysaccharide OR lipopolysaccharides OR {systemic inflammation} OR {systemic inflammatory} OR {viral infection*} OR {virus infection*})
#3	Animal Models	TITLE-ABS-KEY(animals OR animal OR mammal* OR {non-human primate*} OR {nonhuman primate*} OR {non human primate*} OR mice OR mus OR mouse OR murine OR woodmouse OR rats OR rat OR murinae OR muridae OR cottonrat OR cottonrats OR hamster OR hamsters OR cricetinae OR rodentia OR rodent OR rodents OR pigs OR pig OR swine OR swines OR piglets OR piglet OR boar OR boars OR {sus scrofa} OR ferrets OR ferret OR polecat OR polecats OR {mustela putorius} OR {guinea pigs} OR {guinea pig} OR cavia OR callithrix OR marmoset OR marmosets OR cebuella OR hapale OR octodon OR chinchilla OR chinchillas OR gerbillinae OR gerbil OR gerbils OR jird OR jirds OR rabbits OR rabbit OR hares OR hare OR cats OR cat OR felis OR feline* OR dogs OR dog OR canine OR canines OR canis OR sheep OR sheeps OR mouflon OR mouflons OR ovis OR goats OR goat OR capra OR capras OR rupicapra OR rupicapras OR chamois OR haplorhini OR monkey OR monkeys OR anthropoidea OR anthropoids OR saguinus OR tamarin OR tamarins OR leontopithecus OR hominidae OR ape OR apes OR {pan paniscus} OR bonobo OR bonobos OR {pan troglodytes} OR gibbon OR gibbons OR siamang OR siamangs OR nomascus OR symphalangus OR chimpanzee OR chimpanzees OR prosimian OR prosimians OR {bush baby} OR {bush babies} OR galagos OR galago OR pongidae OR gorilla OR gorillas OR

		{pongo pygmaeus} OR orangutan OR orangutans OR lemur OR lemurs OR lemuridae OR horse OR horses OR equus OR equine* OR cow OR calf OR bovine* OR bull OR sciuridae OR squirrel OR squirrels OR chipmunk OR chipmunks OR suslik OR susliks OR vole OR voles OR lemming OR lemmings OR muskrat OR muskrats OR lemmus OR otter OR otters OR marten OR martens OR martes OR weasel OR badger OR badgers OR ermine OR mink OR minks OR sable OR sables OR gulo OR gulos OR wolverine OR wolverines OR mustela OR llama OR llamas OR alpaca OR alpacas OR camelid OR camelids OR guanaco OR guanacos OR foxes OR donkey OR donkeys OR mule OR mules OR zebra OR zebras OR shrew OR shrews OR bison OR bisons OR buffalo OR buffaloes OR deer OR deers OR bear OR bears OR panda OR pandas OR {wild hog} OR {wild boar} OR fitchew OR fitch OR beaver OR beavers OR jerboa OR jerboas OR capybara OR capybaras))
#4		#1 AND #2 AND #3
#5	Limit Applied: Language	#4 AND (LIMIT-TO (LANGUAGE , "English"))
#6	Limit Applied: Exclude Publication Type	#5 AND NOT DOCTYPE(ab OR bk OR ch OR cp OR cr OR bz OR dp OR ed OR er OR le OR mm OR no OR pr OR rp OR re OR sh)
#7	Limit Applied: Exclude Publication Type	#6 AND NOT TITLE(editorial OR commentary OR {conference abstract*} OR {conference proceeding*} OR symposium* OR errata OR erratum OR corrigenda OR corrigendum OR protocol OR protocols OR {meta-analysis} OR {meta-analyses} OR metanalyses OR metanalysis OR metaanalyses OR metaanalysis OR {meta analyses} OR {meta analysis} OR {systematic review*} OR {integrative review})

Notes: The limit for language (i.e., English) was applied to the main search using the filter available. The keywords were searched in the title, abstract, or keywords fields. Phrases were enclosed in curly brackets (i.e., { }) to force the searching of the exact terms in order presented, or double quotation marks (i.e., “x”) to find approximate phrases. The search terms to limit by publication type was searched in the Title field only (i.e., TITLE) and the Document Type field (i.e., DOCTYPE). No other limits were applied to the searches.

Database: Embase
Platform: Elsevier
Date Searched: March 4, 2024
Database Date Coverage: 1947–present
Date Limits: None
Other Limits/Filters: Source: Embase & Embase Classic; Language: English

Set	Concept	Search Strategy
#1	Niacin	('vitamin B3':ti,ab OR niacinamide*:ti,ab OR nicotinamide*:ti,ab OR 'vitamin PP':ti,ab OR 'vitamin B 3':ti,ab OR '3 Pyridinecarboxamide':ti,ab OR '3-Pyridinecarboxamide':ti,ab OR Enduramide:ti,ab OR Nicobion:ti,ab OR niacin:ti,ab OR 'nicotinic acid*':ti,ab OR '3 pyridine carboxylic acid*':ti,ab OR '3 pyridinecarboxylic acid*':ti,ab OR 'lithium nicotinate*':ti,ab OR 'nicotinamide riboside*':ti,ab OR 'nicotinamide ribose*':ti,ab OR 'nicotinamide dinucleotide*':ti,ab OR 'nicotinamide adenine dinucleotide*':ti,ab OR 'diphosphopyridine nucleotide*':ti,ab OR 'dihydronicotinamide adenine dinucleotide*':ti,ab OR nadide:ti,ab OR NADH:ti,ab OR 'Coenzyme I':ti,ab OR 'coenzyme i':ti,ab OR ((NAD:ti,ab OR NAD+:ti,ab) AND niacin:ti,ab) OR ((NAD:ti,ab OR NAD+:ti,ab) AND 'vitamin B3':ti,ab) OR ((NAD:ti,ab OR NAD+:ti,ab) AND 'vitamin B 3':ti,ab) OR 'nicotinamide mononucleotide*':ti,ab OR 'nicotinamide nucleotide*':ti,ab OR 'nicotinamide'/exp OR 'nicotinic acid'/exp OR 'nicotinamide riboside'/exp OR 'nicotinamide adenine dinucleotide'/exp OR 'nicotinamide nucleotide'/exp OR 'reduced nicotinamide adenine dinucleotide'/exp)
#2	Infection	(sepsis:ti,ab OR septic:ti,ab OR septicemia:ti,ab OR septicaemia:ti,ab OR 'blood stream infection*':ti,ab OR pyemia*:ti,ab OR phyohemia*:ti,ab OR pyaemia*:ti,ab OR 'blood poisoning*':ti,ab OR bacteremia:ti,ab OR bacteraemia:ti,ab OR fungemia:ti,ab OR fungaemia:ti,ab OR parasitemia:ti,ab OR viremia:ti,ab OR viraemia:ti,ab OR 'viral infection*':ti,ab OR 'virus infection*':ti,ab OR 'bacterial infection':ti,ab OR 'bacterial infections':ti,ab OR 'critical illness':ti,ab OR 'critical illnesses':ti,ab OR 'critically ill':ti,ab OR 'critical care':ti,ab OR 'intensive care':ti,ab OR endotoxin:ti,ab OR endotoxins:ti,ab OR endotoxemia:ti,ab OR endotoxaemia:ti,ab OR endotoxinemia:ti,ab OR lipopolysaccharide:ti,ab OR lipopolysaccharides:ti,ab OR 'systemic inflammation':ti,ab OR 'systemic inflammatory':ti,ab OR 'sepsis'/exp/mj OR 'septicemia'/exp/mj OR 'septic shock'/exp/mj OR 'bacteremia'/exp/mj OR 'fungemia'/exp/mj OR 'bacterial infection'/exp/mj OR 'infection'/exp OR 'virus infection'/exp/mj OR 'viremia'/exp/mj OR 'critical illness'/exp/mj OR 'intensive care'/exp/mj OR 'endotoxin'/exp/mj OR 'endotoxemia'/exp/mj OR 'lipopolysaccharide'/exp/mj OR 'systemic inflammation'/exp/mj)
#3	Animal Models	('animal'/de OR animals:ti,ab OR animal:ti,ab OR 'animal model'/exp OR 'animal experiment'/exp OR [animal cell]/lim OR [animal experiment]/lim OR [animal model]/lim OR [animal tissue]/lim OR 'invertebrate'/exp OR 'vertebrate'/de OR 'mammal'/de OR mammal*:ti,ab OR 'primate'/de OR 'non-human primate*':ti,ab OR 'non human primate*':ti,ab OR 'nonhuman primate*':ti,ab OR 'Artiodactyla'/exp OR 'Carnivora'/exp OR 'elephant'/exp OR 'hyrax'/exp OR

		'Insectivora'/exp OR 'lagomorph'/exp OR 'marsupial'/exp OR 'Platyrrhini'/exp OR 'Catarrhini'/de OR 'monotreme'/exp OR 'Perissodactyla'/exp OR 'rodent'/exp OR 'Scandentia'/exp OR 'Xenarthra'/exp OR 'Haplorhini'/de OR 'prosimian'/exp OR 'Cercopithecidae'/exp OR 'Hylobatidae'/exp OR 'ape'/de OR 'hominid'/de OR 'gorilla'/exp OR orangutan/exp OR chimpanzee/exp OR 'Pan paniscus'/exp OR 'Pan troglodytes'/exp OR 'Pongo pygmaeus'/exp OR mice:ti,ab OR mus:ti,ab OR mouse:ti,ab OR murine:ti,ab OR woodmouse:ti,ab OR rats:ti,ab OR rat:ti,ab OR murinae:ti,ab OR muridae:ti,ab OR cottonrat:ti,ab OR cottonrats:ti,ab OR hamster:ti,ab OR hamsters:ti,ab OR cricetinae:ti,ab OR rodentia:ti,ab OR rodent:ti,ab OR rodents:ti,ab OR pigs:ti,ab OR pig:ti,ab OR swine:ti,ab OR swines:ti,ab OR piglets:ti,ab OR piglet:ti,ab OR boar:ti,ab OR boars:ti,ab OR 'sus scrofa':ti,ab OR ferrets:ti,ab OR ferret:ti,ab OR polecat:ti,ab OR polecats:ti,ab OR 'mustela putorius':ti,ab OR 'guinea pigs':ti,ab OR 'guinea pig':ti,ab OR cavia:ti,ab OR callithrix:ti,ab OR marmoset:ti,ab OR marmosets:ti,ab OR cebuella:ti,ab OR hapale:ti,ab OR octodon:ti,ab OR chinchilla:ti,ab OR chinchillas:ti,ab OR gerbillinae:ti,ab OR gerbil:ti,ab OR gerbils:ti,ab OR jird:ti,ab OR jirds:ti,ab OR rabbits:ti,ab OR rabbit:ti,ab OR hares:ti,ab OR hare:ti,ab OR cats:ti,ab OR cat:ti,ab OR felis:ti,ab OR feline*:ti,ab OR dogs:ti,ab OR dog:ti,ab OR canine:ti,ab OR canines:ti,ab OR canis:ti,ab OR sheep:ti,ab OR sheeps:ti,ab OR mouflon:ti,ab OR mouflons:ti,ab OR ovis:ti,ab OR goats:ti,ab OR goat:ti,ab OR capra:ti,ab OR capras:ti,ab OR rupicapra:ti,ab OR rupicapras:ti,ab OR chamois:ti,ab OR haplorhini:ti,ab OR monkey:ti,ab OR monkeys:ti,ab OR anthropoidea:ti,ab OR anthropoids:ti,ab OR saguinus:ti,ab OR tamarin:ti,ab OR tamarins:ti,ab OR leontopithecus:ti,ab OR hominidae:ti,ab OR ape:ti,ab OR apes:ti,ab OR 'pan paniscus':ti,ab OR bonobo:ti,ab OR bonobos:ti,ab OR 'pan troglodytes':ti,ab OR gibbon:ti,ab OR gibbons:ti,ab OR siamang:ti,ab OR siamangs:ti,ab OR nomascus:ti,ab OR symphalangus:ti,ab OR chimpanzee:ti,ab OR chimpanzees:ti,ab OR prosimian:ti,ab OR prosimians:ti,ab OR 'bush baby':ti,ab OR 'bush babies':ti,ab OR galagos:ti,ab OR galago:ti,ab OR pongidae:ti,ab OR gorilla:ti,ab OR gorillas:ti,ab OR 'pongo pygmaeus':ti,ab OR orangutan:ti,ab OR orangutans:ti,ab OR lemur:ti,ab OR lemurs:ti,ab OR lemuridae:ti,ab OR horse:ti,ab OR horses:ti,ab OR equus:ti,ab OR equine*:ti,ab OR cow:ti,ab OR calf:ti,ab OR bovine*:ti,ab OR bull:ti,ab OR sciuridae:ti,ab OR squirrel:ti,ab OR squirrels:ti,ab OR chipmunk:ti,ab OR chipmunks:ti,ab OR suslik:ti,ab OR susliks:ti,ab OR vole:ti,ab OR voles:ti,ab OR lemming:ti,ab OR lemmings:ti,ab OR muskrat:ti,ab OR muskrats:ti,ab OR lemmus:ti,ab OR otter:ti,ab OR otters:ti,ab OR marten:ti,ab OR martens:ti,ab OR martes:ti,ab OR weasel:ti,ab OR badger:ti,ab OR badgers:ti,ab OR ermine:ti,ab OR mink:ti,ab OR minks:ti,ab OR sable:ti,ab OR sables:ti,ab OR gulo:ti,ab OR gulos:ti,ab OR wolverine:ti,ab OR wolverines:ti,ab OR mustela:ti,ab OR llama:ti,ab OR llamas:ti,ab OR alpaca:ti,ab OR alpacas:ti,ab OR camelid:ti,ab OR camelids:ti,ab OR guanaco:ti,ab OR guanacos:ti,ab OR foxes:ti,ab OR donkey:ti,ab OR donkeys:ti,ab OR mule:ti,ab OR mules:ti,ab OR zebra:ti,ab OR zebras:ti,ab OR shrew:ti,ab OR shrews:ti,ab OR bison:ti,ab OR bisons:ti,ab OR buffalo:ti,ab OR buffaloes:ti,ab OR deer:ti,ab OR deers:ti,ab OR bear:ti,ab OR bears:ti,ab OR panda:ti,ab OR pandas:ti,ab OR 'wild hog':ti,ab OR 'wild boar':ti,ab

		OR fitchew:ti,ab OR fitch:ti,ab OR beaver:ti,ab OR beavers:ti,ab OR jerboa:ti,ab OR jerboas:ti,ab OR capybara:ti,ab OR capybaras:ti,ab)
#4		#1 AND #2 AND #3
#5	Limits Applied: Source and Language	#4 AND ([embase]/lim OR [embase classic]/lim) AND [english]/lim
#6	Limit Applied: Exclude Publication Types	#5 NOT ([conference abstract]/lim OR [conference paper]/lim OR [conference review]/lim OR [data papers]/lim OR [editorial]/lim OR [erratum]/lim OR [letter]/lim OR [note]/lim OR [review]/lim OR [short survey]/lim OR [systematic review]/lim OR [meta analysis]/lim OR [preprint]/lim OR 'conference abstract'/exp OR 'conference paper'/exp OR 'data paper'/exp OR 'editorial'/exp OR 'letter'/exp OR 'erratum'/exp OR 'note'/exp OR 'short survey'/exp OR 'review'/exp OR 'systematic review'/exp OR 'meta analysis'/exp OR 'retraction notice'/exp OR 'retraction notice':ti OR 'retracted publication':ti,ab OR 'retraction of publication':ti,ab OR corrigenda:ti,ab OR corrigendum:ti,ab OR erratum:ti,ab OR errata:ti,ab OR 'conference abstract*':ti,ab OR 'conference proceeding*':ti,ab OR symposium*:ti,ab OR letter:ti,ab OR editorial:ti,ab OR commentary:ti,ab OR 'meta analysis':ti,ab OR 'meta analyses':ti,ab OR metaanalysis:ti,ab OR metaanalyses:ti,ab OR metanalysis:ti,ab OR metanalyses:ti,ab OR 'meta-analysis':ti,ab OR 'meta-analyses':ti,ab OR 'systematic review*':ti,ab OR 'integrative review*':ti,ab)

Notes: The limits for language (i.e., English) and source (Embase and Embase Classic) were applied to the main search using the filters available in Embase. The keywords were searched in the title and abstract fields (i.e., :ti,ab), and the EMTREE controlled vocabulary terms were searched as /de or if the EMTREE term was exploded to automatically include all narrower terms this was indicated with /exp. EMTREE terms searched as the major focus of the article are indicated with /mj. Phrases were enclosed in single quotation marks to force the searching of the exact terms in order presented. No other limits were applied to the searches.
