# Supplementary material for: Meta-analysis of niacin and NAD metabolite treatment in infectious disease animal studies suggests benefit but requires confirmation in clinically relevant models
Source: Sci Rep. 2025 Apr 12;15:12621. doi: 10.1038/s41598-025-95735-y (PMC11993703; doi:10.1038/s41598-025-95735-y)
Supplement: Supplementary file 35 — Supplementary Information 35. [file 41598_2025_95735_MOESM35_ESM.pdf]

Supplementary File-3

Information Sources and Search Strategy

A biomedical librarian searched five bibliographic databases: Embase (Elsevier), PubMed/MEDLINE (US National Library of Medicine), Scopus (Elsevier), Web of Science: BIOSIS Citation Index (Clarivate Analytics), and Web of Science: Core Collection (Clarivate Analytics) for both animal and human studies. The searches were completed in December 2022 and updated in February 2024. A combination of keywords and controlled vocabulary terms (i.e., EMTREE [Embase] and MeSH [PubMed/MEDLINE]) were used for each concept of interest (i.e., niacin/vitamin B3, infection/sepsis, animals). The review team provided feedback on the search strategies. The searches were limited to those published in English, and search strategies were used to exclude specific article types included in the exclusion criteria.

The librarian used EndNote 21 (Clarivate Analytics) to collect the search results from each database, identify and remove duplicates, and export the unique records for screening.
